# Applying Augmented Reality to Convey Medical Knowledge on Osteoclasts to Users of a Serious Game: Vignette Experiment

**DOI:** 10.2196/64751

**Published:** 2025-06-16

**Authors:** Jascha Grübel, Julia Chatain, Claudio Schmid, Violaine Fayolle, Fabio Zünd, Reinhard Gruber, Bernd Stadlinger

**Affiliations:** 1 Laboratory for Geo-Information Science and Remote Sensing, Department of Environmental Sciences, Wageningen University, Droevendaalsesteeg 4, Wageningen, 6708 PB, The Netherlands, +41 44 632 51 54; 2 Center for Sustainable Future Mobility, Department of Mechanical and Process Engineering & Department of Civil, Environmental and Geomatic Engineering, ETH Zurich, Zürich, Switzerland; 3 Game Technology Center, Department of Computer Science, ETH Zurich, Zürich, Switzerland; 4 Chair of Cognitive Science, Department of Humanities, Social and Political Sciences, ETH Zurich, Zürich, Switzerland; 5 Visual Computing Group, Harvard John A. Paulson School Of Engineering And Applied Sciences, Harvard University, Boston, MA, United States; 6 Future Embodied Learning Technologies, Singapore-ETH Centre, Singapore, Singapore; 7 Center for Dental Medicine, Clinic of Cranio-Maxillofacial and Oral Surgery, University of Zurich, Zürich, Switzerland; 8 Disney Research, Zürich, Switzerland; 9 Competence Center of Oral Biology, University Clinic of Dentistry, Medical University of Vienna, Vienna, Austria; 10 ETH AI Center, Zürich, Switzerland

**Keywords:** tablet-based augmented reality, osteoclasts, educational game, user study, vignette experiment, augmented reality, communication, medical student, oral cavity, serious game

## Abstract

**Background:**

Visualization technology is enhancing interactive learning by merging digital content with real-world environments, offering immersive experiences through augmented reality (AR) in fields like medical education. AR is being increasingly used in medicine and dental education to improve student learning, particularly in understanding complex concepts such as bone remodeling. Active learning strategies, supported by AR, boost student autonomy, reduce cognitive load, and improve learning outcomes across various disciplines. AR is gaining popularity in higher education as it enhances active learning, reduces cognitive load, and improves cognitive, meta-cognitive, and affective outcomes, particularly in medical and nursing education. The effectiveness of immersive AR in enhancing understanding of complex physiological processes is still unclear, with a lack of rigorous studies on its impact and how to effectively convert academic content into AR.

**Objective:**

We assess the capacity of AR-enhanced content for learning medical knowledge with a state-of-the-art AR game published along with a modern cell atlas of the oral cavity. To assess AR-enhanced content for learning, we formulated hypotheses on the general impact on learning (H1), specific improvements in learning (H2), and long-term retention (H3).

**Methods:**

An AR serious game was developed to represent current knowledge on osteoclasts for classroom use. The game was evaluated in an unblinded face-to-face vignette experiment (39 participants). Learning outcomes on “Osteoclasts” were compared between the AR game (17 participants) and a textbook-only option (20 participants) conveying the same content. Participants were randomly assigned and learning success was measured at three time-points, immediately after the experiment session, 1 week later, and 1 month later, via web-based surveys.

**Results:**

The AR serious game elicited strong interest in the topic (perceived relevance in Attention, Relevance, Confidence, and Satisfaction [ARCS], W=10,417; *P*<.001) and motivated students by increasing self-efficacy (confidence in ARCS, W=11,882.5; *P*=.02) and satisfaction (in ARCS, W=4561; *P*<.001). The learning outcomes were comparable to text-based self-learning (*t*=2.0103; *P*_Bonferroni_=.095). Furthermore, curious students benefited more from interactive learning methods compared with text-only methods and had higher learning success (*t*=−2.518; *P*=.02).

**Conclusions:**

Introducing new technology such as AR into teaching requires technological investment, updated curricula, and careful application of learning paradigms. We found support for improved motivation (H1) and some evidence of AR’s baseline effectiveness (H2a). While we could not confirm AR’s impact on visual tasks overall (H2b), we noted an interesting interaction between curiosity and visual task outcomes (H2c), as well as how game design influences student perception of the material (H2d). Due to attrition, long-term learning outcomes (H3) could not be assessed. AR-based learning may particularly benefit curious students, who often struggle with text-heavy methods. As students are increasingly accustomed to brief, engaging content, teaching approaches must adapt.

## Introduction

**Figure 1.**
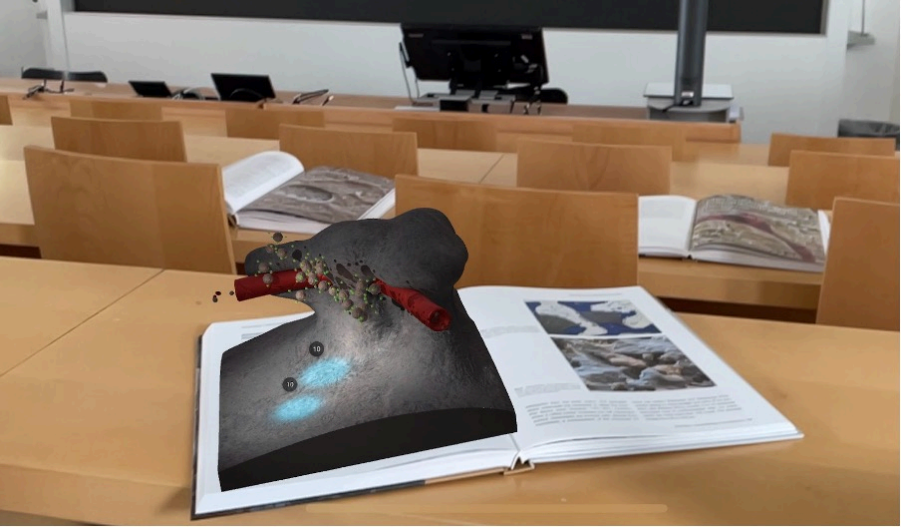
The augmented reality osteoclast serious game being played in a lecture hall.

Visualization technology is transforming interactive learning by merging digital content with the real world to enhance educational experiences [1]. Augmented reality (AR) superimposes computer-generated content over real-world environments, enriching sensory perception with 3D images, videos, and audio [2], enabling interaction in a mixed-reality space. AR integrates real environmental stimuli with digital representations [3,4], offering immersive media for fields like medical education (Figure 1). Examples of AR in teaching include augmenting books with digital content [5] and the augmentation of physical objects [6] with contextualized information. Since the 1990s, AR has evolved with advancements in hardware, software, and head-mounted displays (eg, Microsoft HoloLens), expanding its potential as a teaching tool [7]. The improvement of smartphones and tablets has further broadened AR’s influence [8], which is reflected in everyday applications like Snapchat filters, Pokémon Go, and IKEA Place [9].

AR is increasingly used in medicine and dental medicine to enhance student learning, particularly in understanding complex concepts. AR is already used for various applications, from patient communication to student learning and to overcome dental phobia [10,11]. This paper focuses on the potential of AR-enhanced learning material to help students [12] understand the complex concepts of bone remodeling. Knowledge of physiology is essential for teaching health sciences, including medicine and dentistry. Bone physiology is of key importance in surgical fields like orthopedics and oral, and maxillofacial surgery [13]. Many treatments, such as dental implant placement, depend on successful bone remodeling [14]. Currently, bone biology and remodeling are taught in medical school mainly through histology, where tissues are examined and discussed at the microscopic level [15]. Histology courses typically include lectures and microscopy labs, but students often struggle with understanding the spatial relationships of 2D tissue sections [16]. AR-enhanced learning materials could help students to better visualize and understand the spatial features of bone biology.

Active learning strategies, supported by AR technology, enhance student autonomy, reduce cognitive load, and improve learning outcomes across various disciplines. To understand AR-enhanced learning, we considered learning frameworks that emphasize student autonomy and agency [17], such as active learning strategies. Matching content with an appropriate medium is crucial for creating a coherent learning experience [1]. Over the past decades, active learning strategies, such as flipped classrooms [18], project-based learning [19], and productive failure [20], have been shown to improve understanding, long-term learning, ability to transfer understanding to other situations and contexts [21-24].

The use of AR in higher education is gaining attention because it can enhance active learning, reduce cognitive load, and improve both cognitive and affective outcomes. AR in higher education is being increasingly used [25] because it supports active forms of learning [26] by colocating information and its referent, reducing split-attention effects [27]. AR also fosters collaboration and supports social incentives for learning [28]. One potential pathway in which AR context could improve active learning is by reducing cognitive load through visualization [29,30]. AR game-based learning positively impacts not only cognitive outcomes (eg, performance and learning), but also meta-cognitive outcomes (eg, attitude and participation), as well as affective outcomes (eg enjoyment and motivation) [31]. In medical and nursing education, AR has been shown to enhance knowledge and understanding [24,25], practical skills, and social skills [3]. This is important because, in the medical context, the busy schedules of teachers, practitioners, and students reduce available learning time, making additional improvements particularly beneficial. For instance, gamification in histology teaching can enhance student learning [32], and immersive games can support medical learning, especially in the context [33].

The effectiveness of immersive AR experiences in enhancing student understanding of complex physiological processes remains unclear, with a significant knowledge gap in the literature. The literature is inconclusive regarding what makes an AR-enhanced learning experience valuable and how to effectively convert academic content into AR [34-37]. This knowledge gap is compounded by a lack of rigorous studies, preventing meaningful meta-analysis [10]. Investigating when and how AR improves learning outcomes is essential. We investigate curiosity as a construct that could underpin a learning framework for AR-enhanced content [38]. The conversion of academic content into games or AR-enhanced content is little studied in the literature [39,40]. This conversion requires fundamental simplifications that may impact how students perceive and retain the content. It also determines what students take away from the AR-enhanced learning experience in the long term and requires more research to be evaluated [41].

This study explores the creation of AR-based content for immersive learning and its impact on student performance, focusing on enhancing a medical textbook through the AR Osteoclasts app. The aim of this project was to “augment” a medical cell atlas textbook with a strong focus on photorealistic imaging (Visual Biology in Oral Medicine, Quintessence Publishing [13]) by means of an AR-based App. The textbook itself was inspired by 6 computer-animated scientific videos on cell communication processes in a clinical context and state-of-the-art reviews [42-45]. We selected the process of bone resorption and new bone formation from the book chapter on osteoclasts as a basis for the app AR Osteoclasts was designed to provide an interactive visual experience of bone physiology, specifically bone resorption and formation, enabling users to engage with these processes in an AR environment (Figure 1 and Multimedia Appendix 1). To evaluate the app, we assessed the game on a technical level (Multimedia Appendix 1) and on learning outcomes with an experiment. To assess AR-enhanced content for learning, we formulated hypotheses on the general impact on learning (H1), specific improvements in learning (H2), and long-term retention (H3).

- H1: Learning motivation about osteoclasts would be improved with a serious AR game, compared with learning from text.
- H2a: Learning outcomes about osteoclasts are at least as effective with a serious AR game as learning from text.
- H2b: Learning outcomes about osteoclasts with a serious AR game improve on visual questions compared with learning from text.
- H2c: Learning outcomes about osteoclasts with a serious AR game are improved for curious learners compared with learning from text.
- H2d: Learning outcomes about osteoclasts with a serious AR game are improved for topics represented as visual components of the game.
- H3: Long-term learning outcomes about osteoclasts with a serious AR game are improved compared with learning from text.

## Methods

### Overview

To assess this AR serious gaming for learning in the context of Osteoclasts, we conducted an experiment comparing AR-based learning with traditional text-based learning. To test our hypotheses, we employed a difference-in-differences design for quantitative analysis of learning outcomes and utilized automated text analysis for qualitative assessment. The design of the AR game follows best practices [46,47] and focuses on converting scientific knowledge into gaming mechanisms involving stakeholders and experts [48]. The game design aims to enhance learning (Multimedia Appendix 1) through embodiment [49,50] in minigames [51]. Additionally, a usability study with current best practices [52-57] was conducted to assess the participants’ ability to effectively engage with the game (Multimedia Appendix 2). Last, we provide insights into participants’ experiences with a video of a single playthrough (Multimedia Appendix 3).

We conducted a power analysis to determine the necessary sample size for our study. We compared the effects of a serious AR game (AR-enhanced treatment) and text reading (text-only treatment) in a vignette experiment. The primary variable of interest was the difference in recall rate (correctly remembering an answer) between the two treatments. We aimed for an effect size of a 10% difference in the recall rate with a SD of 10% in recall rate. Using an α level of 0.05 and power of 0.8, the analysis indicated that a total of 32 participants (at least 16 per group) were required.

**Figure 2.**
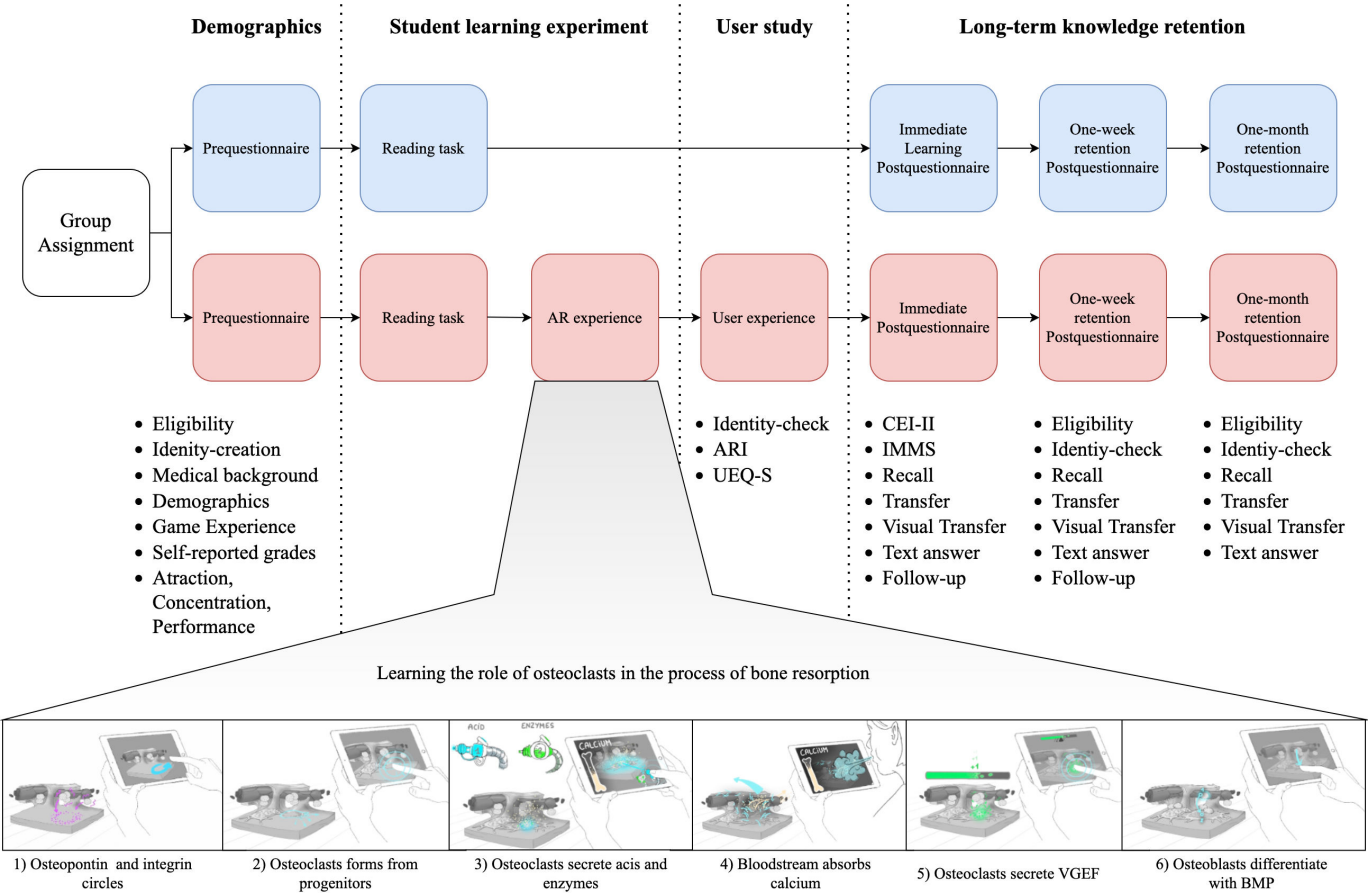
Protocol for the student learning experiment. Students were randomly assigned to the text-only group (blue) and the AR experience (red). The AR content (bottom images) included 6 short interactions covering osteoclast differentiation, lacuna formation, and osteoblast differentiation (Multimedia Appendix 1). Demographic information was collected before the experiment, and learning outcomes were measured at 3 points to assess long-term knowledge retention. ARI: augmented reality immersion; BMP: Bone Morphogenetic Protein; CEI-II: Curiosity and Exploratory Inventory-II; IMMS: Instructional Materials Motivation Survey; UEQ-S: User Experience Questionnaire – Short; VEGF: Vascular Endothelial Growth Factor.

Students were randomly assigned to either a text-only group or an AR-enhanced group (Figure 2). Both groups were presented with identical content, though through different learning methods. Both groups read a description of bone remodeling, while the AR-enhanced group followed up with an interactive AR game designed to provide an additional learning modality (Multimedia Appendix 1). Learning outcomes were assessed immediately after the treatment through a web-based survey, with follow-up assessments conducted at one week and one month to evaluate long-term retention.

### Hypothesis H1

First, we examined the relationship between individual learning experiences and motivation, specifically exploring how the game mechanisms influenced students’ motivation to learn (H1). To assess this, we used the Attention, Relevance, Confidence, and Satisfaction (ARCS) model of motivation—comprising Attention, Relevance, Confidence, and Satisfaction—which is measured through the Instructional Materials Motivation Survey [58,59]. Attention refers to how effectively the learning materials engage and capture the learner’s interest, while Relevance reflects the perceived connection between the materials and the real world. Confidence measures the learners’ belief in their potential for success in the discipline when using the materials, and Satisfaction assesses learners’ sense of accomplishment during the learning process. The full Instructional Materials Motivation Survey questionnaire [40] is included in Multimedia Appendix 4. To evaluate H1 and understand how participants perceive learning in AR, we analyzed the differences in ARCS model responses using a Wilcoxon signed rank test [58].

### Hypothesis H2a, H2b, and H2c

To assess hypothesis H2a, we assessed students’ learning outcomes using 3 distinct learning constructs and their composite. The questions are available in Multimedia Appendix 4. First, we included questions that measured recall, which captures the simplest form of knowledge retention. Second, we incorporated questions that assessed the transfer of knowledge, asking students to apply what they had learned in the game to new problems or contexts [60]. Last, we introduced a visual knowledge transfer task, where students were presented with visualizations of processes and asked to transfer knowledge from their learning experience to these new representations. This was particularly important as transferring knowledge to different forms of representation requires both an understanding of the content and its corresponding representation—a difficult task known as the “representation dilemma” [61]. To assess hypothesis H2b, we evaluated whether the game enhanced performance on visual transfer tasks, using visualizations that were not part of the game but depicted the same concepts.

To evaluate hypothesis H2c, our serious game was designed to leverage students’ curiosity and exploration affinity. As opposed to linear expository texts, curiosity may be especially impactful for learning outcomes in a gaming context. To account for this, we employed the Curiosity and Exploratory Inventory-II (CEI-II) [62], specifically focusing on students’ general curiosity traits. This approach allowed us to examine how students’ curiosity traits impacted their experience, as opposed to how the experience impacted students’ curiosity at the time of playing.

To assess H2a, H2b, and H2c, we developed linear regression models for each of the 3 learning constructs: Recall, Transfer, and Visual Transfer. Additionally, we constructed a composite model that combined the performance across all 3 constructs. In each model, the learning construct served as the dependent variable, with the following independent variables:


$$y=\beta_{0}+\beta_{1}\cdot Treatment+\beta_{2}\cdot CEI2+\beta_{3}\cdot ARI+\beta_{4}\cdot Grade+\;\;\;\;\;\;\;\;\;\;\;\;\beta_{5}\cdot Age+\beta_{6}\cdot Gender+\beta_{7}\cdot Experience+\beta_{8}\cdot Image+\;\;\;\;\;\;\;\;\;\;\;\;\;\;\;\;\beta_{9}\cdot Interaction+\beta_{10}\cdot Text+\beta_{11}\cdot Treatment\cdot CEI2+\varepsilon$$

The Treatment variable distinguishes between AR-enhanced and Text-only learning. The CEI2 variable represents the sum across all CEI-II items. The ARI (augmented reality immersion) variable represents the sum across all ARI items. The Grade variable is the student’s self-reported Swiss Grade, ranging from 1 (worst) to 6 (best), with 4 representing a passing grade. The Age variable is self-reported within a 5-year age bracket converted to a continuous scale. The gender variable is either male, female, or other; however, no participants in our sample identified as other. The Experience variable refers to participants’ prior knowledge of osteoclasts, rated on a 5-point scale from “no knowledge” to “regular interaction”. The Image, Interaction, and Text variables referred to preferred modes of learning as self-reported by the student, the sum of three 5-point scales for each. The coefficient $\beta_{11}$ was for the interaction between the treatment group and the CEI-II items sum.

Given the challenges of interpreting interaction models [63], we used marginal effects to better understand how the interaction between treatment and CEI-II impacts learning outcomes. To compute these effects, we used unit-level conditional estimates of the empirical distribution across the full range of the CEI-II scale (from 10 to 50 points), applying heteroskedasticity-robust SEs at a 90% CI [64]. Additionally, we conducted a Simple Slope Analysis [65] to investigate whether curiosity plays a different role in learning outcomes depending on the treatment condition.

### Hypothesis H2d

To assess hypothesis H2d, we examined which topics became particularly salient for students under the different treatment conditions. Students were asked 3 text-based questions related to bone remodeling (Table S2 in Multimedia Appendix 5 and survey questions in Multimedia Appendix 4). To encourage more detailed responses, we used encouragement designs [66], prompting students to provide longer and more detailed answers. The text answers provided by students were analyzed with structural topic modeling [67] to explore the relationship between student responses and the treatment conditions. Topic modeling identifies coherent themes within large sets of written documents, while structural topic modeling extends this method by incorporating covariates that include meta-information not present in the documents themselves. In our analysis, we include the treatment condition, the specific question answered, the individual student providing the response, and the time in weeks since the treatment.

Topic modeling is an iterative statistical process that is highly sensitive to initialization [67] and requires fine-tuning to produce reliable results. Therefore, we adhered to established best practices in topic modeling. The number of topics was a priori unknown and needed to be determined. Since the optimal number of topics was unknown a priori, we tested a range of topic counts from 2 to 20. We also examined the impact of including covariates by running models both with and without them (Section S9.1 in Multimedia Appendix 5). Based on these tests, we determined that 10 topics (K=10) were the most suitable for the text data we analyzed. To address potential initialization issues, we ran 100 models with 10 topics (K=10) and selected the best-performing model, balancing exclusivity and coherence [67].

Finally, the topics generated by the model required clear and meaningful labels for interpretation. To ensure high-quality labeling, 3 independent experts assigned labels to the topics, and we calculated the intercoder reliability index, Krippendorff α [68], to assess the consistency and accuracy of the labels. Krippendorff α ranges from –1 to 1, with a score of 1 indicating perfect agreement, 0 representing random labeling, and –1 signifying completely inverse labeling [69]. Generally, an α value greater than 0.80 indicates high reliability, while values above 0.67 are considered acceptable for the purposes of our analysis [68].

### Hypothesis H3

To assess hypothesis H3 on knowledge retention, participants completed knowledge assessment questionnaires at 3 time points: immediately following the learning experience, one week later, and one month later. However, due to participant attrition, the hypothesis could not be tested statistically. A multi-level model was initially planned to account for the repeated measurements but was not implemented due to the reduced sample size.

### Ethical Considerations

Our participants were selected from dental students in the final year of dental school, attending a course at the end of their studies with appropriate knowledge of the topic and content level. All students were informed in advance about the study taking place, and participation was optional. Students gave informed consent before participating. The data was handled confidentially in line with privacy regulations. Participants were not compensated for their participation. The study was approved by the ethics commission of the Canton of Zürich (Req-2022‐00290).

## Results

### Overview

No data points from the 39 student participants were excluded by the principal (23 female, 14 male, 2 NA). However, 2 participants dropped out during the experiment, resulting in attrition. Students were aged 27 years (SD 3.6; measures in 5 y blocks) on average. Most had heard of osteoclasts (36 students), and a few had worked with osteoclasts before (2 students). Six students started studying dental medicine with the Master program (0‐1 y in dental medicine), most students started with dental medicine in their Bachelor program (3 y of experience), and 6 students have extended experience (5 y of experience). Students also self-reported their CEI-II as 32.26 on average (SD 6.74).

For a long-term assessment, we asked the participants to recall the learned material after one week and after one month. Participation in the follow-up survey was voluntary. The first follow-up survey after a week had 6 responses, and the second follow-up survey after a month had 15 responses. Due to limited response rates, statistical analysis of the follow-up data was not feasible according to our power analysis.

### Hypothesis H1

**Figure 3.**
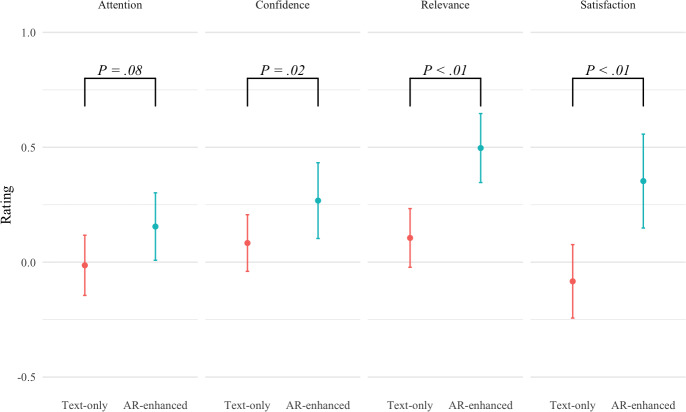
Evaluating the Instructional Materials Motivation Survey responses based on the ARCS construct with 95% CIs, we found no significant difference in Attention (W=18,619; *P*=.08) between the learning scenarios. However, participants reported increased Confidence (W=11,882.5; *P*=.02) , greater perceived Relevance (W=10,417; *P*<.001) of the material, and higher Satisfaction (W=4561; *P*<.001) with the learning experience. AR: augmented reality; ARCS: Attention, Relevance, Confidence, and Satisfaction.

We examined the participants’ subjective learning experience using the ARCS model (Figure 3). We found significant effects for Confidence (W=11,882.5; *P*=.02), Relevance (W=10,417; *P*<.001), and Satisfaction (W=4561; *P*<.001). Although there was no difference in performance, participants reported higher motivation overall—feeling more confident, perceiving the topic as more relevant, and expressing greater satisfaction with their progress. Interestingly, we did not find significant differences in Attention (W=18,619; *P*=.08), which contrasts with previous findings that interactive learning tools tend to enhance attention [70,71].

### Hypothesis H2a

We reported the participant responses as the proportions of correct answers for each question. For recall tasks (Figure S20 in Multimedia Appendix 5), we reported very high correct responses, indicating a ceiling effect. Several questions were answered correctly by 100% of the participants, although there were minor differences between treatment groups, which appeared to be random. For transfer tasks (Figure S21 in Multimedia Appendix 5), we observed that 2 questions appeared simple, with very high correct response rates, similar to recall tasks. The other 3 questions had high correct response rates, ranging from 50% to 70%. For visual transfer tasks (Figure S22 in Multimedia Appendix 5), we reported the lowest correct response rates. A Welch *t* test revealed no significant difference between the AR and text treatments in learning outcomes (*t*=2.0103; *P*_Bonferroni_=.095). Although text treatments had nominally higher scores across all transfer tasks, the differences were not statistically significant.

### Hypothesis H2b

In the regression analysis (Table 1), we reported no significant results for recall and transfer learning. These models had adjusted *R*^2^ of 0.03 and 0.12, respectively, and their *F* statistics were not significant. However, the models for Visual Transfer and Total cumulative score showed a significant *F* statistic (*F*_11,24,visual transfer_=2.72, *P*=.02; *F*_11,24,total_=2.81, *P*=.02). Only the visual transfer model revealed significant effects for the AR treatment (*t*=−2.099; *P*_visual_transfer_= .046). All other models could not discern a difference between the treatment conditions for the outcome variables. Across models, we also found that previous experience with osteoclasts was a strong predictor of the result (*t*_total_=2.469, *P*_total_=.02; *t*_transfer_=1.865, *P*_transfer_=.07; *t*_visual transfer_= 1.789, *P*_visual transfer_=.09). For the visual transfer task, we also find a significant effect of the CEI-II (*t*_visual transfer_ =−2.518, *P*_visual transfer_=.02), the interaction effect (*t*_visual_transfer_=1.913, *P*_visual transfer_=.06) and the preference for text learning (*t*_visual_transfer_=2.575, *P*_visual_transfer_=.02). Last, the cumulative score also showed a significant effect for the participants being male (*P*_total_=.06) which may be attributed to motivation [72] but cannot be discerned by this study.

**Table 1.** Student outcomes for the 3 learning tasks and a cumulative score.

	Dependent variable			
	Total	Recall	Transfer	Visual transfer
AR^a^ treatment				
Effect size (SE)	–0.211 (0.172)	0.071 (0.205)	–0.106 (0.358)	–0.615 (0.293)
*t* value (*df*=11)	−1.261	0.347	−0.296	−2.099
*P* value	.22	.73	.76	.05
CEI-II^b^				
Effect size (SE)	–0.001 (0.003)	0.005 (0.004)	0.005 (0.007)	–0.014 (0.006)
*t* value (*df*=11)	−0.401	1.196	0.798	−2.518
*P* value	.69	.24	.43	.02
ARI^c^				
Effect size (SE)	0.003 (0.002)	0.005 (0.003)	0.007 (0.003)	–0.002 (0.003)
*t* value (*df*=11)	1.647	1.737	1.842	−0.569
*P* value	.11	0.095	.07	.57
Swiss grade				
Effect size (SE)	0.044 (0.047)	0.049 (0.056)	0.071 (0.097)	0.008 (0.079)
*t* value (*df*=11)	0.956	0.979	0.727	0.107
*P* value	.34	.33	.47	.91
Age (years)				
Effect size (SE)	–0.006 (0.005)	0.004 (0.006)	–0.011 (0.011)	–0.012 (0.009)
*t* value (*df*=11)	−1.160	0.693	−0.972	−1.338
*P* value	.25	.49	.34	.19
Male				
Effect size (SE)	0.085 (0.041)	0.084 (0.049)	0.083 (0.085)	0.082 (0.069)
*t* value (*df*=11)	2.081	1.834	0.978	1.182
*P* value	.05	.08	.33	.24
Experience				
Effect size (SE)	0.195 (0.079)	0.037 (0.096)	0.307 (0.165)	0.241 (0.134)
*t* value (*df*=11)	2.469	0.392	1.865	1.789
*P* value	.02	.69	.07	.09
Image preference				
Effect size (SE)	–0.01 (0.014)	0.002 (0.017)	–0.034 (0.029)	0.002 (0.023)
*t* value (*df*=11)	−0.708	0.130	−1.177	0.103
*P* value	.49	.9	.25	.92
Interaction preference				
Effect size (SE)	–0.004 (0.015)	–0.013 (0.018)	0.008 (0.030)	–0.008 (0.025)
*t* value (*df*=11)	−0.305	−0.740	0.267	−0.346
*P* value	.76	.46	.79	.73
Text preference				
Effect size (SE)	0.016 (0.012)	–0.019 (0.015)	0.013 (0.026)	0.054 (0.021)
*t* value (*df*=11)	1.293	−1.329	0.516	2.575
*P* value	.21	.2	.61	.02
Interaction of AR treatment and CEI-II				
Effect size (SE)	0.004 (0.005)	–0.005 (0.006)	–0.001 (0.011)	0.017 (0.009)
*t* value (*df*=11)	0.772	−0.710	−0.119	1.913
*P* value	.47	.48	.91	.07
Constant				
Effect size (SE)	0.156 (0.432)	0.537 (0.515)	–0.244 (0.900)	0.175 (0.736)
*t* value (*df*=11)	0.362	1.043	−0.271	0.238
*P* value	.72	.31	.79	.81
Observations	36	36	36	36
*R* ^2^	0.563	0.354	0.411	0.555
Adjusted *R*^2^	0.362	0.058	0.142	0.351
Residual SE (*df*=25)	0.100	0.117	0.204	0.167
*F* test (*df*=11, 24)	2.807	1.196	1.526	2.719
*P* value	.02	.34	.19	.02

a AR: augmented reality.

b CEI-II: Curiosity and Exploratory Inventory-II.

c ARI: augmented reality immersion.

### Hypothesis H2c

**Figure 4.**
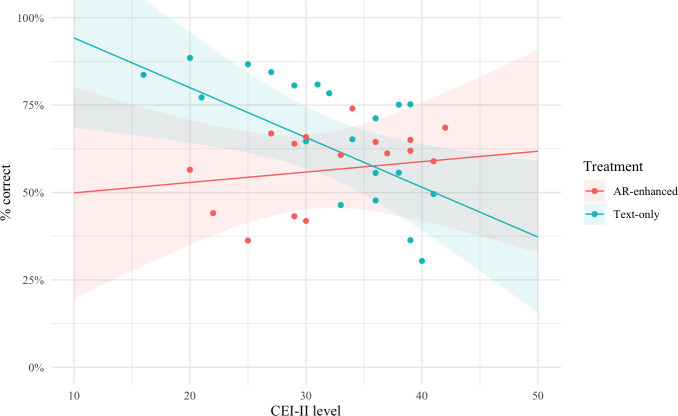
A simple slope analysis of our model revealed a strong inverse relationship between reading and curiosity in the students (1.4% decrease in correct responses, *t*_24_=−2.518, *P*=.02). For students who were curious, this could be offset by experiencing the information in AR. AR: augmented reality; CEI-II: Curiosity and Exploratory Inventory-II.

We investigate the marginal effects of the model for visual transfer learning (Figure 4). In the range of the CEI-II index, we detect an inflection point at 36 points. For lower CEI-II scores, text-only learning yields the best results. However, for higher CEI-II scores, AR experiences help participants to score higher. The results are limited by the empirical distribution not having observations for CEI-II under 16 or over 42, making the behavior in the tails of the distribution speculative. We employ a Simple Slope Analysis and detect that the text-only treatment has a significant negative slope for CEI-II (1.4% decrease in correct responses, *t*=−2.518, *P*=.02), whereas the CEI-II has no significant effect under the AR-enhanced treatment (*t*=0.347, *P*=.73).

### Hypothesis H2d

We first analyzed the frequency of word occurrences in participants’ responses (Figure S24 in Multimedia Appendix 5). The words identified generally aligned with the topics addressed in each question. When categorizing responses, certain terms are clustered into thematic groups. For the formation of osteoclasts, the most common answers could be grouped into precursors (hemopoetic, progenitor, and stem) and receptors (RANKL [Receptor Activator of NF-κB Ligand], Arginylglycylaspartic acid, and bind). For the function of osteoclasts and osteoblasts, the answers are grouped according to the 2 cell types: osteoclasts (resorb and dissolve), and osteoblasts (form and build). The osteogenic differentiation is separated into cell-related words (bone cell, mesenchyme, osteoblast, osteoclast, and stem) and signaling/receptor-related words (Wingless und Int-1 [Integrated], Bone Morphogenetic Proteins, RANKL, and RANK [Receptor Activator of NF-κB]).

Our topic models were labeled by 3 independent raters, and the intercoder reliability was calculated using Krippendorff α, which reached a value of 0.717. This indicates good intercoder reliability. Although full consensus was not always reached, in cases of disagreement, we relied on the most probable words from the topic contrasts (Section S9.2 in Multimedia Appendix 5) to determine a label for the topics. We looked at the prevalence of topics across all texts, where 100% represents all words written (Figure S25 in Multimedia Appendix 5). The topics of osteogenic differentiation and RANKL signaling were the most frequently discussed, each accounting for approximately 15% or more of the total text. Topics related to differentiated cells, the remodeling site, and stem cells each made up more than 10% of the text. The topics of bone resorption mechanisms, RANK signaling, bone remodeling, and multinucleated cells each contributed more than 5% of the total text. The smallest topic was the “Don’t know” category, which captured uncertain or unclear responses from students.

**Figure 5.**
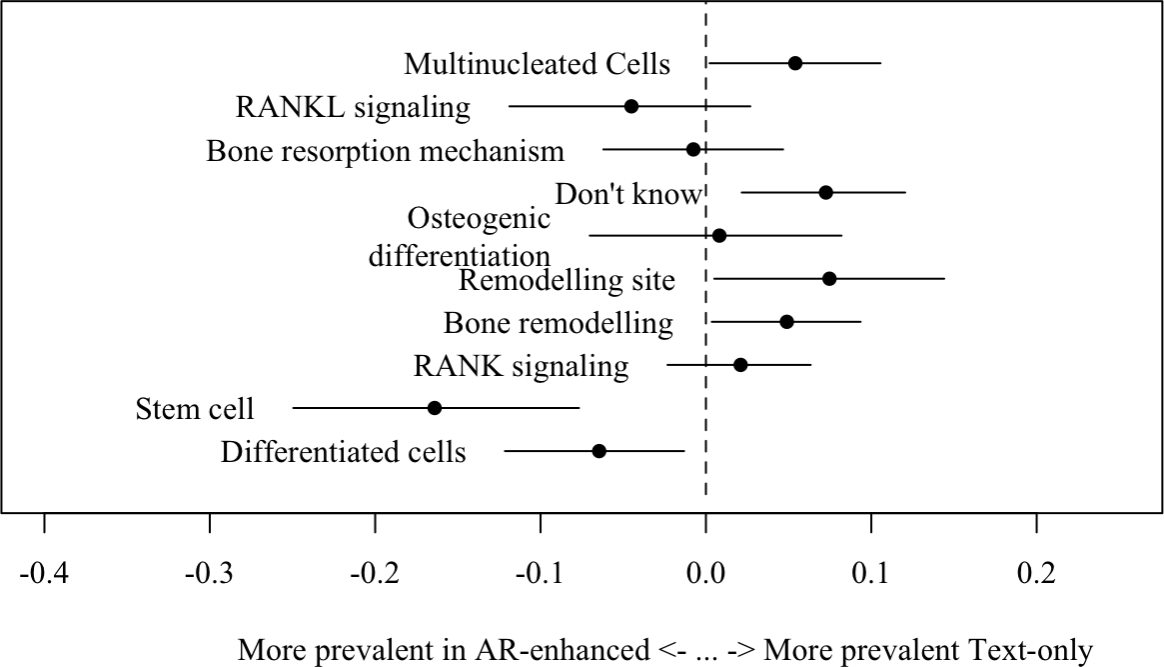
Topic prevalence by treatment. Negative values indicate an increased prevalence to appear in the AR-enhanced treatment, while positive values indicate an increased prevalence to appear in the text-only treatment. When the dashed line is crossed, no significant prevalence for either treatment is detected. AR: augmented reality; RANK: Receptor Activator of NF-κB; RANKL: Receptor Activator of NF-κB Ligand.

We used the properties of structural topic modeling to assess whether the treatment type influenced the topics students focused on (Figure 5). We found that in the AR-enhanced treatment, students wrote more frequently about the stem cells (*t*=−3.681, *P*<.01) and differentiated cells (*t*=−2.303, *P*=.02) In contrast, students in the text-only treatment tended to focus more on the remodeling site (*t*=2.220, *P*=.03) and the process of bone remodeling (*t*=2.108, *P*=.03). People in the text-only treatment were more likely to respond with “Don’t know” (*t*=2.953, *P*<.01). No significant differences were observed for other topics between the two treatment groups.

Topics may also be influenced by the 3 questions we asked (Figure S37 in Multimedia Appendix 5). To determine whether a topic was associated with a particular question, we examined the topic across all 3 paired comparisons between questions. For instance, the topic on multinucleated cells was associated less frequently with the question on osteoclast formation (Q1) but was strongly associated with both the coordinated function of osteoclasts and osteoblast in the canopy (Q2) and osteogenic differentiation (Q3). That indicated that participants were statistically significantly more likely to talk about multinucleated cells in Q2 and Q3.

In the formation question (Q1), participants were less likely to talk about RANK-RANKL signaling and stem cells and multinucleated cells, and more likely to talk about bone remodeling, bone resorption, the remodeling site, differentiated cells, stem cells, or osteogenic differentiation. In the functions question (Q2), participants were less likely to talk about bone remodeling, bone resorption mechanism, the remodeling site, or differentiated cells, and more likely to talk about osteogenic differentiation, RANK-RANKL signaling, and stem cells and multinucleated cells. In the differentiation question (Q3), participants were less likely to talk about bone resorption mechanism, stem cells, RANK signaling, or osteogenic differentiation, and more likely to talk about bone resorption, the remodeling site, differentiated cells, multinucleated cells, and RANKL signaling.

### Hypothesis H3

Due to participant attrition, we are unable to report statistical results for the long-term study. However, those participants who did complete the follow-up showed remarkable performance when playing the AR game (Figure S23 in Multimedia Appendix 5). In some cases, participants even showed signs of improved retention over time. However, due to the small sample size, further analysis of these trends was not possible.

## Discussion

### Principal Findings

In this study, we evaluated the use of an AR medical teaching game in a classroom setting. Before examining the effectiveness of our difference-in-differences design comparing text-based and AR-based learning, we briefly summarize the usability study (MultimediaAppendices 2  6), which showed that participants adapted well to the AR experience with minimal difficulty. In our vignette experiment, one group received the AR game with a written text (AR-enhanced), while the second group only received the text (text-only). The goal was to assess whether the AR game enhances student engagement, motivation, and learning outcomes, as well as long-term knowledge retention. Our findings suggest that immersive media positively impacts motivation (H1). The learning experiment showed similar learning outcomes across treatments (H2a), while visual learning was not significantly improved overall (H2b), likely due to ceiling effects from easier questions, though it did improve for curious students (H2c). Topic modeling revealed that AR learners focused more on cells in line with the game design, while text learners focused more on processes (H2d). Due to participant attrition, we could not assess long-term learning (H3). In summary, AR facilitated the learning of key concepts, with curious students benefiting the most.

### Principal Results

We found that presenting advanced scientific content through AR significantly improves motivation (H1) by enhancing students’ subjective learning experience. AR increased engagement with the process of bone remodeling, helping students develop a deeper appreciation for the topic and perceive it as more important, according to the ARCS model. Students who interacted with the AR scene attributed higher value and relevance to the concepts, forming a stronger personal connection to the content by recognizing its value. Additionally, the playful nature of the AR experience led students to perceive the material as easier to learn, which, despite not improving assessment performance, increases overall satisfaction and may reduce learning pressure.

We also aimed to examine the impact of AR-enhanced content on students’ learning outcomes. We started with a conservative hypothesis that students in the AR experience would achieve comparable learning outcomes (H2a). While we anticipated improved performance on visual tasks due to the visual nature of the game (H2b), this expectation was not confirmed. Instead, we found that only highly curious students performed better on visual transfer tasks when learning with AR-enhanced content compared with a text-only approach (H2c). Specifically, a negative relationship between text-only learning and curiosity was observed, which was mitigated by the AR game for visual transfer tasks. Students with higher curiosity performed better in the AR condition than in the text-only condition. We identified a cutoff at CEI-II of 35 beyond which students improved performance on visual transfer tasks following the AR experience. Since dental medicine involves interpreting visual tasks, AR representations may offer a valuable tool to enhance student performance, particularly for curious learners.

We expected that the visual narrative of the AR game would direct students’ attention to specific concepts. Indeed, the game’s emphasis on cellular mechanisms influenced the text responses of participants and shaped how they learned and retained the material (H2d). Notably, the students in the AR-enhanced treatment group mentioned the cells more compared with the text-only treatments, whose responses focused more on processes and locations. The interactive elements and game mechanics of the AR experience appeared to guide students’ language and understanding when answering questions. The choice of how the scientific process was translated into game mechanics played a crucial role in shaping students’ learning experiences. For most AR games, the process of translating knowledge into game mechanics is only focused on playability, but our investigation highlights the importance of this aspect, as detailed in the game design and usability study (MultimediaAppendices 1  2).

Although our sample size was insufficient for a statistical analysis of long-term knowledge retention (H3), we did observe some significant trends in the text responses (Section S9.4 in Multimedia Appendix 5). Specifically, we found that general topics increased in prevalence, while more specific topics, such as RANK-RANKL signaling, decreased. Most topics maintained their prevalence. This suggests that a stable core of learned concepts persisted over time, though with a slight loss of detail.

### Limitations

We identified 4 major limitations of this study that can guide future research on serious gaming in AR for educational purposes. Future investigations should focus on (1) understanding the role of student/player characteristics, (2) effectively translating scientific knowledge into game mechanics, (3) evaluating the effectiveness of long-term assessments, and (4) ensuring that the tests used to measure learning outcomes are appropriate for assessing the game’s impact.

First, regarding player characteristics (Multimedia Appendix 2), it is important to recognize that games are played by individuals with diverse backgrounds and gaming skills. Casual gamers [73], who are often less familiar with AR experiences, differ from participants who prefer speedruns or completionist approaches [74] (Figure S41 in Multimedia Appendix 2 ). Content designed for more advanced players may be less accessible or engaging for casual gamers. To ensure that a serious game effectively imparts knowledge, game design must consider varying play styles. Our observations revealed that most participants were casual gamers, suggesting that performance issues in the average playtime group were more related to user interface and game dynamics than to the learning content itself [75,76].

Second, when translating a scientific process into a game, decisions must be made about what to represent, what to omit, and which aspects of the process to emphasize (visually, interactively, and mechanically) versus those that are only indirectly conveyed [77]. Our findings showed that AR participants focused more on cells, while text-only participants concentrated on location and process. This suggests that AR encouraged students to engage more deeply with the game’s core mechanisms, that is, the cells, while somewhat distancing them from other aspects, such as the overall process. Given the strong visual guidance in the AR game, it is crucial that the game not only presents knowledge but also highlights key concepts as part of the immersive experience. To improve AR-enhanced learning, it would be beneficial to follow experimental design frameworks like design, experiment, analyze, and reproduce [78] to ensure that both content delivery and learning outcomes are aligned in a reproducible framework [79].

Third, the small number of participants due to the class size and the participant attrition for the longitudinal study limited our ability to draw conclusions. While some promising trends were observed in the long-term study—such as certain students showing improved knowledge retention after exposure to the AR treatment—it is unclear whether these effects were driven by individual differences or the treatment itself. The interaction between curiosity and the AR treatment is also intriguing but requires further investigation with a larger participant group to provide robust evidence of a positive relationship between curiosity and gamified educational content.

Fourth, another limitation was the selection of tests used to assess learning outcomes. The questions designed for recall, transfer, and visual transfer tasks must not only align with the content but also be sufficiently challenging to differentiate responses across treatments. In this study, the questions may have been too easy, leading to a ceiling effect that prevented us from detecting significant differences between participants at the expected effect sizes. Most participants scored 75% or higher (at least 8 out of 11 questions) on recall and transfer tasks, with previous knowledge of osteoclasts being the strongest predictor of performance. As a result, the impact of AR on learning outcomes may have been more evident in the visual transfer task, where participants showed more variation.

### Comparison With Prior Work

Research on the use of serious gaming for medical education reveals mixed findings regarding the efficacy of AR games, though there is growing evidence of their positive impact on student engagement and learning experiences. We identified 21 studies on serious gaming in medical learning, providing valuable insights that complement our findings. Despite the mixed outcomes in the literature, there is a gradual increase in the adoption of game-based learning approaches in education [80]. Students generally report higher content acceptance and more positive learning experiences when engaging with serious games [81,82]. Furthermore, process learning through serious games, particularly in flipped classroom settings, has been shown to prepare students effectively for tasks [83]. Overall, gamified teaching formats are widely well-received by learners and offer the potential to create immersive educational experiences.

The positive effects of intrinsic benefits are contrasted with the question of why some studies have null-finding and others do not. Null findings are reported regularly in both individual studies [34] and meta-reviews (13 studies [35], 27 studies [36], and 7 studies [37]). These attest to no significant benefits in the use of virtual reality (VR) or AR for learning outcomes. However, they equally suggested that VR and AR could promote intrinsic benefits such as increasing learner immersion and engagement. Our findings replicate these positive effects, while also suggesting a potential moderator that may explain when studies yield null findings versus positive results. Specifically, we identify curiosity to engage the gaming content as a key factor that drives visual understanding of content, and therefore, benefits can only be detected when tasks and assessments capture this criterion.

Studies showing positive outcomes often focus on the enhancement of spatial abilities in medical students, who benefit from the embodied experience offered by VR/AR [84-88]. A study in 2021 compared the teaching efficiency of VR with traditional education [89] and found that VR students had a significantly higher pass rate than those receiving conventional medical training. The VR students also demonstrated a better understanding of various processes and procedures. Another study [90] examined whether AR could improve short-term anatomical knowledge in head and neck anatomy compared with traditional 2D screen-based learning, with the AR group outperforming the traditional learning group. A further study [36] highlighted that VR/AR-based teaching not only improved medical education but also positively influenced student enthusiasm and enjoyment. In the field of occupational medicine, immersive VR serious games help students relate to patients’ states and circumstances [33]. In our study, we observed that AR-based learning positively impacted visual transfer tasks, particularly for curious students. This may have been due to eliciting enhanced spatial understanding fostered by the AR experience in the medical context.

### Conclusions

AR-enhanced learning holds great promise as an educational tool for the 21st century, particularly in the medical sciences, but also beyond. Immersive serious gaming in AR offers a compelling opportunity to engage students with complex scientific content, which can often be difficult to communicate and may deter new learners. However, the most critical factor that shapes student learning from AR-enhancement is the conversion of scientific knowledge to game mechanics. Additionally, AR has the potential to promote equity in the classroom by offering more inclusive learning opportunities. Our findings reinforce previous research showing that AR fosters a stronger connection with content, resulting in more positive perceptions of learning. While no significant learning benefits were observed for simpler tasks, consistent with existing literature, AR proved especially beneficial for more complex visual transfer tasks. We identified curiosity as a key factor that moderates the effectiveness of AR learning, with curious students experiencing greater learning gains. Ultimately, immersive serious gaming provides students with new perspectives on the content. Our findings indicated that particularly curious students benefited from interactive learning and could improve their learning outcomes compared with a text-only learning environment.

## Supplementary material

10.2196/64751 Multimedia Appendix 1 Game design and scientific background for the serious game.

10.2196/64751 Multimedia Appendix 2 Usability study of the serious game.

10.2196/64751 Multimedia Appendix 3 Outlines of the survey questions fielded in Qualtrics.

10.2196/64751 Multimedia Appendix 4 Zip file that contains survey data, text response data, and game data used in the analysis.

10.2196/64751 Multimedia Appendix 5 Advanced statistical analysis of the data not included in the main text.

10.2196/64751 Multimedia Appendix 6 Video of playing AR Osteoclasts from beginning to end.

## Abbreviations

AR: augmented reality
ARCS: Attention, Relevance, Confidence, and Satisfaction
ARI: augmented reality immersion
CEI-II: Curiosity and Exploratory Inventory-II
RANK: Receptor Activator of NF-κB
RANKL: Receptor Activator of NF-κB Ligand
VR: virtual reality
