# Supplementary material for: Applying Augmented Reality to Convey Medical Knowledge on Osteoclasts to Users of a Serious Game: Vignette Experiment
Source: JMIR Serious Games. 2025 Jun 16;13:e64751. doi: 10.2196/64751 (PMC12185033; doi:10.2196/64751)
Supplement: Multimedia Appendix 1 — Game design and scientific background for the serious game. [file games-v13-e64751-s001.docx]

**Applying Augmented Reality to Convey Medical Knowledge on Osteoclasts to Users of a Serious Game: A Vignette Experiment**

## Multimedia Appendix 1: Game design and scientific background

### S1 Game design


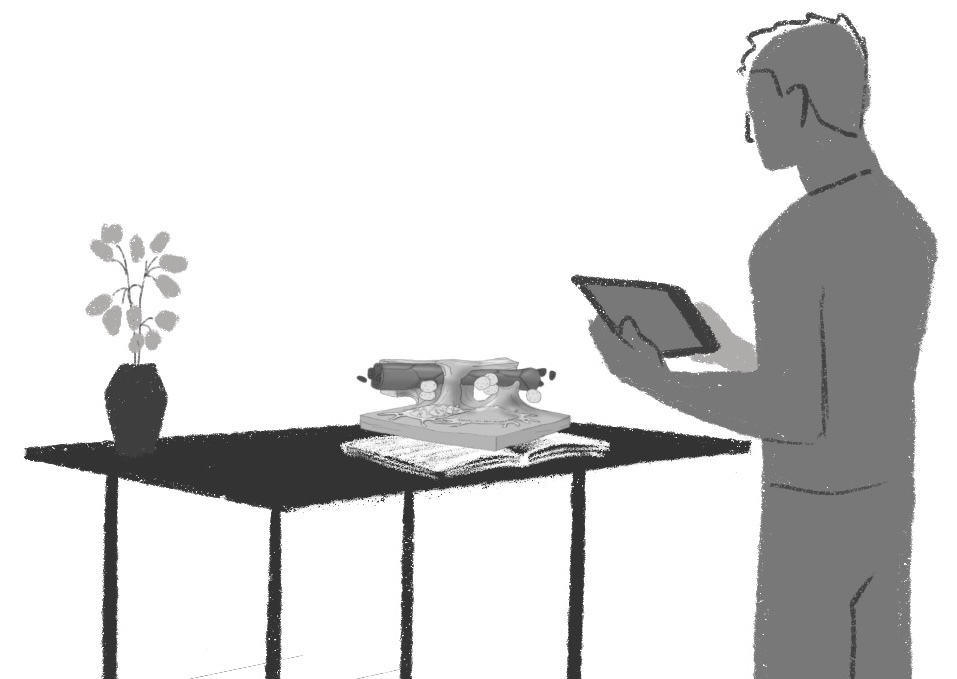


Figure S1: Scene overview. A user is aiming at the book *Cell-to-Cell Communication* [1] to view the scene of AR Osteoclasts and play the game.

The game employed Augmented Reality (AR) to experience the process of bone resorption through embodiment [2,3]. Figure S1 illustrates the concept of AR: the user is holding a tablet and aiming the camera at the book *Cell-to-Cell Communication* [1]. An image in the book triggers a virtual scene, shown on the device’s screen, seemingly on top of the book. The game has to balance scientific accuracy with the gaming experience and we employed an expert-reviewed, stakeholder-centered approach [4]. A series of three minigames were presented to the learner to guide them through the story. Minigames are an effective tool to communicate knowledge because they present a single concept in a playful manner [5]. We developed a game flow that mapped scientific concepts to minigames. The game was also meant to be a scientific tool of inquiry [6,7] to understand learning behavior [3].

#### S1.1 Gameflow

AR Osteoclasts takes place on the surface of a bone with a nearby blood vessel to provide the necessary backdrop for all processes involved in bone remodeling, see Figure S2. All Minigames also are located on the square bone surface and allow the user to experience several aspects of the microcosm of bone remodeling. Typically, minigames are fun short interactions that allow the users to experience and advance a story. In the first stage of this project, the process of bone remodeling as known to science was the starting point. We investigated possible game mechanisms for each step of the process (see Appendix 2D for the details of the process). Several options were drafted (see Appendix 2E for a map of potential processes), tested with paper prototypes [8] and a final selection was set for the three mini-games implemented in AR Osteoclasts, see Table S1.


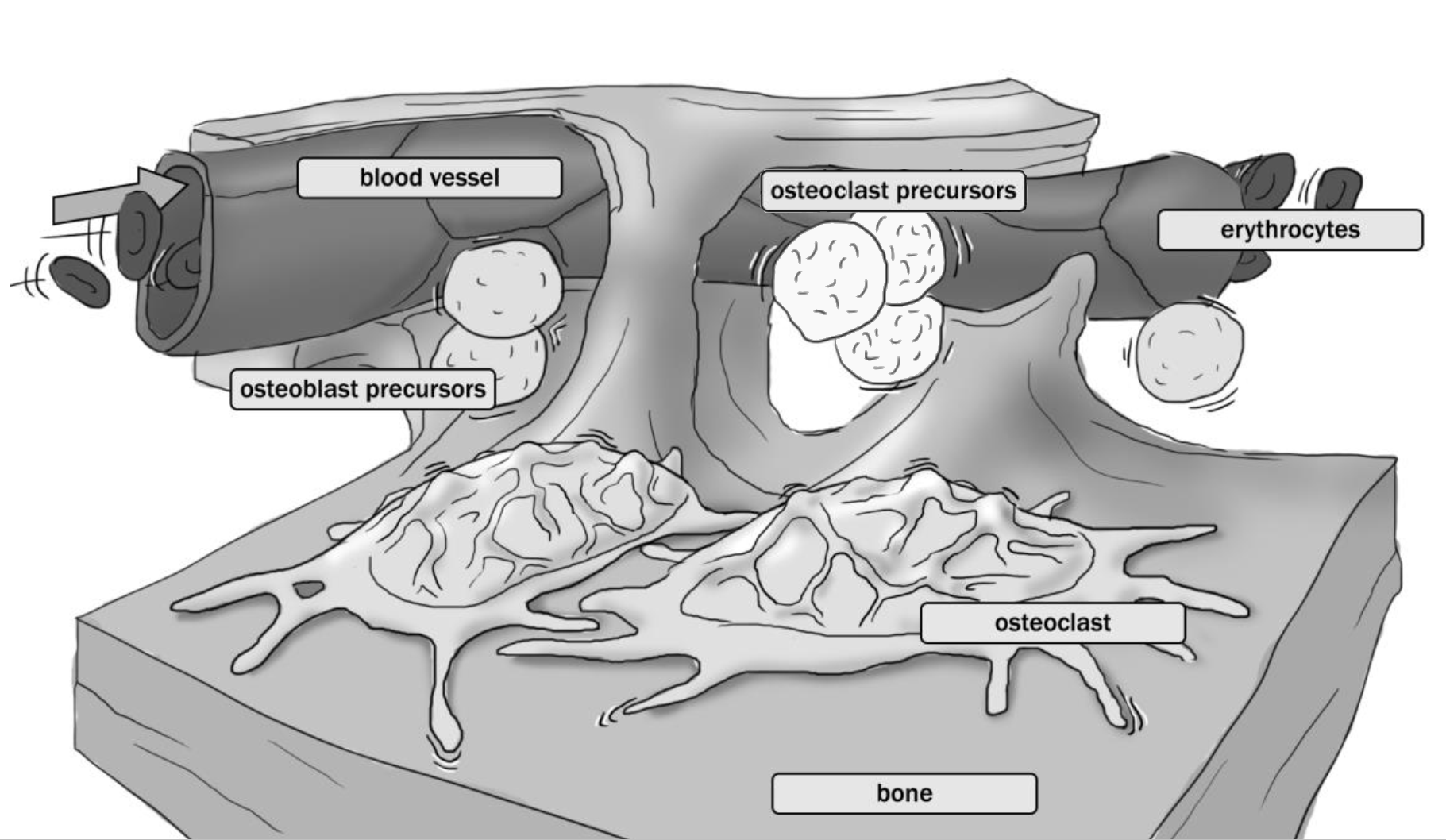


Figure S2: Game Setting. A blood vessel above the bone surface transports all cells and minerals. The Osteoclasts form on the surface of the bone.

Table S1. Overview of the translation of scientific knowledge into a serious gaming.

|  | Scientific  Background | Gameplay Implementation | User Interaction |
| --- | --- | --- | --- |
|  |  |  |  |
| Minigame 1: Osteoclast (Figure S6) | | | |
|  | Only cells in contact with bone can further differentiate to osteo- clasts. 10-20 osteoclast precursor cells fuse to form a mature osteoclast. Osteoclasts attach to osteopontin via a circle of integrins and form a resorption lacuna. Osteoclasts form a ruffled border. | Help the osteoclast precursor cells join together with and find the landing position. (1) Ostepontin on the bone surface allows the cells to attach to. (2) Several osteoclast precursor cells should be joined together with RANKL to get enough mass for an osteoclast. | (1) Draw a circle on the bone surface where the integrin and osteopotin connect to keep the osteoclast precursor cells in place.  (2) Swipe on the screen to push the osteoclast precursor cells towards the circle on the bone surface until they form a critical mass (10 cells). |
| Minigame 2: Lacuna (Figure S27) | | | |
|  | Osteoclasts secrete acid (pH4) and acid resistant enzymes. The acid demineralises the bone, enzymes degrade collagen. Calcium is secreted to the canopy and further to the bloodstream. | The surface of the osteoclast becomes transparent in an animation. (1) Remove calcium and collagen to form lacuna. (2) Get the calcium up the canopy to the blood vessel. | (1) Aim AR gun (acid or enzyme) on calcium or collagen to remove it. (2) Clear out the laguna by blowing on the screen (microphone detects sound). A bar indicates when the game is completed. |
| Minigame 3 Osteoblast (Figure S28) | | | |
|  | Osteoclast activity attracts osteoblast precursor cells. Osteoblast precursor cells clean the resorption lacuna with membrane bound serine proteinases. (BMP), which stands out of the bone surface is made soluble. BMP by chemotaxis attracts more osteoblast precursor cells and differentiates them to osteoblasts. | (1) The osteoclasts call in the osteoblast precursor cells for the next step. The osteoclasts performs apoptosis (cell suicide) to clear the way for the osteoblast precursor cells. Osteoblast precursor cells clean the lacunae, receive BMP from the bone and turn into osteoblasts proper. | (1) Tap quickly on osteoclast to release VEGF. The cell disappears after 10 taps. Each tap calls osteoblast precursor cells from the bloodstream. (2) They can be swiped towards the lacune. (3) To transform them into osteoblast, the BMP in the bone is activated by tapping on the osteoblast precursor cells 3 times. |

The mini-games centred around three main objects in the process of bone remodeling, the arrival of the osteoclasts (see Figure S6), the function of the resorption lacuna (see Figure S7), and the arrival of the osteoblasts (see Figure S8). The interactions were optimized through several rounds of playtests to ensure they were easy to perform [9]. Several interactions were simplified to improve usability compared to scientific accuracy.

a)
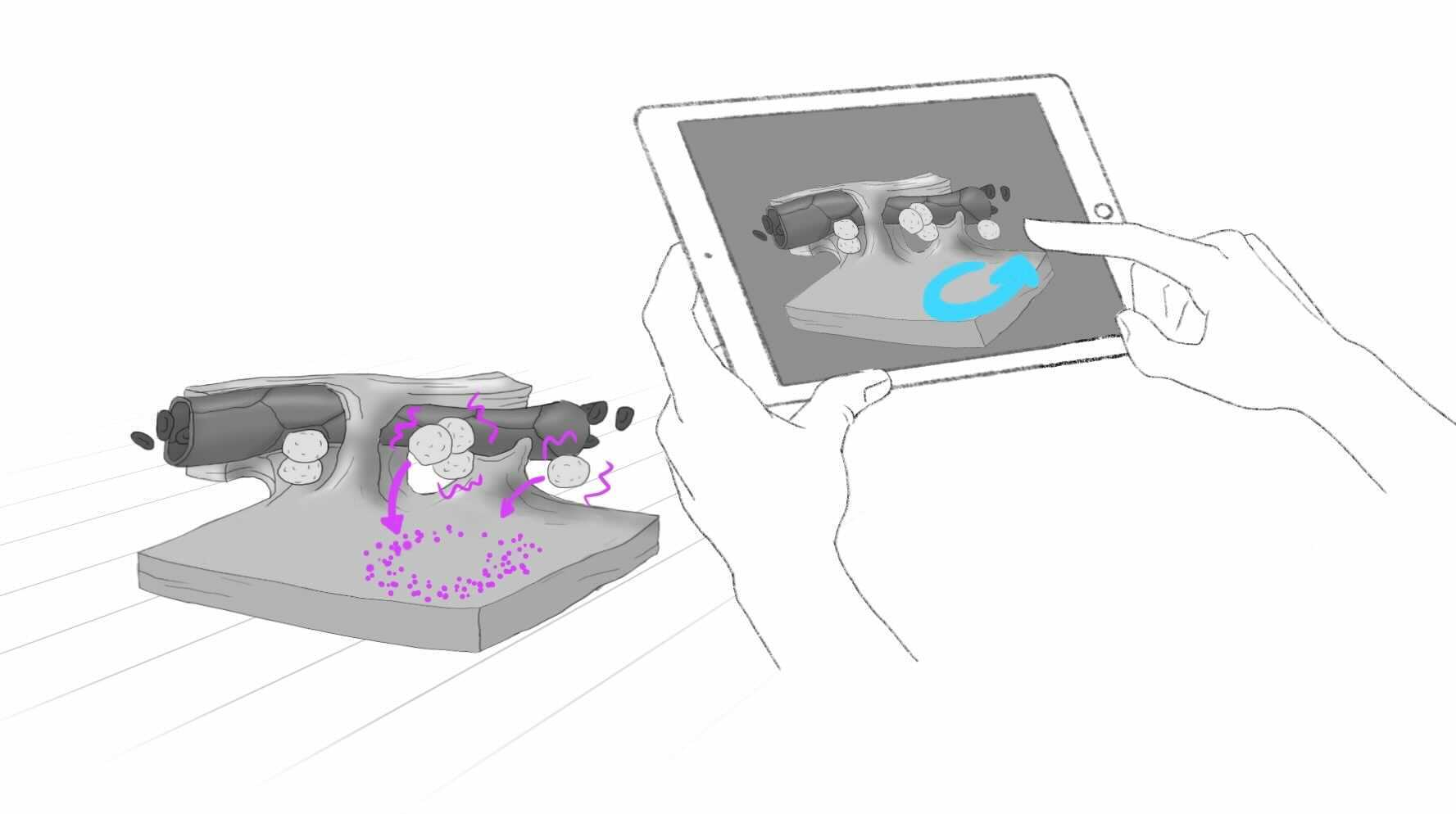
b)
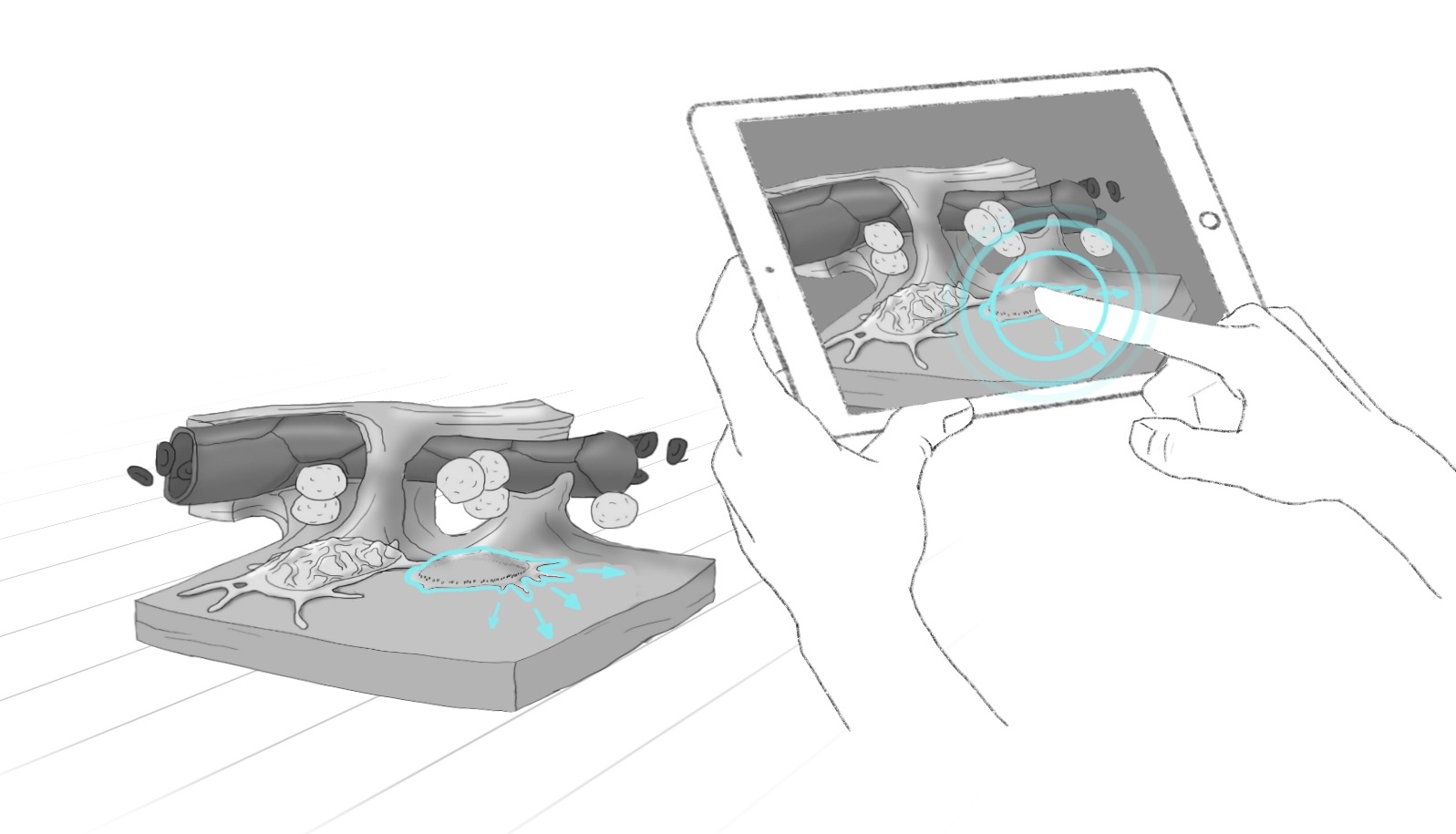


Figure S3: The first mini-game covered the arrival of osteoclasts. This minigame corresponded to storyboard 1-07, 1-08, 2-09, 2-10 (see Appendix 2D). In this minigame, the osteoclast precursor cell aggregates were attached to the bone to form an osteoclast and start the bone resorption process. a) Osteoclast precursor cells landed where osteopontins were. b) The attachment of the osteoclast precursor cells and the formation of osteoclast.

a)
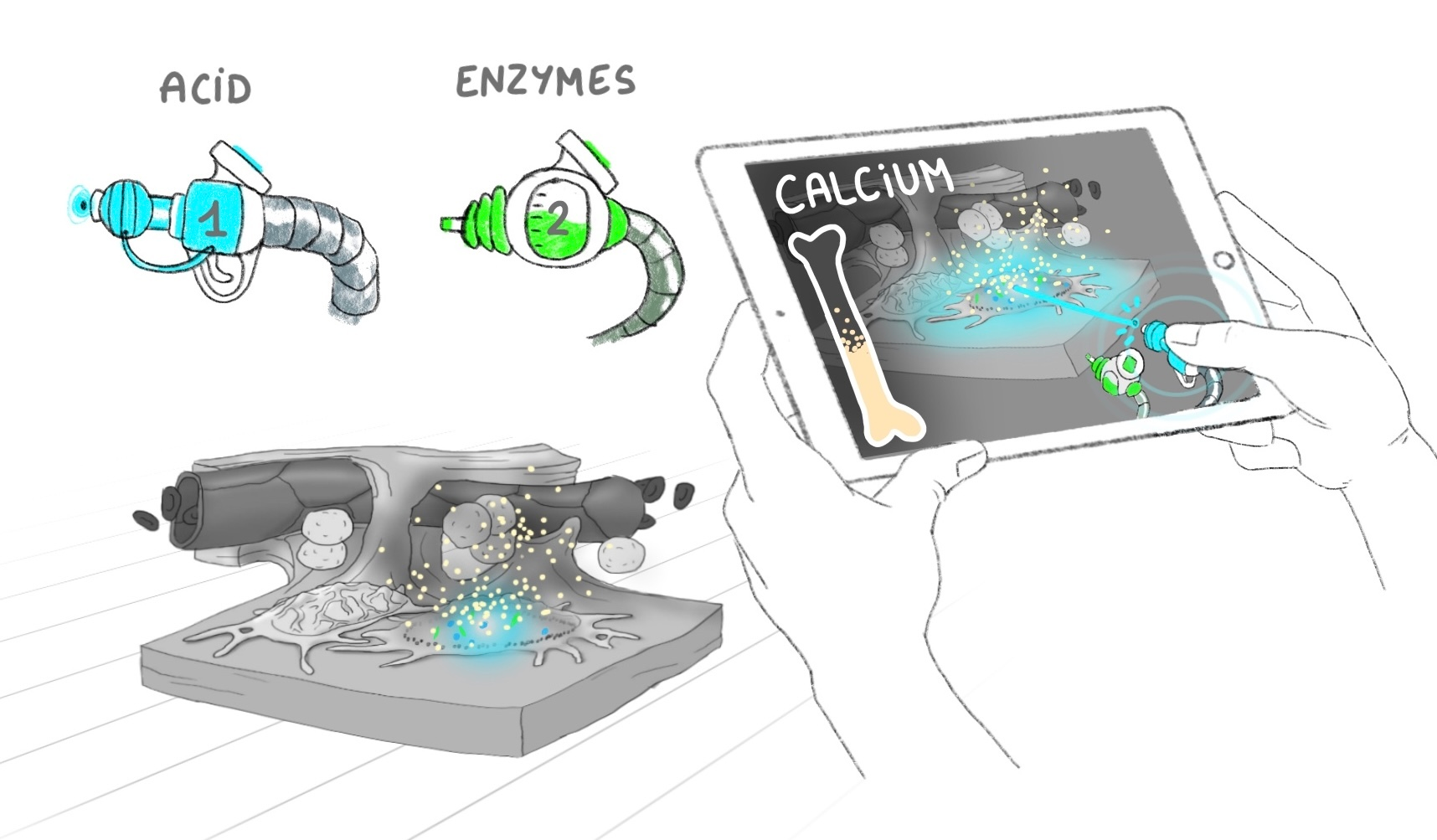
b)
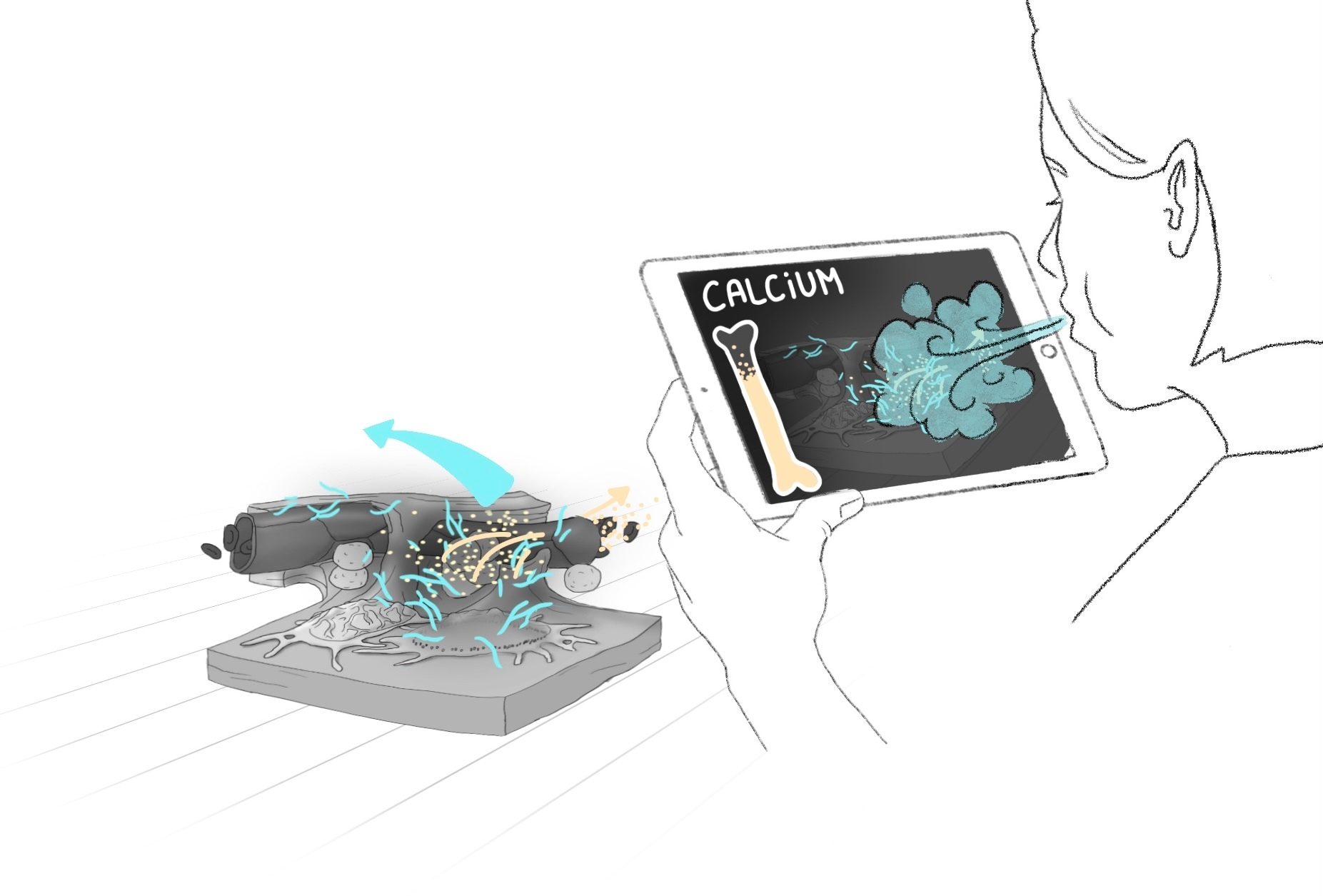


Figure S4: The second mini-game covered the formation of the lacuna. This minigame corresponded to storyboard 2-11, 2-12a, 2-12b, 2-13 (see Appendix 2D). In this minigame, the osteoclast demineralized the bone and degraded the collagen with enzymes. Then the calcium was returned to the bloodstream. a) The user aims the AR gun at either pieces of calcium to pulverize it with acid or degrade collagen with enzymes. In the lacunae we can see calcium and collagen that must be aimed at correctly. b) When the lacunae fills with debris, the user must blow to make the calcium (and degraded collagen fly away out of the lacunae).

a)
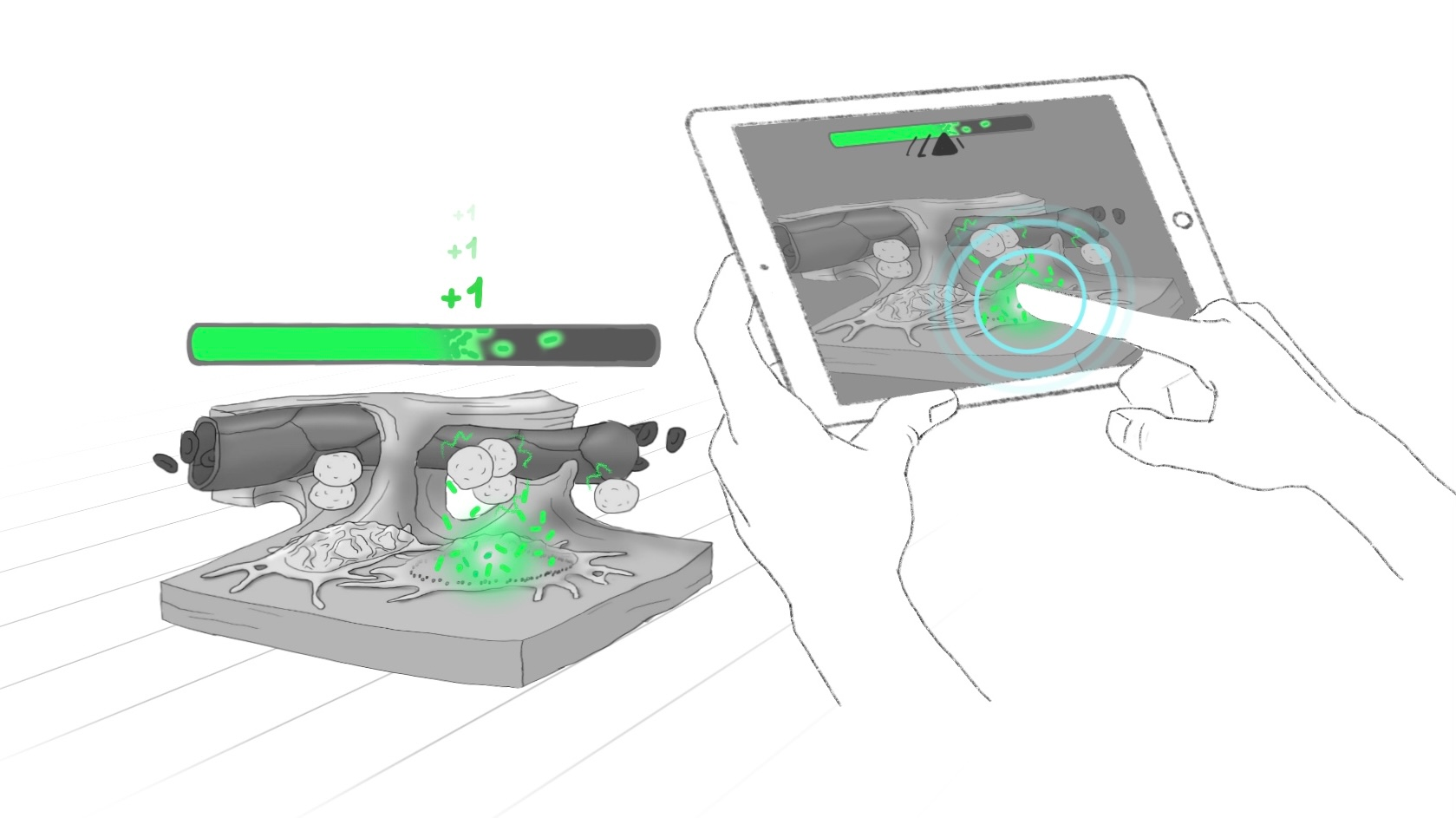
b)
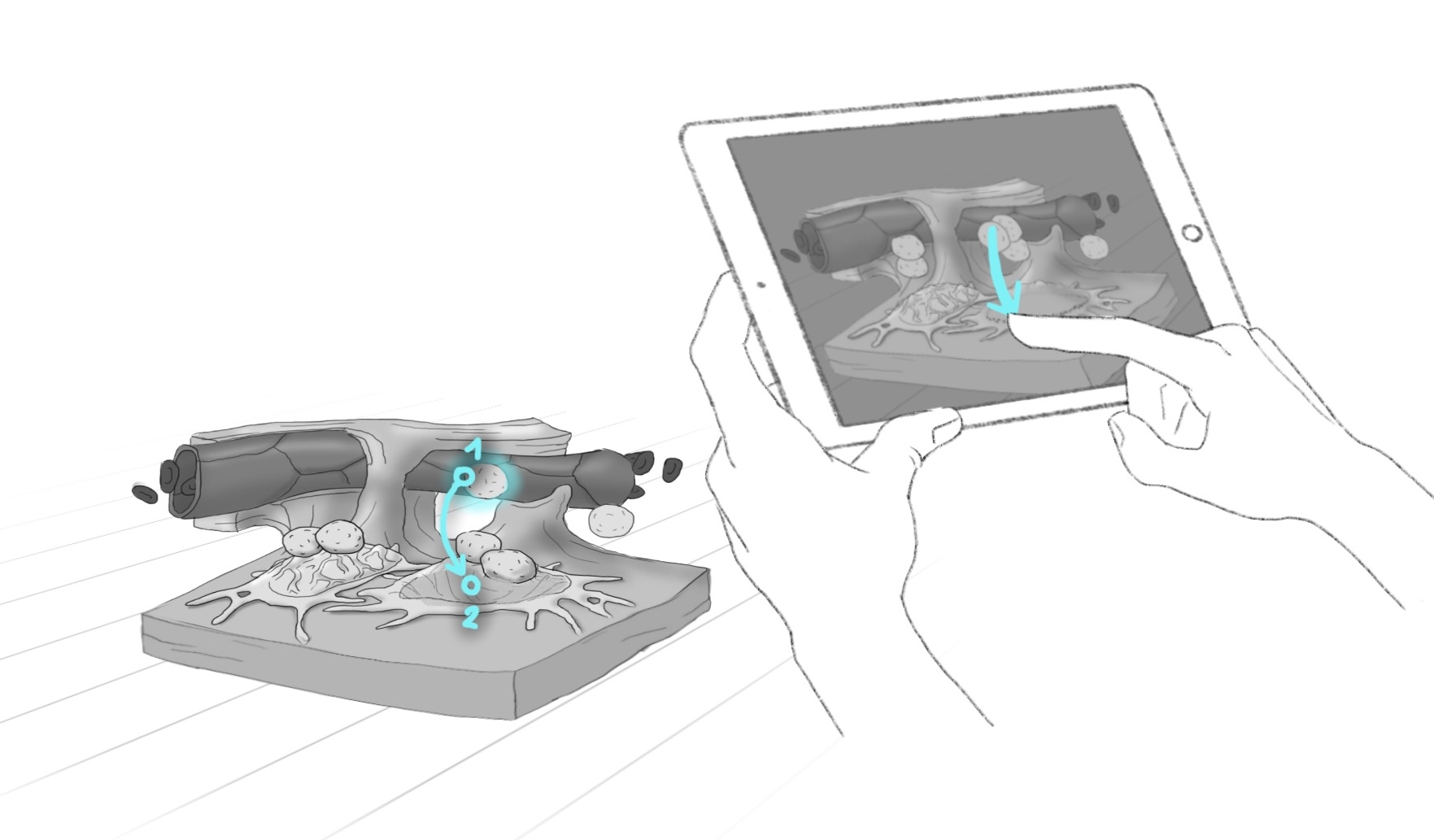


Figure S5: The third mini-game covered the arrival of osteoblasts. This minigame corresponded to storyline and storyboard 2-14, 2-15, 3-16, 3-17 (see Appendix 2D). In this minigame, the osteoclast secreted Vascular Endothelial Growth Factor (VEGF) to attract osteoblast precursor cells. As the osteoclasts performed apoptosis, the osteoblast precursor cells cleaned up and transform into osteoblasts. a) The user tapped the osteoclast to release ephrins and attract osteoblast precursor cells. Once the bar was filled, the osteoclast performed apoptosis and the osteoblast precursor cells appeared from the blood vessel. c) The osteoblast precursor cells needed to be pushed into the lacunae The osteoblast precursor cells stuck into the lacunae and transformed into osteoblasts.

#### S1.2 Encyclopedia

The simplified gameflow omitted certain details of the scientific processes and we complemented the gamified process with accurate descriptions in an encyclopedia that can be opened at any time, see Figure S29. The encyclopedia was evaluated by several experts in the field for scientific accuracy.

a)
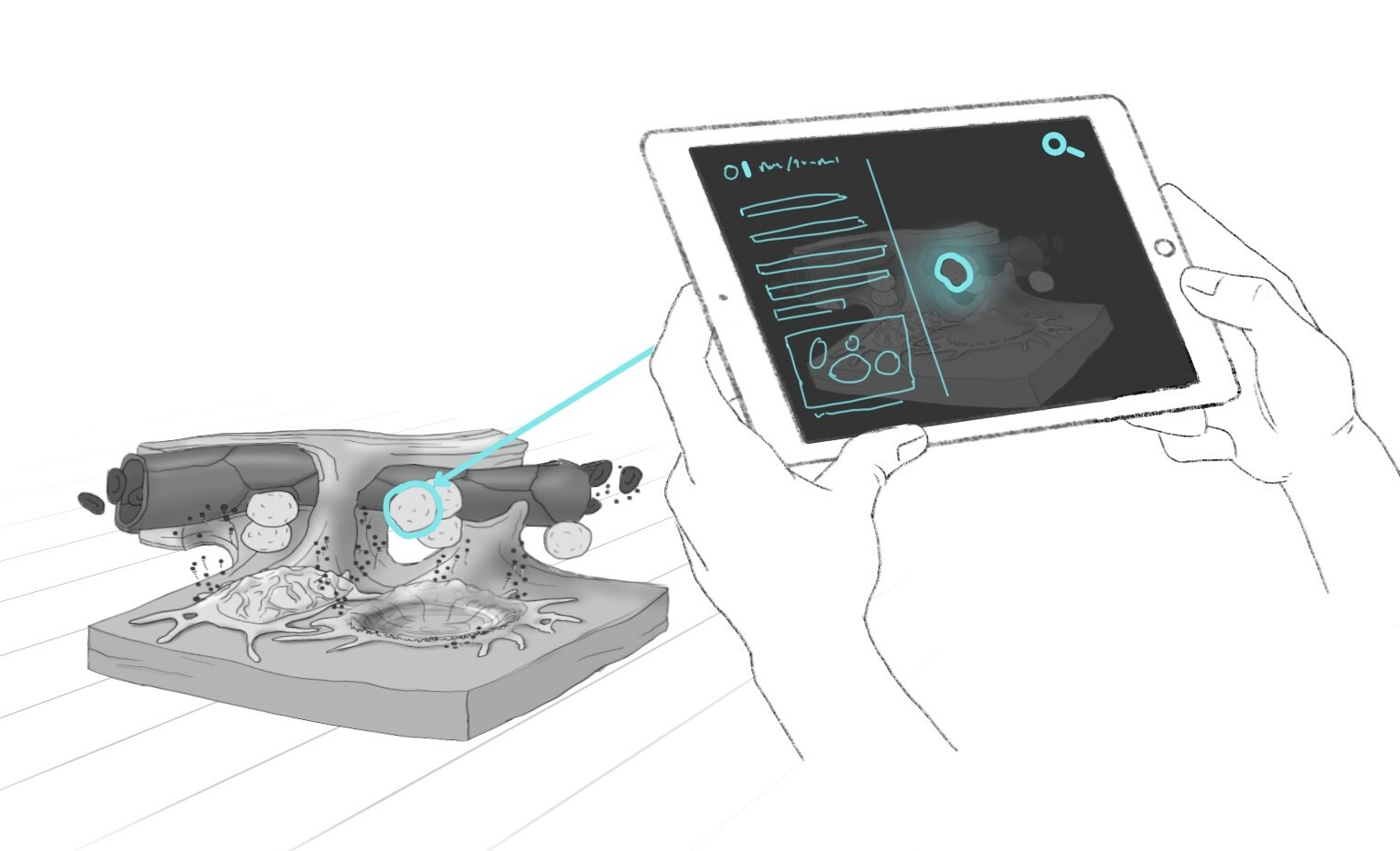
b)
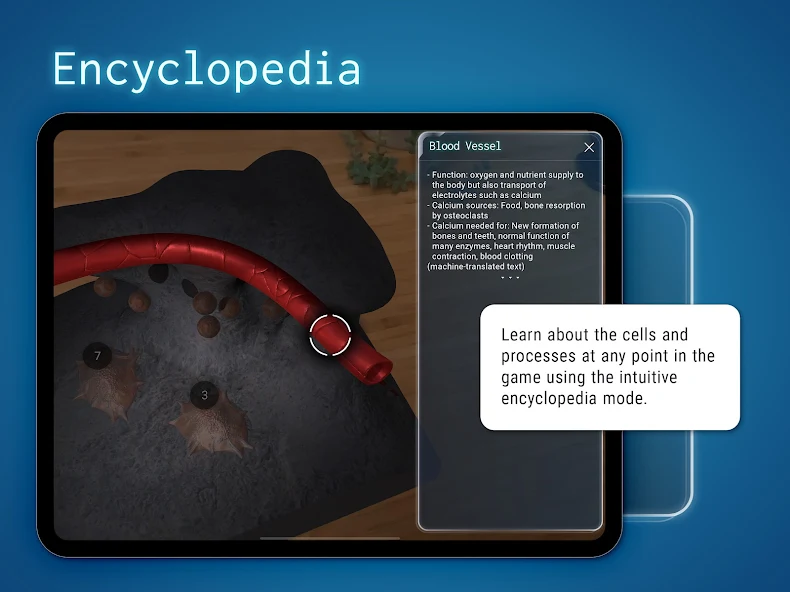


Figure S6: Encyclopedia. The user can aim the crosshairs in the center of the screen on objects in the scene to receive additional information. a) Sketch of interaction. b) Promotional material created by the Game Technology Center, used with permission.

### S2 Game Design Storyboard

**Storyline**

**Augmented Reality | Bone Remodelling**

Main actors: Hematopetic Stem Cell HSC, osteoclast precursers (OCP), membrane bound RANK

| Osteoclast |  | Bone resorption |
| --- | --- | --- |
| Osteoblast |  | Bone formation (present RANKL, produce OPG) |
| Osteocyte |  | Bone remodelling (present RANKL), release sclerostin and |
|  |  | prostaglandin PGE2 |
| Mediators: RANK |  | membrane bound receptor on osteoclasts |
| RANKL |  | activates osteoclasts |
| GM-CSF |  | granulocyte –macrophage colony stimulating factor released by osteoblast, mobilises osteoclast precursors |
| OPG |  | osteoprotegerin (soluble decoy receptor) |
| OCP |  | osteoclast precursor |
| OBP |  | osteoblast precursor |

Part 1 – Osteoclast activation

1. Hematopoetic stem cells (HSC) hibernate in the bone marrow cavity.
2. Parathyroid hormone signals to osteoblasts, that more blood calcium is needed.
3. Osteoblasts secrete GM-CSF which wake up the HSC, HSC divide and the daughter cells

(osteoclast precursors – OCP) move to the bone site

1. Osteoblasts detach from the bone and from a canopy with connection to the next blood vessel (separated by a thin membrane but not by endothelial cells)
2. Osteoblast reach out to the OCP and present RANKL on their cell membrane
3. OCP receive the RANKL signal and divide
4. Only cells in contact with bone can further differentiate to osteoclasts (OC)
5. 10-20 OCP fuse to from a mature OC

Part 2 - Bone resorption providing calcium

1. OC attached to osteopontin via a circle of integrins and form a resorption lacuna
2. OC forms a ruffled border
3. OC secretes acid (pH1) and acid resistant enzymes
4. Acid demineralises the bone, enzymes degrade collagen
5. Calcium is secreted to the canopy and further to the blood stream
6. OC finishes and reaches out to osteoblast precursors OBP via membrane bound Ephrins
7. OBP clean the resorption lacuna with membrane bound serine proteinases

Part 3 - New bone formation

16. BMP, which stands out of the bone surface is made soluble 17. BMP by chemotaxis attract more OBP and differentiates them to OB

18. OB fill the resorption lacuna with new bone.

| **Description** | **Picture** |
| --- | --- |
| General description of the basic picture with …   1. A bone block with extending structures. 2. A Blood vessel. 3. Erythrocytes streaming through the vessel, blending in and out on both sides. 4. Six Osteoblast precursor cells, gently swinging around their position. 5. Two osteoclast, the upper surface „bubbling”, the pseudopods extending and contracting slightly. | 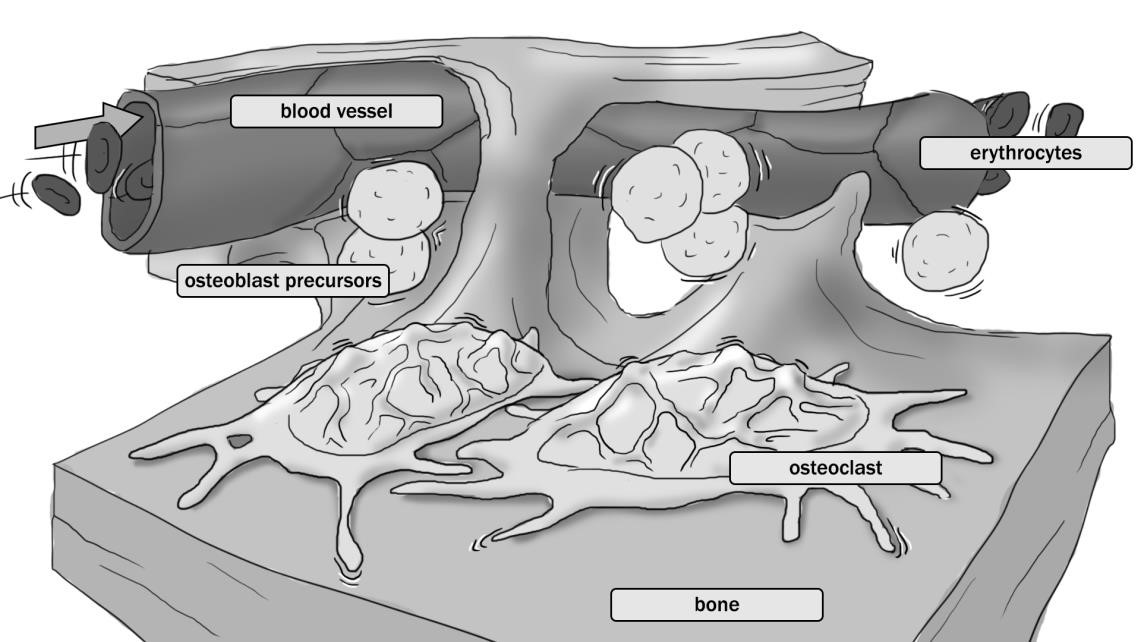 |

| **Description** | **Picture** | |
| --- | --- | --- |
| *OC attached to osteopontin via a circle of integrins and form a resorption lacuna.*    One of the osteoclasts turns transparent, the inner region more than the outer rim. Due to the transparency, it’s possible to see the bone under the osteoclast.  A ring of small molecules blends in. | 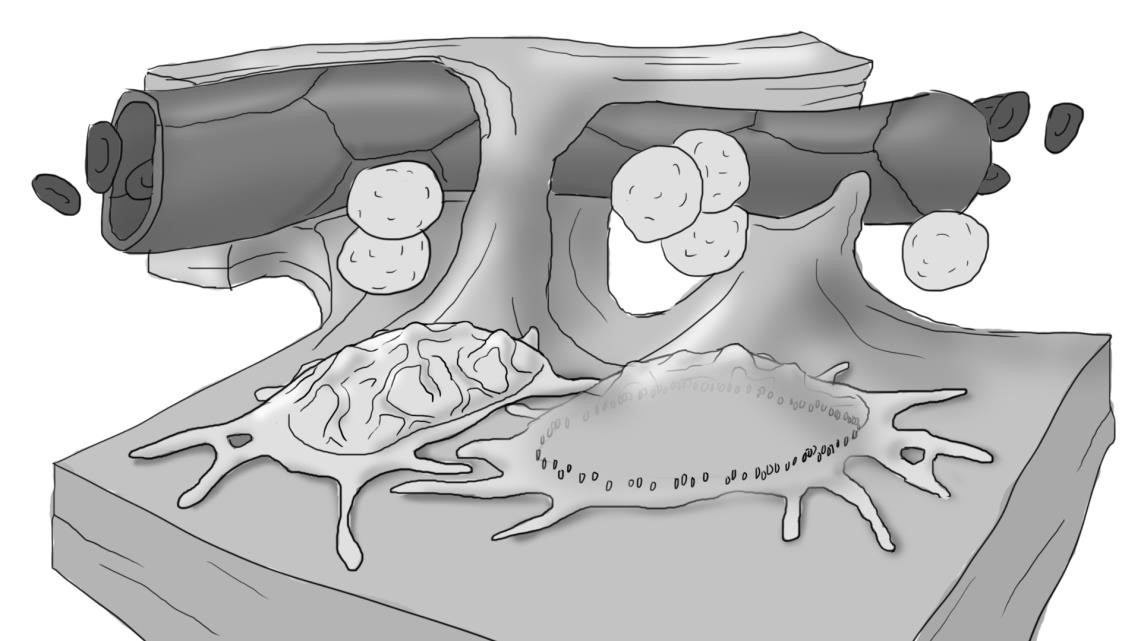 | |
| *OC forms a ruffled border.*    The osteopontin ring blends out.    The osteoblast forms a ruffed border. This is done by blending in a new model rather than a shape animation. | 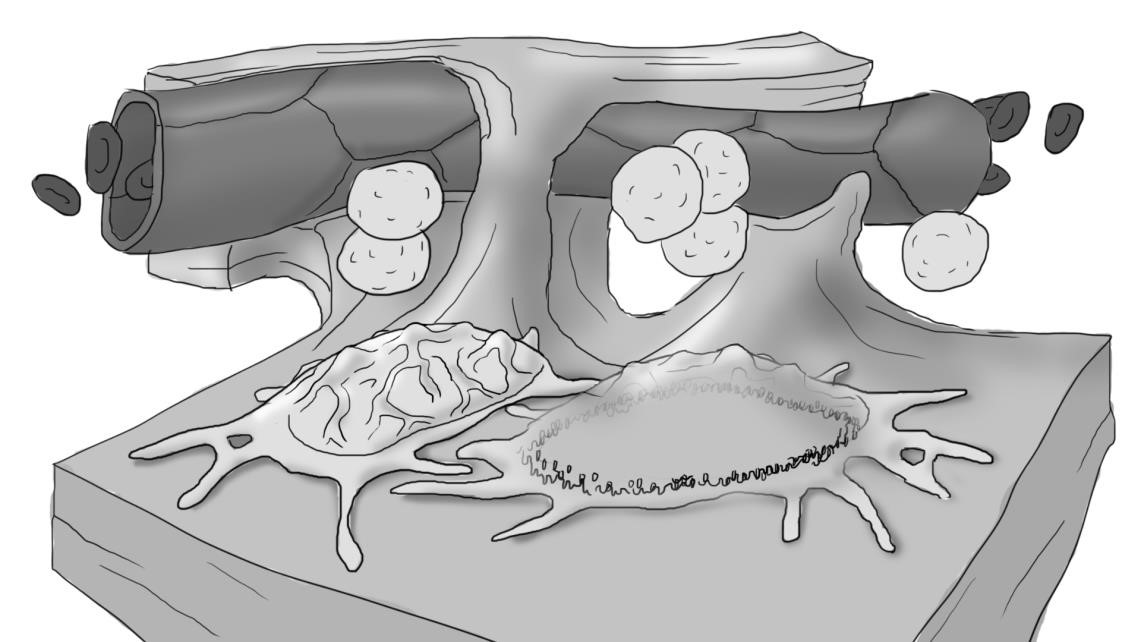 |  |

| **Description** | **Picture** |
| --- | --- |
| *OC secretes acid (pH1) and acid resistant enzymes.*    Little balls or organically shaped objects in two different colors blend in and move towards the bone. | 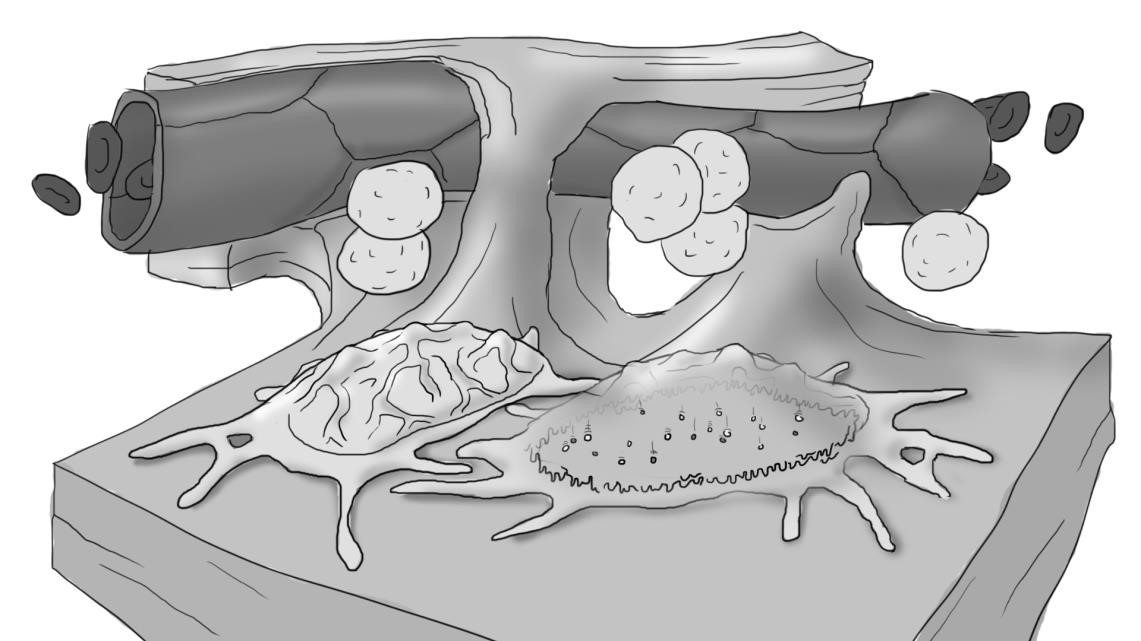 |
| *Acid demineralises the bone, …*    A depression forms in the bone (by shape animation, if possible).    In the first step, strings of collagen are visible. | 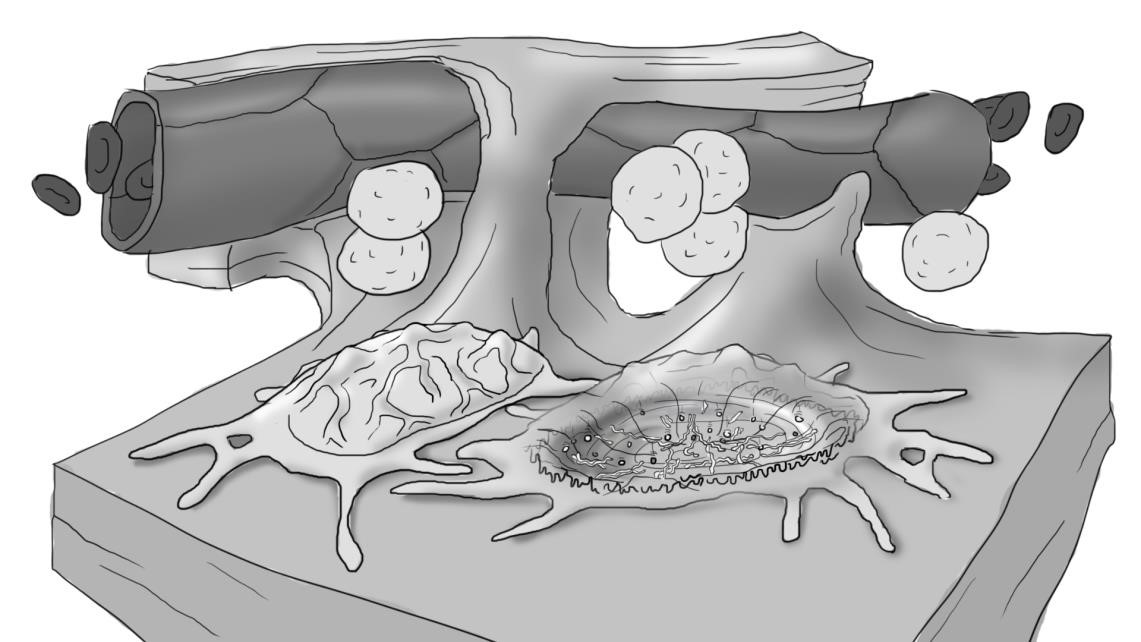 |

| **Description** | **Picture** |
| --- | --- |
| *… enzymes degrade collagen.*    The collagen fibers blend out. | 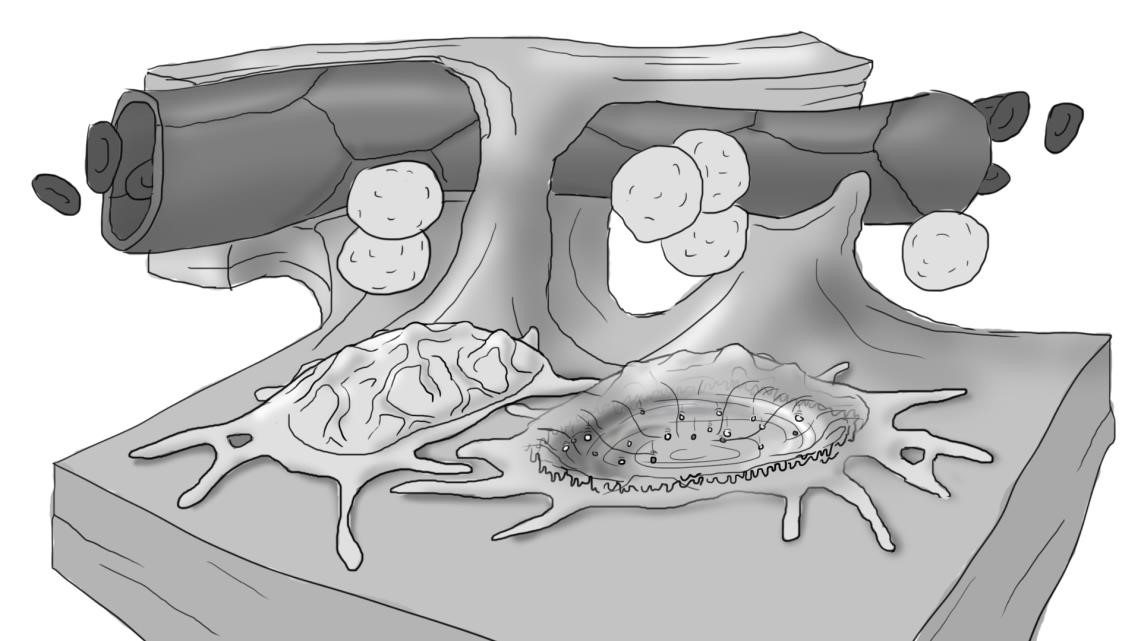 |
| *Calcium is secreted to the canopy and further to the blood stream.*    Many elements (balls or other geometrical shapes) blend in at the fringe of both osteoblasts.    They move towards the blood vessel and disappear in it.    Downstream, the same elements appear and move with the blood stream. They disappear like the erythrocytes. | 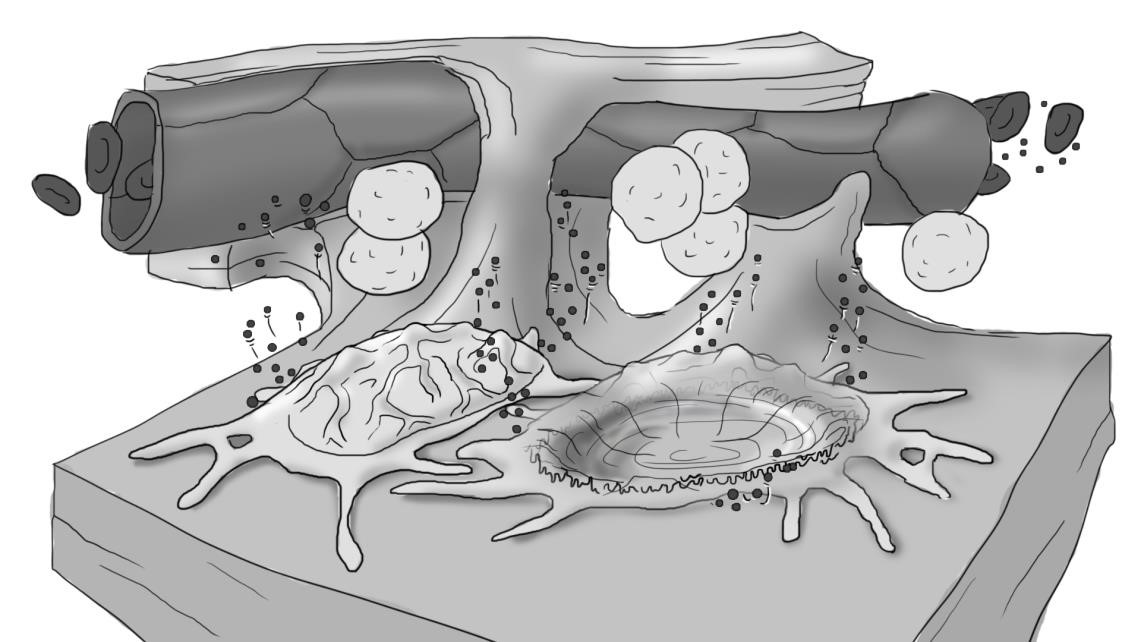 |

| **Description** | **Picture** |
| --- | --- |
| *OC finishes and reaches out to osteoblast precursors OBP via membrane bound Ephrins.*    The transparent osteoblast turns opaque again.    Some OBP cells move down in the direction of the osteoblasts until contact is reached. | 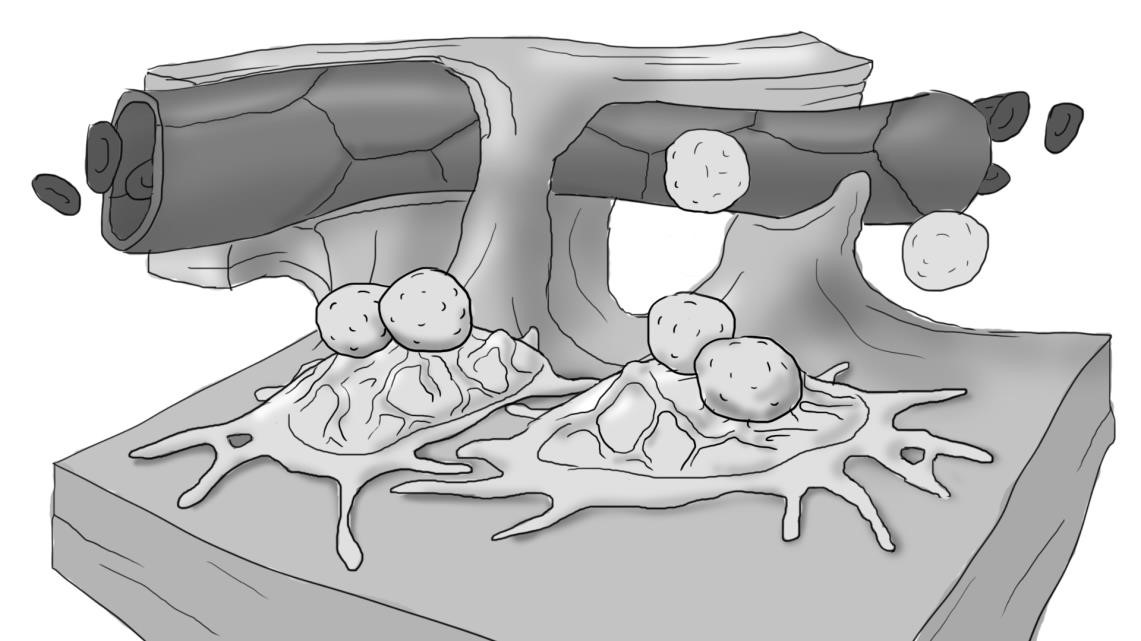 |

### S3 Bone remodeling mind map


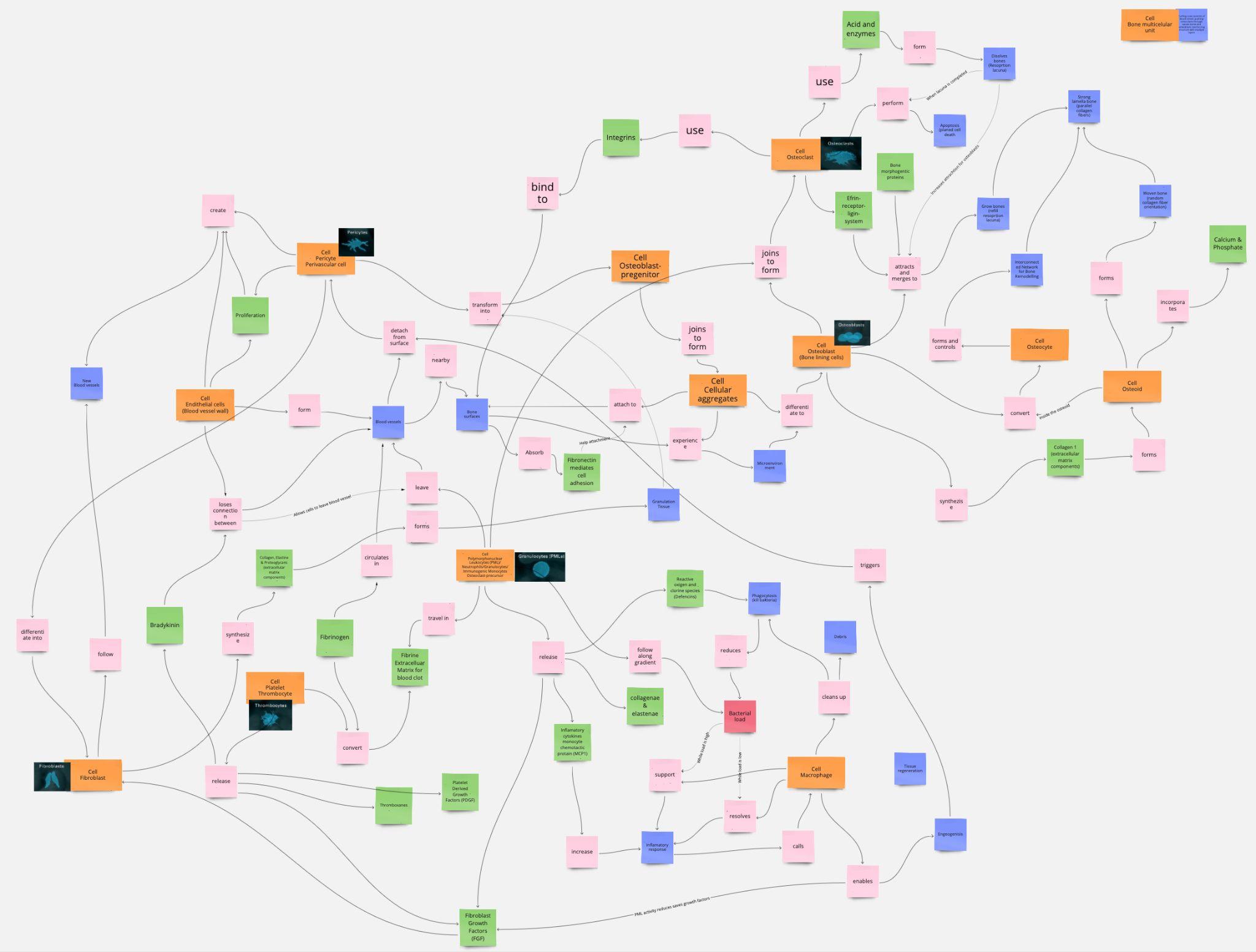


Figure S7: Mind map of all actors (orange), actions (pink), goals (green and red), and outcomes (blue). The mini-games map some of these to interactions for the user.

### References

1. Gruber R, Stadlinger B. Cell-Atlas - Visual Biology in Oral Medicine. Quintessenz Verlag; 2022. Available from: https://play.google.com/store/books/details?id=gPBmEAAAQBAJ ISBN:9783868676181

2. Grübel J, Thrash T, Aguilar L, Gath-Morad M, Chatain J, Sumner RW, Hölscher C, Schinazi VR. The hitchhiker’s guide to Fused Twins: A review of access to Digital Twins in situ in Smart Cities. Remote Sens (Basel) MDPI AG; 2022 Jun 27;14(13):3095. doi: 10.3390/rs14133095

3. Chatain J, Kapur M, Sumner RW. Three Perspectives on Embodied Learning in Virtual Reality: Opportunities for Interaction Design. Extended Abstracts of the 2023 CHI Conference on Human Factors in Computing Systems New York, NY, USA: Association for Computing Machinery; 2023. p. 1–8. doi: 10.1145/3544549.3585805

4. Bunt L, Greeff J, Taylor E. Enhancing Serious Game Design: Expert-Reviewed, Stakeholder-Centered Framework. JMIR Serious Games JMIR Serious Games; 2024 May 31;12(1):e48099. doi: 10.2196/48099

5. Metzmacher H, Rothnie F, Fayolle V, Sumner B, Zünd F. Using augmented reality games to communicate topics in Artificial Intelligence. 54th International Simulation and Gaming Conference (ISAGA 2023) ISAGA; 2023. p. 282–291. Available from: https://www.research-collection.ethz.ch/handle/20.500.11850/634829 [accessed May 31, 2024]

6. Colombo G, Grübel J. The Spatial Performance Assessment for Cognitive Evaluation (SPACE): A Novel Game for the Early Detection of Cognitive Impairment. Extended Abstracts of the 2023 CHI Conference on Human Factors in Computing Systems New York, NY, USA: Association for Computing Machinery; 2023. p. 1–6. doi: 10.1145/3544549.3583828

7. Grübel J. The design, experiment, analyse, and reproduce principle for experimentation in virtual reality. Frontiers in Virtual Reality frontiersin.org; 2023;4. doi: 10.3389/frvir.2023.1069423

8. Schade C, Stagge A. Paper Prototyping as a Method for the Evaluation of Serious game Concepts. search.proquest.com; 2020. doi: 10.34190/GBL.20.089

9. Winn B, Heeter C. Resolving conflicts in educational game design through playtesting. Innovate: Journal of Online Education learntechlib.org; 2006 Dec 1;3:6. Available from: https://www.learntechlib.org/p/104284/
