# Supplementary material for: Applying Augmented Reality to Convey Medical Knowledge on Osteoclasts to Users of a Serious Game: Vignette Experiment
Source: JMIR Serious Games. 2025 Jun 16;13:e64751. doi: 10.2196/64751 (PMC12185033; doi:10.2196/64751)
Supplement: Multimedia Appendix 2 — Usability study of the serious game. [file games-v13-e64751-s002.docx]

**Applying Augmented Reality to Convey Medical Knowledge on Osteoclasts to Users of a Serious Game: A Vignette Experiment**

## Multimedia Appendix 2: Usability study

### S4 Usability methodology

The usability study had two goals. Firstly, to validate that users were able to experience the full content of the game. For an optimal learning experience, we wanted to ensure that users understood the game mechanisms, so that we were not testing their skills in operating the game instead of their learning outcomes [1]. Therefore, we investigated how well the users were able to operate the game according to the design and whether users were satisfied with their game experience.

We investigated the usability of AR osteoclasts with participants from two sources, a publicly circulated version of the game and a controlled experiment. First, the game was made available with a QR code in the book. This encouraged expert users and students to play the game as they used the book. Engagement with the game in the book was optional. We evaluated this with a voluntary survey after playing the game that resembled the survey from the student learning experiment. Participants gave informed consent before starting the survey. Second, we used our learning experiment to evaluate the usability in a controlled context (Figure S8). The recruitment for in-person participants is detailed in the section on the student learning experiment. The usability study was only conducted with the participants who received the AR game.


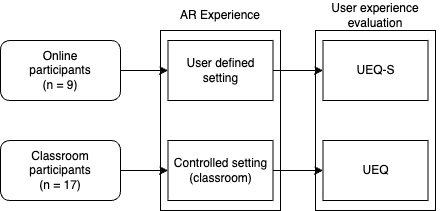


Figure S8: The protocol for the usability study. We recruited users from a course (n=17) and online (n=9). All participants have completed the game.

The survey after the AR experience evaluated the design of our game in terms of user experience. For the course students, we employed the short version of the User Experience Questionnaire (UEQ) [2,3], that is the UEQ-S [4], to increase retention [5]. These questionnaires allowed a distinction between objective (pragmatic) and subjective (hedonic) qualities of the experience. As the UEQ-S is focused on general software tools, we also used the Augmented Reality Immersion (ARI) questionnaire [6] to assess the level of participant immersion.

Finally, we complemented subjective participant evaluations with objective measures from the game. We assessed how well participants were able to use the game by looking at several key indicators. First, playtime was gathered by stages to assess how long people engaged with the mini-games as well as with the text. Second, activation of the AR scene was counted to check how well people could operate and interact with the AR scene. As we used marker-based AR, the scene was activated when the picture presented in the book was sufficiently within the tablet's field of view, and lost otherwise. Lastly, we assessed the user actions in the AR game as either being a “success”, a “miss”, or a “fail”. A success was an interaction that was performed according to the game design. A miss was an interaction close to the game design but which did not trigger the desired outcome, e.g., in the first part of Mini-Game 1, if a circle was drawn on the blood vessel rather than on the bone surface. Lastly, a “fail” was an interaction that was not planned in the game design. It is noteworthy that explorative behavior may be considered a “fail” in this paradigm.

### S5 Usability results

#### S5.1 Participant statistics

A total of 440 unique users were reported in the anonymized game reports. These users were not linked to survey respondents and no demographics were available. 26 users opted to provide us feedback via our voluntary survey. The 17 student participants in the experiment that played the game were included in these users. All users consented to take part in our survey and provide the user data from the game.

AR Osteoclasts was deployed globally via the Apple app-store and Google Play. The game has been installed and used in many countries across the world (Figure S9). For online participants, we did not gather demographic information and only used device-based information that participants shared.


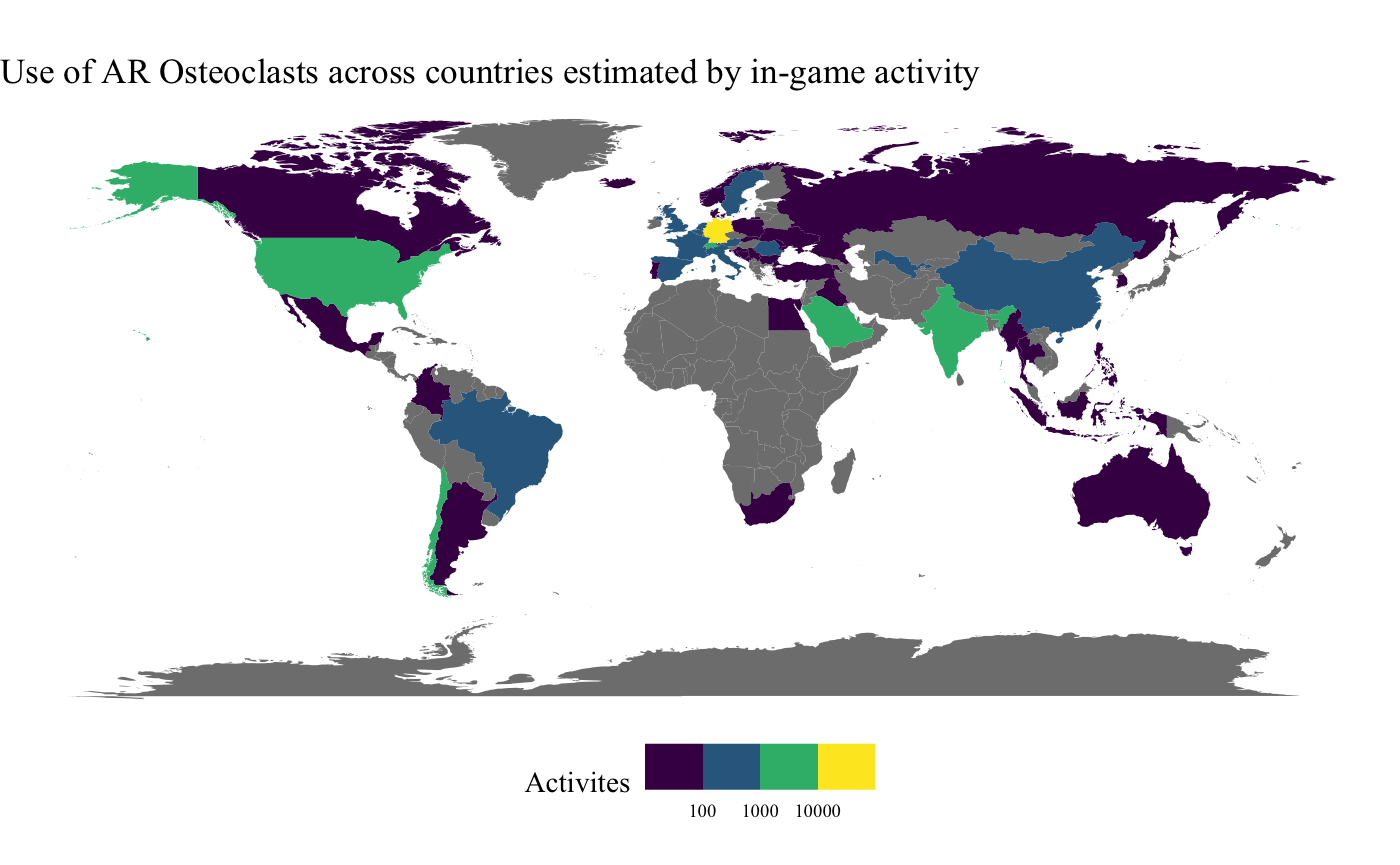


Figure S9: Approximation of engagement with AR Osteoclasts across the globe. People from 52 countries played AR Osteoclasts. Germany reports the highest engagement (country of the book press) but also India, the U.S., Chile, Switzerland and Saudi Arabia have seen more user activity.

#### S5.2 Evaluation of Outcomes

We evaluated user experience (UEQ-S) and AR immersion (ARI). We assessed the Augmented Reality Immersion (ARI) of participants and found that generally people online were more immersed. To some degree, this may be due to the classroom setting of the student experiment (Figure S10).

###
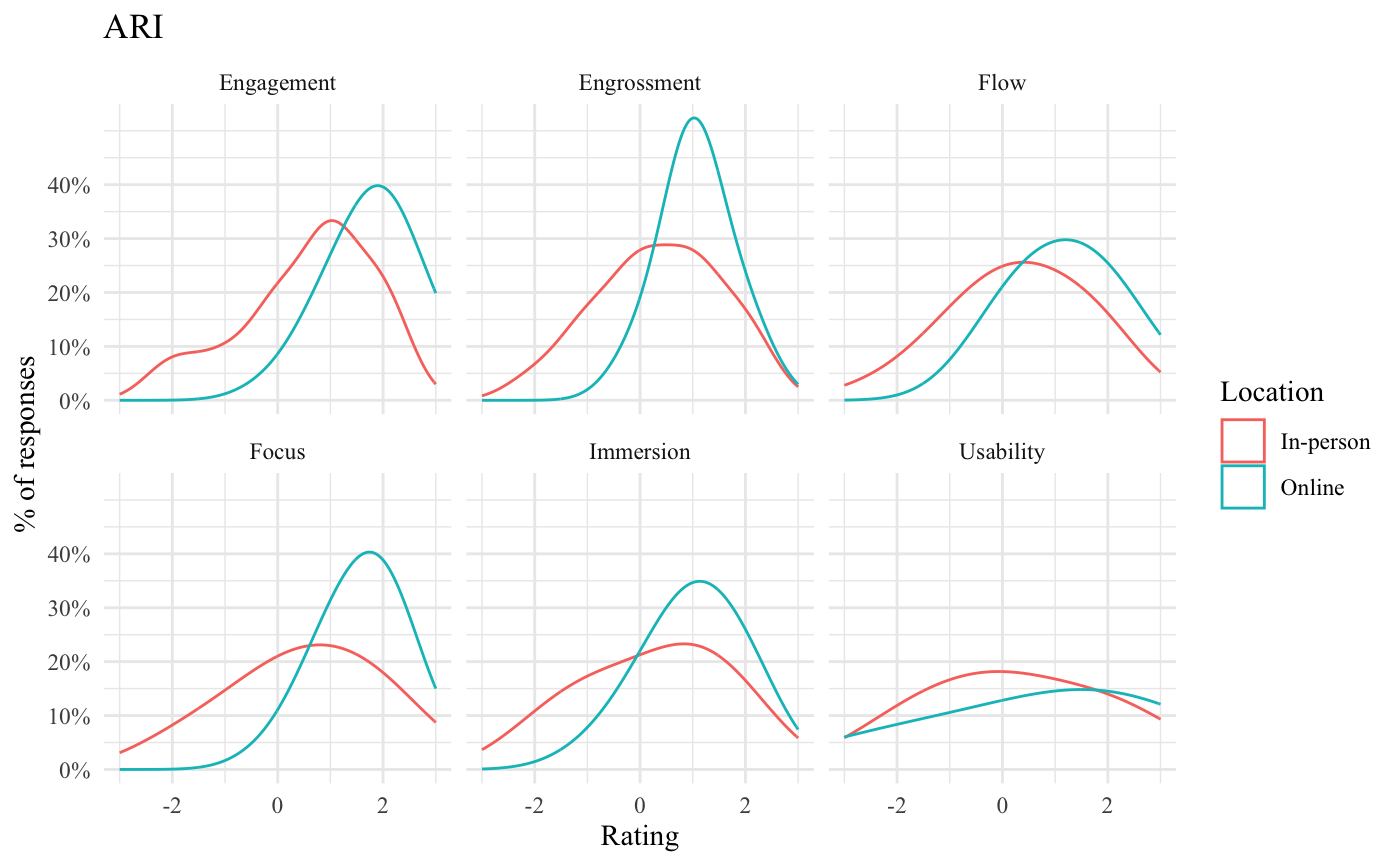


Figure S10: ARI results grouped by constructs from both the online participants and the student participants.

We also looked at the user experience with the UEQ-S survey. As the scales were subjective in judgment, the developers of UEQ-S have provided a benchmark that compared the usability across the average of thousands of other usability studies. Both online and offline, the game was perceived positively for its hedonic quality but was lacking somewhat in the pragmatic quality compared to these other usability studies (Figure S11).

a)
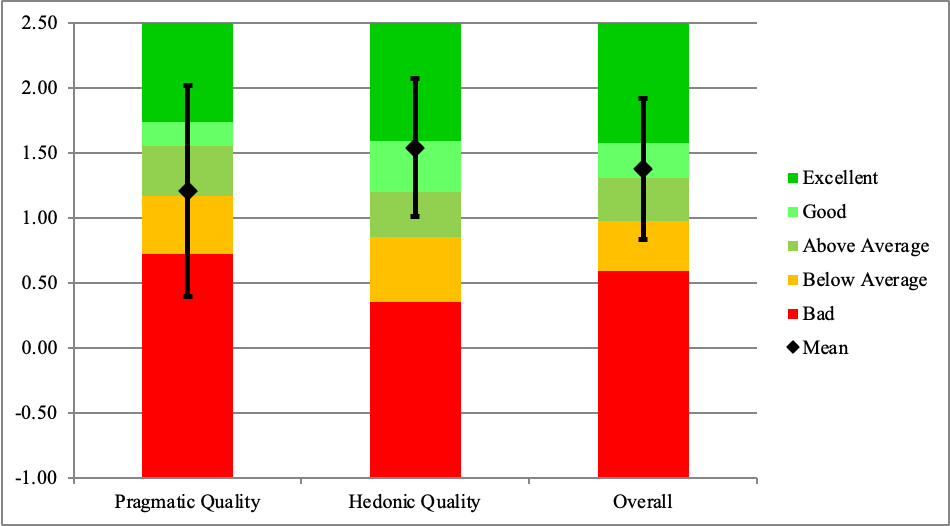
b)
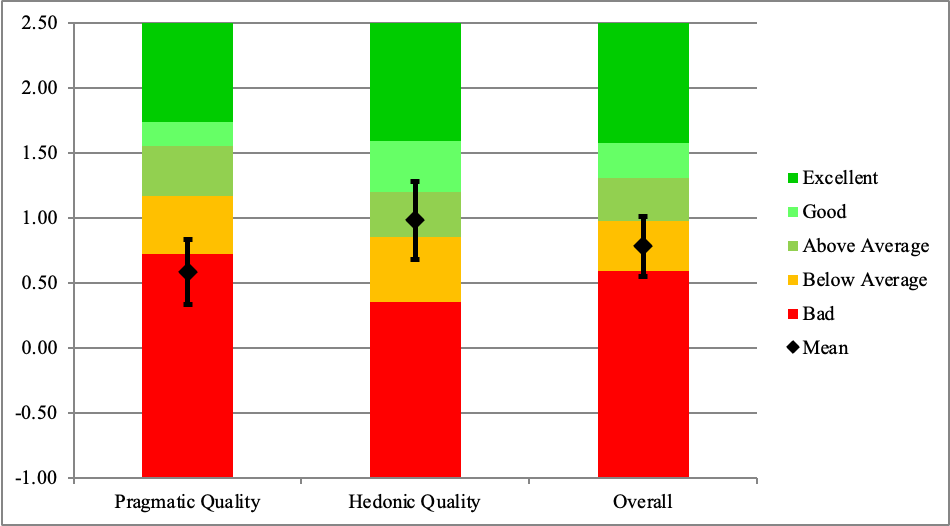


Figure S11: UEQ-S results from a) online respondents and b) in-person respondents. The online respondents had a more positive perception of AR Osteoclasts placing its hedonic quality significantly above the mean of UEQ-S whereas in-person respondents only rated it slightly above average. The pragmatic quality was not significantly above average.

#### S5.3 Technical usability

There are no guidelines on assessing technical suitability of serious AR games. However, we proposed three aspects that merit attention: the availability of AR features, the duration of play, and the actions that users take in the serious AR game. We tracked how often they lost and found the page on which the AR scene was anchored (Figure S12). We opted for this feature so that people who focused on the page got the AR experience from the first moment. In the tutorial, the scene was visible but was not the focus as the User Interface was explained. Only in the AR setup stage did we train participants how to focus on the page. After the training, we noted that for the rest of the time people effectively refocused on the page, bringing us to net zero between pages lost and found for the AR tracking.

###
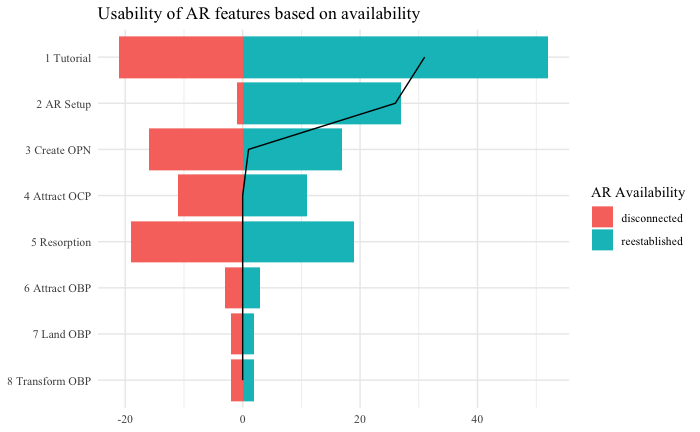


Figure S12: Usability of the AR features based on whether the AR was able to be displayed. Disconnected AR was caused when the camera could not see the book page. Connection was reestablished when the book page came into focus. During the Tutorial and the AR setup, most people acquired the AR display. During the game, the average was around zero, indicating that people were able to recover from losing the AR display.

The completion time by stages of the game revealed three patterns of interest (Figure S13). The average play time started off as a normal distribution, but as the stages progress it transformed into a trimodal distribution. At the end of the game, we had three distinct peaks at t=3 minutes, t=8 minutes, and t=16 minutes. We refer to these as short, average and extensive playtimes, respectively. These playtimes were indicative of playstyles[7] involving trying to play the game as fast as possible, engaging with the content as presented, or trying to explore every possible aspect of the game. The tail probabilities indicated that about half of the participants belonged to the average playtime group (p=0.5) whereas both other groups had a tail probability of around 10% (p=0.1).


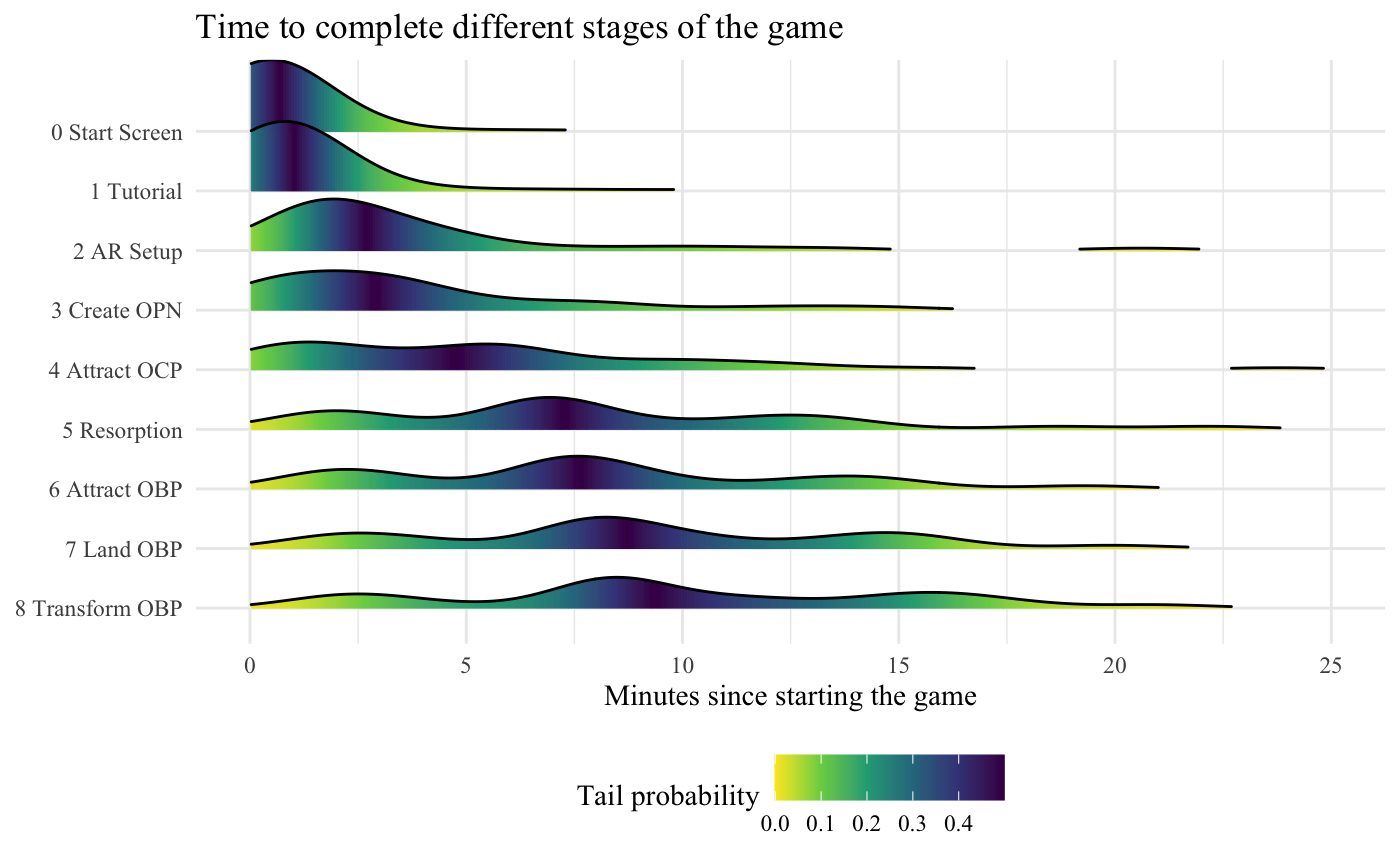


Figure S13: The stages of the game shown from top to bottom. The time to complete is shown across all participants. The height of the ridges shows how many participants completed the stage at a specific minute. The colors are based on the empirical cumulative distribution function indicating that 95% of participants fall in the blue to green area and only 5% fall in the yellow tones. We can see that the main cohort played the game in 8 minutes and that there were a few fast players completing it in 3 minutes and slow players completing the game in 16 minutes.

We showed the user interactions by game stage to investigate how easily participants were able to engage with the content (Figure S14). We observed that most failures to interact happened in the first game stage. There were two potential causes, either people did not yet understand what to do or they were so curious about the novel environment that they randomly interacted with anything. Furthermore, half the stages exhibited “miss” rates of nearly 50%. This could indicate that people had difficulty with the user interactions such that they understood what to do but were not able to do it correctly. Finally, two stages had success rates above 80% indicating that participants understood the tasks and performed them correctly.

However, we would like to better understand the “fails” and “misses”. Therefore, we investigated the user interactions by playtime leaning on the trimodal distribution of timing (Figure S14). For short playtime, we found very low failure rates and distinctly higher success rates than for the overall group. For the average playtime, we found that most failures were concentrated in this group. Lastly, we found that the average player seemed to get more used to the game mechanics in the next stage where they exhibited a high miss rate instead of a failure rate. Overall, many misses in the last stages were accrued by the extensive playtimes.


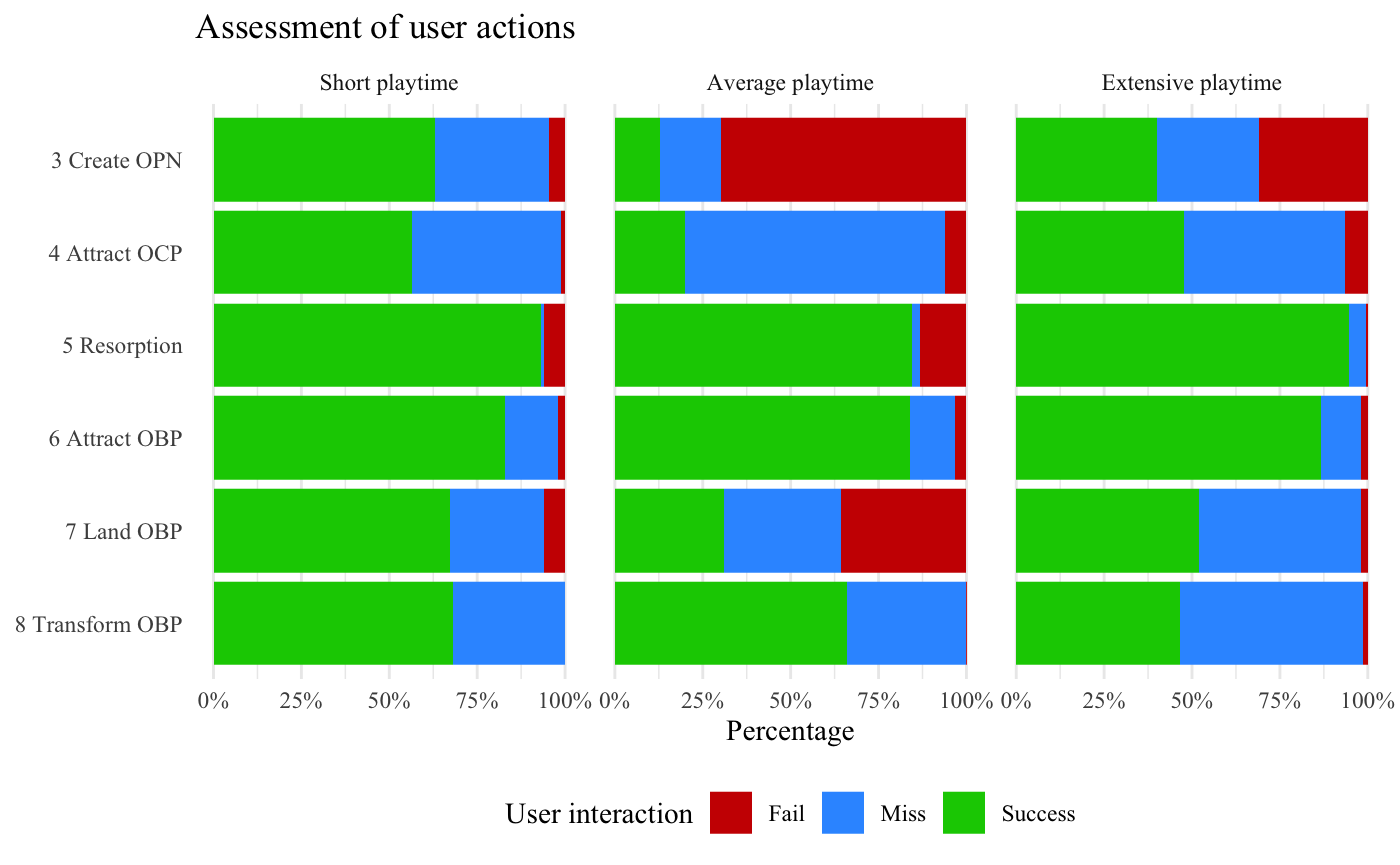


Figure S14: Short play times were related with higher success rates and extensive playtimes had higher miss rates. Most failures occurred with average playtime, especially at the beginning of the game.

We also looked at the interaction with the encyclopedia to gauge how deeply people engaged with the further learning context. We found that people were interacting with the encyclopedia most in the beginning and end of the game, indicating that they attained some kind of flow [8]. The encyclopedia would interrupt the flow around stage 6 and 7 and most participants opted not to engage during these stages.

####


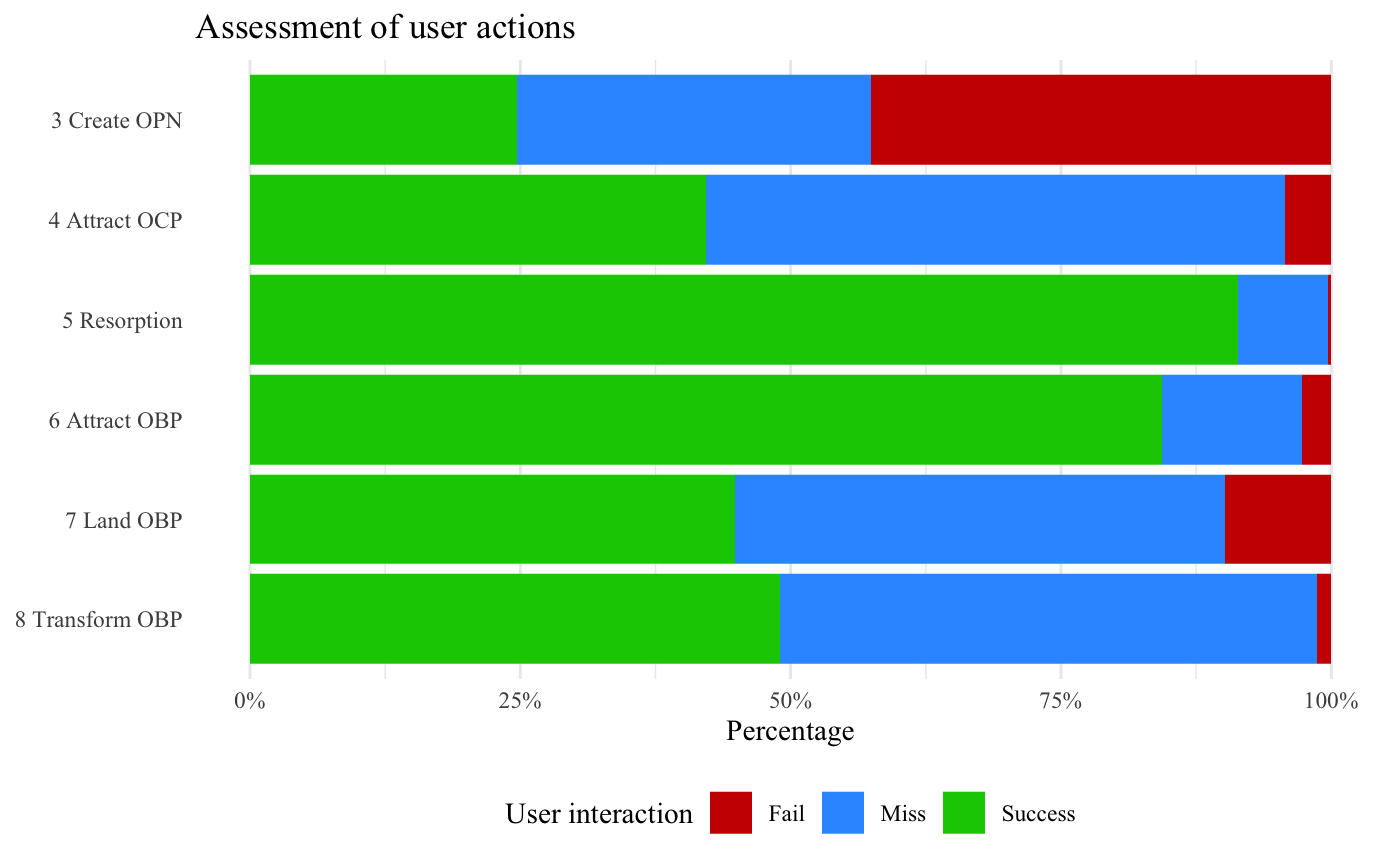


Figure S15: Assessment of user actions per stage. Users either successfully executed the interaction (blue), they missed by performing the right interaction but in the wrong context (green), or they failed by performing some other interaction. In the first game phase, the fail rate is highest as users needed to acclimate. Only the stages of “Resorption” and “Attract OBP” achieved very high success rates. All other stages required some learning before the tasks were done correctly.


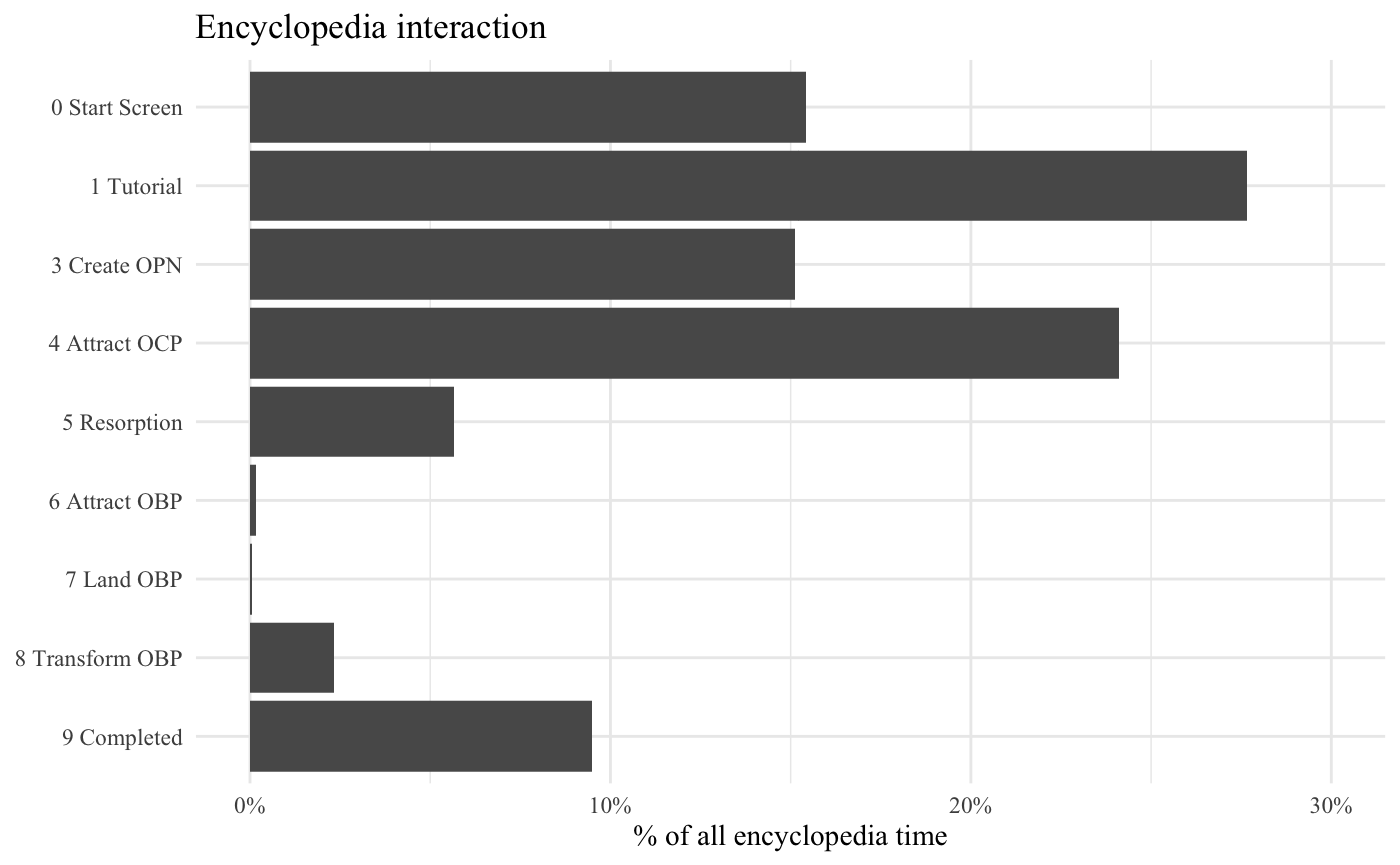


Figure S16: Encyclopedia interaction. A key feature of the game for in-depth knowledge access is the encyclopedia. We tracked the time they spend in the encyclopedia and people spend most time in the encyclopedia in the beginning and when they completed the game.

### S6 Usability discussion

We found that in-person participants rated the user experience as less new than online participants who were overall more positive towards the application. We explained this difference by the fact that the students took part in the experiment by default, whereas online participants approached the game on their own volition. Generally, the hedonic quality of the game was rated higher compared to the pragmatic quality. We interpreted this to indicate that the game was visually pleasing and a positive experience. But sometimes the game mechanics may have been too loosely related to the scientific process that they represented. Interestingly, despite the limited appeal from user experience perspective, the game statistically improved motivation.

We complemented the user experience study with data collected from the game itself. Online participants allowed us to gain additional insights into users by increasing the number of participants from 19 to a total of 376 before attrition. The game attracted the most activity in Germany, India, the U.S., Chile, Switzerland and Saudi Arabia (Figure S9). We attribute the localities to the distribution of the accompanying book and the respective prevalence of the book as a tool in education and practice. We aimed for users to take about 10 minutes to complete the AR experience. Most participants played the game for approximately 8 minutes, with a trimodal distribution of playtime with minor peaks at 3 minutes and 16 minutes, indicating different playstyles for so-called speedrunners (i.e. completing the game as fast as possible) to completionists (i.e. exploring every possible aspect of a game) [9]. Keeping playstyles in mind may be important to improve participant engagement by catering to their different interactions with the game. We found that people had no problems with the AR setup of the game. However, our analysis of the user actions also indicated that average playtime was associated with most difficulties operating the game. One reason for this could be different prior knowledge of AR and tech-savviness. Experienced players tended to have less trouble operating a game [1] and could therefore explore more efficiently or more broadly. It would be beneficial to connect in-game activity with survey results in future research, however, our setup did not allow for this.

### S7 Comparison online-offline responses

Bonferroni corrected t-tests revealed that comparing in-person with online participants had no statistical significant effect (t = -0.115, *P*_Bonferroni_ = .999) and grouping AR-enhanced in-person participants with AR-enhanced online participants against Text-only in-person participants was also not statistically significant (t = 2.0119, *P*_Bonferroni_ = .103) although elevated and close to the threshold α = 0.1.


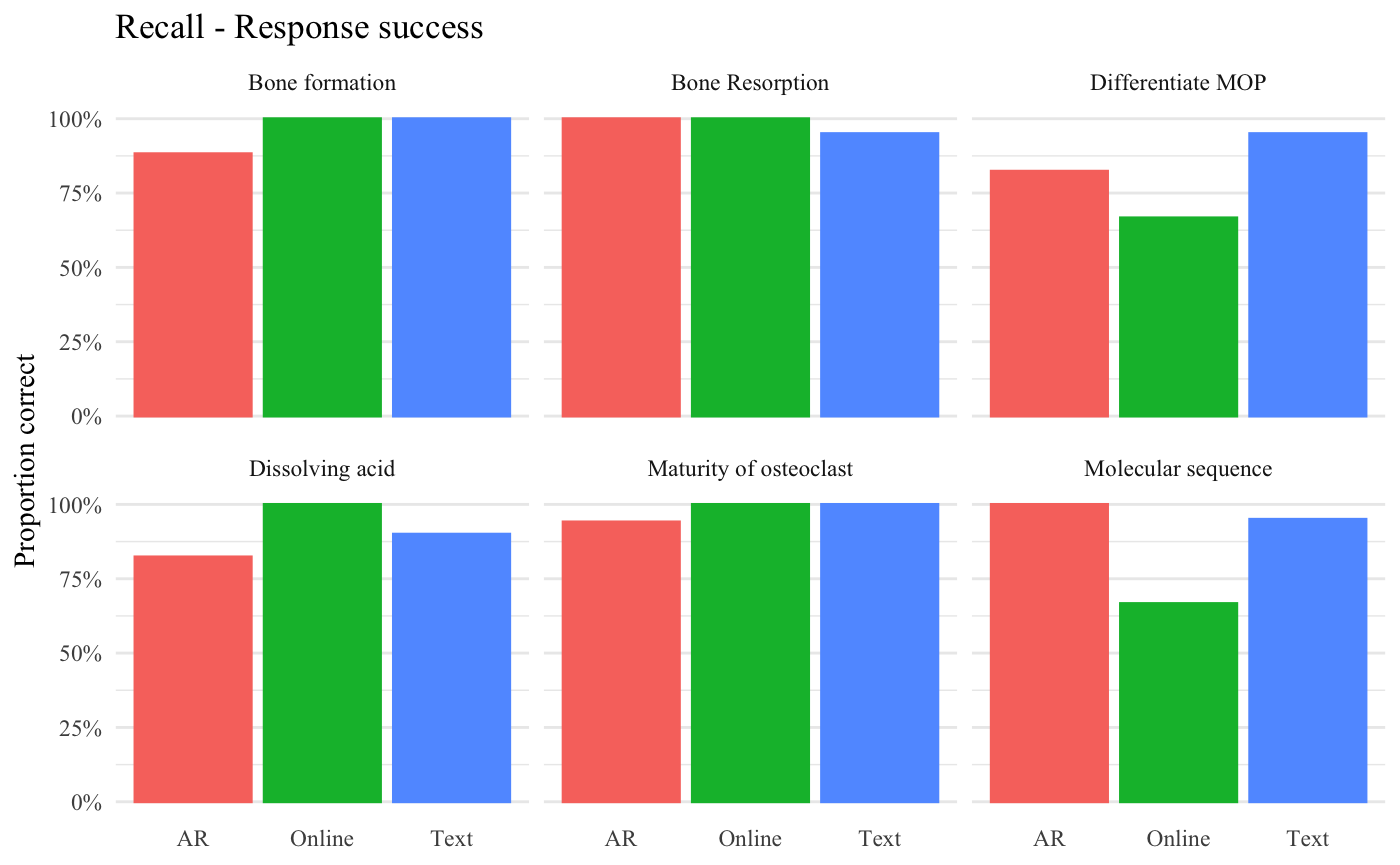


Figure S17: Recall comparison including online participants.


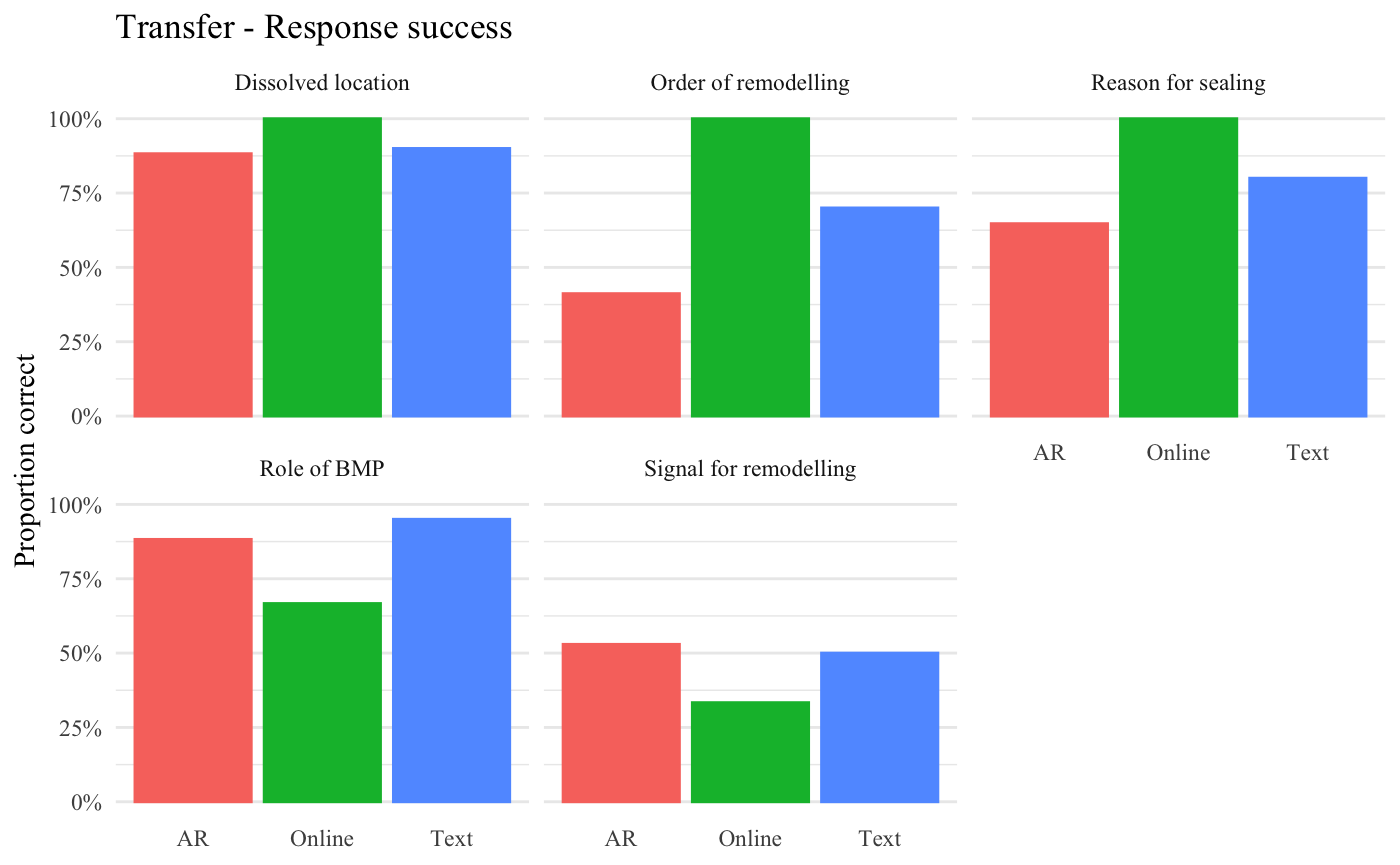


Figure S18: Transfer comparison including online participants.


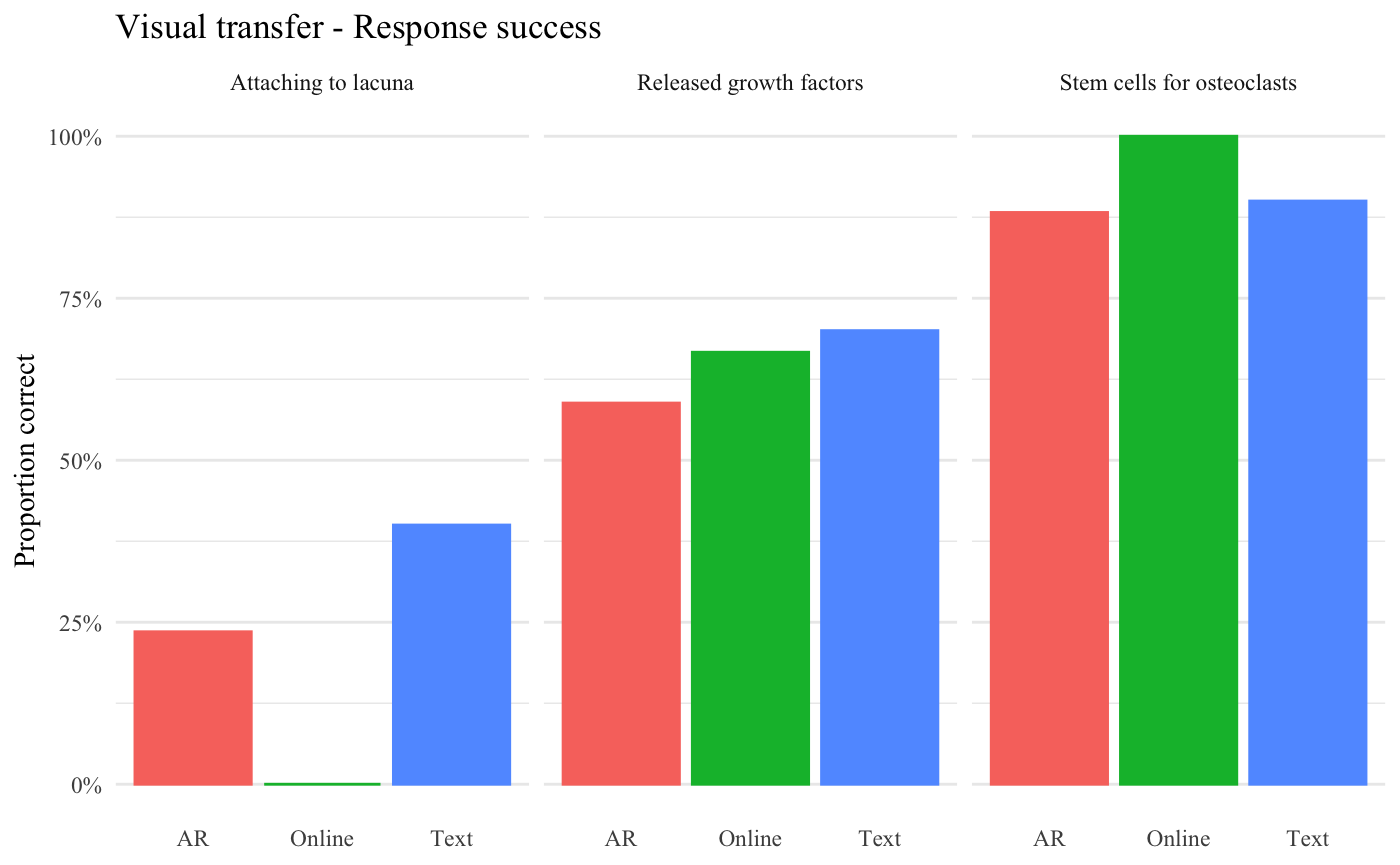


Figure S19: Visual Transfer comparison including online participants.

### References

1. Grübel J, Thrash T, Hölscher C, Schinazi VR. Evaluation of a conceptual framework for predicting navigation performance in virtual reality. PLoS One journals.plos.org; 2017 Sep 15;12(9):e0184682. PMID:28915266

2. Roberts ME, Stewart BM, Tingley D. stm: An R Package for Structural Topic Models. J Stat Softw jstatsoft.org; 2019 Oct 31;91:1–40. doi: 10.18637/jss.v091.i02

3. Laugwitz B, Held T, Schrepp M. Construction and Evaluation of a User Experience Questionnaire. HCI and Usability for Education and Work Springer Berlin Heidelberg; 2008. p. 63–76. doi: 10.1007/978-3-540-89350-9_6

4. Schrepp M, Hinderks A, Thomaschewski J. Design and evaluation of a short version of the user experience questionnaire (UEQ-S). Int J Interact Multimed Artif Intell IMAI Software - International Journal of Interactive Multimedia and Artificial Intelligence; 2017;4(6):103. doi: 10.9781/ijimai.2017.09.001

5. Schrepp M. A Comparison of SUS, UMUX-LITE, and UEQ-S. uxpajournal.org; 2023. Available from: https://uxpajournal.org/wp-content/uploads/sites/7/pdf/JUX_Schrepp_Feb2023_updated.pdf [accessed May 13, 2024]

6. Georgiou Y, Kyza EA. The development and validation of the ARI questionnaire: An instrument for measuring immersion in location-based augmented reality settings. Int J Hum Comput Stud Elsevier; 2017 Feb 1;98:24–37. doi: 10.1016/j.ijhcs.2016.09.014

7. Jaćević M. How the Players Get Their Spots: A Study of Playstyle Emergence in Digital Games. 2021 IEEE Conference on Games (CoG) IEEE; 2021. p. 1–8. doi: 10.1109/CoG52621.2021.9619067

8. Chen J. Flow in games (and everything else). Commun ACM New York, NY, USA: Association for Computing Machinery; 2007 Apr 1;50(4):31–34. doi: 10.1145/1232743.1232769

9. O’Brien SP. “Select the Type of Experience You Would Like to Have”: Exploring Player Roles and Role Affordance in Video Games. paj: The Journal of the Initiative for Digital Humanities, Media, and Culture paj-ojs-tamu.tdl.org; 2012;3. Available from: https://paj-ojs-tamu.tdl.org/paj/article/view/38
