# Supplementary material for: Applying Augmented Reality to Convey Medical Knowledge on Osteoclasts to Users of a Serious Game: Vignette Experiment
Source: JMIR Serious Games. 2025 Jun 16;13:e64751. doi: 10.2196/64751 (PMC12185033; doi:10.2196/64751)
Supplement: Multimedia Appendix 3 — Outlines of the survey questions fielded in Qualtrics. [file games-v13-e64751-s003.docx]

**Applying Augmented Reality to Convey Medical Knowledge on Osteoclasts to Users of a Serious Game: A Vignette Experiment**

**Multimedia Appendix 3**

Outlines of the survey questions fielded in Qualtrics. There are four parts. The *prestudy* is fielded before the participants play the game. The *mainstudy* is fielded after playing the game. The *poststudy 1 week* is fielded one week after playing the game and the *poststudy 1 month* is fielded one month after playing the game.

AR Osteoclasts Prestudy

Survey Flow

Block: Eligibility (5 Questions)

Standard: Identifier (2 Questions)

Standard: Medical background (6 Questions)

Standard: Demographics (5 Questions)

Standard: Gaming experience (2 Questions)

Standard: Self-awareness of achievements (6 Questions)

Standard: Attraction -> Concentration -> Performance (12 Questions)

| Page Break |  |
| --- | --- |

Start of Block: Eligibility

Q1.1
Dear students,


thank you for participating in this study. We are exploring new learning approaches based on Augmented Reality Educational Gaming. The study is run in cooperation between UZH and ETH Zürich.


At any time, you are allowed to stop participation in the study and you may request the removal of your data from our data collection. Your data will be deleted after the completion of the study. For more details, please contact Jascha Grübel under jgruebel@ethz.ch.


Before we start we ask you to verify that you are eligible to participate.

Q1.2 Are you 18 years or older?

- Yes (1)
- No (2)

Skip To: End of Survey If Q1.2 = No

Q1.3 Timing

First Click (1)

Last Click (2)

Page Submit (3)

Click Count (4)

| Page Break |  |
| --- | --- |

Q1.4 Are we allowed to link the data that you provide us here with the data that we will collect during class and after class?

- Yes (1)
- No (2)

Q1.5 Timing

First Click (1)

Last Click (2)

Page Submit (3)

Click Count (4)

End of Block: Eligibility

Start of Block: Identifier

Q2.1 Please write down your participant id that you generated on the piece of paper before this survey:

________________________________________________________________

Q2.2 Timing

First Click (1)

Last Click (2)

Page Submit (3)

Click Count (4)

End of Block: Identifier

Start of Block: Medical background

Q3.1 Do you have a background in dental medicine?

- Yes (1)
- No (2)

Q3.2 Timing

First Click (1)

Last Click (2)

Page Submit (3)

Click Count (4)

| Page Break |  |
| --- | --- |

Display This Question:

If Q3.1 = Yes

Q3.3 How many years have you been in dental medicine?

|  | 0 | 1 | 2 | 3 | 4 | 5 | 6 | 7 | 8 | 9 | 10 |
| --- | --- | --- | --- | --- | --- | --- | --- | --- | --- | --- | --- |

| () | 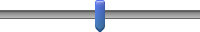 |
| --- | --- |

Q3.4 Timing

First Click (1)

Last Click (2)

Page Submit (3)

Click Count (4)

| Page Break |  |
| --- | --- |

Q3.5 We would like to know whether the topic of osteoclast is new to you. How experienced are you with osteoclasts?

- I am an expert on osteoclasts. (1)
- I have worked with osteoclasts. (2)
- I have only read about osteoclasts. (3)
- I have only heard about osteoclasts. (4)
- I have never heard of osteoclasts. (5)

Q3.6 Timing

First Click (1)

Last Click (2)

Page Submit (3)

Click Count (4)

End of Block: Medical background

Start of Block: Demographics

Q4.1 How old are you?

- 18 - 22 years old (1)
- 23 - 27 years old (2)
- 28 - 32 years old (3)
- 33 - 37 years old (4)
- 38 - 42 years old (5)
- 43 - 47 years old (6)
- 48 - 52 years old (7)
- 53 - 57 years old (8)
- 58 - 62 years old (9)
- 63 - 67 years old (10)
- 68 - 72 years old (11)
- 73 - 77 years old (12)
- older than 78 (13)

Q4.2 With what gender do you identify?

- Male (1)
- Female (2)
- Non-binary / third gender (3)
- Prefer not to say (4)

Q4.3 What is your highest completed degree?

- Doctorate (1)
- Master or similar (2)
- Bachelor or similar (3)
- High school or similar (4)
- Less than high school (5)

End of Block: Demographics

Start of Block: Gaming experience

Q5.1 How often do you play video games (desktop, console, mobile,...)?

- Daily (1)
- Several times a week (2)
- Several times per month (3)
- Every few months (4)
- Several times per year (5)
- Rarely (6)
- Never (7)

Q5.2 Timing

First Click (1)

Last Click (2)

Page Submit (3)

Click Count (4)

End of Block: Gaming experience

Start of Block: Self-awareness of achievements

Q6.1
In the following, we ask you to describe your study achievements so far. This will not be shared with your teachers.


Please answer the questions truthfully as this will help us evaluate the Augmented Reality Game.

Q6.2 In your studies so far, what would you estimate is your average grade according to the Swiss grade scale from 1 to 6. It would be great if you can look up your current grade average online with your phone.

|  | 1 | 1 | 2 | 2 | 2 | 2 | 3 | 3 | 3 | 3 | 4 | 4 | 4 | 4 | 5 | 5 | 5 | 5 | 6 |
| --- | --- | --- | --- | --- | --- | --- | --- | --- | --- | --- | --- | --- | --- | --- | --- | --- | --- | --- | --- |

| Swiss Grade () | 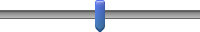 |
| --- | --- |

Q6.3 Did you estimate your grade or did you look it up?

- I estimated my current average grade. (1)
- I looked my current average grade up online. (2)

Q6.4 Timing

First Click (1)

Last Click (2)

Page Submit (3)

Click Count (4)

| Page Break |  |
| --- | --- |

Q6.5
Please estimate where you are in comparison to your study colleagues.


For example:

If you think that you have better grades than half of your colleagues, you would be at the 50 percentile.
If you think that you have better grades than than 90% of your colleages, you would be at the 90 percentile.

|  | 0 | 10 | 20 | 30 | 40 | 50 | 60 | 70 | 80 | 90 | 100 |
| --- | --- | --- | --- | --- | --- | --- | --- | --- | --- | --- | --- |

| Percentile () | 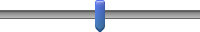 |
| --- | --- |

Q6.6 Timing

First Click (1)

Last Click (2)

Page Submit (3)

Click Count (4)

End of Block: Self-awareness of achievements

Start of Block: Attraction -> Concentration -> Performance

Q7.1 In the following, we present you with several statement and ask you to identify how much you agree or disagree with them. There is no "right" or "wrong". Please answer truthfully to help us evaluate the Augmented Reality Game.

Q7.3 I am on a good way to become a medical professional.

- I agree (1)
- I agree somewhat (2)
- I neither agree nor disagree (3)
- I disagree somewhat (4)
- I disagree (5)
- I don't know (6)

Q7.5 I can remember a lot when I read a text on a medical topic.

- I agree (1)
- I agree somewhat (2)
- I neither agree nor disagree (3)
- I disagree somewhat (4)
- I disagree (5)
- I don't know (6)

Q7.7 I can remember a lot when I look at images on a medical topic.

- I agree (1)
- I agree somewhat (2)
- I neither agree nor disagree (3)
- I disagree somewhat (4)
- I disagree (5)
- I don't know (6)

Q7.9 I can remember a lot when I can interact with content on a medical topic.

- I agree (1)
- I agree somewhat (2)
- I neither agree nor disagree (3)
- I disagree somewhat (4)
- I disagree (5)
- I don't know (6)

Q7.11 I usually find it difficult to focus when I read a text on a medical topic.

- I agree (1)
- I agree somewhat (2)
- I neither agree nor disagree (3)
- I disagree somewhat (4)
- I disagree (5)
- I don't know (6)

Q7.13 I usually find it difficult to focus when I look at images on a medical topic.

- I agree (1)
- I agree somewhat (2)
- I neither agree nor disagree (3)
- I disagree somewhat (4)
- I disagree (5)
- I don't know (6)

Q7.15 I usually find it difficult to focus when I can interact with content on a medical topic.

- I agree (1)
- I agree somewhat (2)
- I neither agree nor disagree (3)
- I disagree somewhat (4)
- I disagree (5)
- I don't know (6)

Q7.17 I perform better when I read a text on a medical topic.

- I agree (1)
- I agree somewhat (2)
- I neither agree nor disagree (3)
- I disagree somewhat (4)
- I disagree (5)
- I don't know (6)

Q7.19 I perform better when I look at images on a medical topic.

- I agree (1)
- I agree somewhat (2)
- I neither agree nor disagree (3)
- I disagree somewhat (4)
- I disagree (5)
- I don't know (6)

Q7.21 I perform better when I can interact with content on a medical topic.

- I agree (1)
- I agree somewhat (2)
- I neither agree nor disagree (3)
- I disagree somewhat (4)
- I disagree (5)
- I don't know (6)

Q7.22 Timing

First Click (1)

Last Click (2)

Page Submit (3)

Click Count (4)

End of Block: Attraction -> Concentration -> Performance

AR Osteoclasts Feedback INperson

Survey Flow

Block: Consent (7 Questions)

Standard: Identifier (2 Questions)

Branch: New Branch

If

If Did you play the Augmented Reality Educational Game "AR Osteoclasts"? Yes Is Selected

Standard: Game Aesthetics UEQ (4 Questions)

Standard: Augmented Reality Immersion (16 Questions)

Standard: CEI-II (2 Questions)

Standard: Recall (8 Questions)

Standard: Transfer (7 Questions)

Standard: Visual Transfer (11 Questions)

Standard: Qualitative answers (9 Questions)

Standard: IMMS (37 Questions)

Standard: Follow-up (3 Questions)

| Page Break |  |
| --- | --- |

Start of Block: Consent

Q1.1 Thank you for participating in this user study of our educational augmented reality game AR Osteoclasts. Before we start with the test, we would like to get your consent to use your data for scientific evaluation.

Q1.2 Can we use your answers to this survey to evaluate our game AR Osteoclasts?

- Yes (1)
- No (2)

Q1.3 Are you older than 18 years?

- Yes (1)
- No (2)

Skip To: End of Survey If Q1.3 = 2

Q1.4 Timing

First Click (1)

Last Click (2)

Page Submit (3)

Click Count (4)

| Page Break |  |
| --- | --- |

Q1.6 Did you play the Augmented Reality Educational Game "AR Osteoclasts"?

- Yes (1)
- No (2)

Q1.7 Are we allowed to link data from your game session with your answers here?

- Yes (1)
- No (2)

Q1.8 Timing

First Click (1)

Last Click (2)

Page Submit (3)

Click Count (4)

End of Block: Consent

Start of Block: Identifier

| 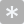 |
| --- |

Q2.1 Please write down your participant id that you generated on the piece of paper before the user study:

________________________________________________________________

Q2.2 Timing

First Click (1)

Last Click (2)

Page Submit (3)

Click Count (4)

End of Block: Identifier

Start of Block: Game Aesthetics UEQ

Q3.1 First, we would like to understand your user experience with the game. There is no "right" or "wrong" answer.

Q3.2 Timing

First Click (1)

Last Click (2)

Page Submit (3)

Click Count (4)

| Page Break |  |
| --- | --- |

| 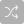 |
| --- |

Q3.3 Decide as spontaneously as possible which of the following conflicting terms better describes the game. There is no "right" or "wrong" answer.

|  | 1 (1) | 2 (2) | 3 (3) | 4 (4) | 5 (5) | 6 (6) | 7 (7) |  |
| --- | --- | --- | --- | --- | --- | --- | --- | --- |
| annoying |  |  |  |  |  |  |  | enjoyable |
| not understandable |  |  |  |  |  |  |  | understandable |
| creative |  |  |  |  |  |  |  | dull |
| easy to learn |  |  |  |  |  |  |  | difficult to learn |
| valuable |  |  |  |  |  |  |  | inferior |
| boring |  |  |  |  |  |  |  | exciting |
| not interesting |  |  |  |  |  |  |  | interesting |
| unpredictable |  |  |  |  |  |  |  | predictable |
| fast |  |  |  |  |  |  |  | slow |
| inventive |  |  |  |  |  |  |  | conventional |
| obstructive |  |  |  |  |  |  |  | supportive |
| good |  |  |  |  |  |  |  | bad |
| complicated |  |  |  |  |  |  |  | easy |
| unlikeable |  |  |  |  |  |  |  | pleasing |
| usual |  |  |  |  |  |  |  | leading edge |
| unpleasent |  |  |  |  |  |  |  | pleasent |
| secure |  |  |  |  |  |  |  | not secure |
| motivating |  |  |  |  |  |  |  | demotivating |
| meets expectations |  |  |  |  |  |  |  | does not meet expectation |
| inefficient |  |  |  |  |  |  |  | efficient |
| clear |  |  |  |  |  |  |  | confusing |
| impractical |  |  |  |  |  |  |  | practical |
| organized |  |  |  |  |  |  |  | cluttered |
| attractive |  |  |  |  |  |  |  | unattractive |
| friendly |  |  |  |  |  |  |  | unfriendly |
| conservative |  |  |  |  |  |  |  | innovative |

Q3.4 Timing

First Click (1)

Last Click (2)

Page Submit (3)

Click Count (4)

End of Block: Game Aesthetics UEQ

Start of Block: Augmented Reality Immersion

Q4.1 Please answer the following questions on how you perceived the game. There is no "right" or "wrong" answer.

Q4.2 Timing

First Click (1)

Last Click (2)

Page Submit (3)

Click Count (4)

| Page Break |  |
| --- | --- |

| 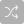 |
| --- |

Q4.3

|  | Strongly agree (1) | Agree (2) | Somewhat agree (3) | Neither agree nor disagree (4) | Somewhat disagree (5) | Disagree (6) | Strongly disagree (7) |
| --- | --- | --- | --- | --- | --- | --- | --- |
| I liked the activity because it was novel (1) |  |  |  |  |  |  |  |
| I liked the type of the activity (2) |  |  |  |  |  |  |  |

Q4.4 Timing

First Click (1)

Last Click (2)

Page Submit (3)

Click Count (4)

| Page Break |  |
| --- | --- |

| 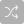 |
| --- |

Q4.5

|  | Strongly agree (1) | Agree (2) | Somewhat agree (3) | Neither agree nor disagree (4) | Somewhat disagree (5) | Disagree (6) | Strongly disagree (7) |
| --- | --- | --- | --- | --- | --- | --- | --- |
| I wanted to spend the time to complete the activity successfully (1) |  |  |  |  |  |  |  |
| I wanted to spend time to participate in the activity (2) |  |  |  |  |  |  |  |

Q4.6 Timing

First Click (1)

Last Click (2)

Page Submit (3)

Click Count (4)

| Page Break |  |
| --- | --- |

| 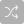 |
| --- |

Q4.7

|  | Strongly agree (1) | Agree (2) | Somewhat agree (3) | Neither agree nor disagree (4) | Somewhat disagree (5) | Disagree (6) | Strongly disagree (7) |
| --- | --- | --- | --- | --- | --- | --- | --- |
| It was easy for me to use the AR application (1) |  |  |  |  |  |  |  |
| I found the AR application confusing (2) |  |  |  |  |  |  |  |
| The AR application was unnecessarily complex (8) |  |  |  |  |  |  |  |
| I did not have difficulties in controlling the AR application (9) |  |  |  |  |  |  |  |

Q4.8 Timing

First Click (1)

Last Click (2)

Page Submit (3)

Click Count (4)

| Page Break |  |
| --- | --- |

| 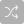 |
| --- |

Q4.9

|  | Strongly agree (1) | Agree (2) | Somewhat agree (3) | Neither agree nor disagree (4) | Somewhat disagree (5) | Disagree (6) | Strongly disagree (7) |
| --- | --- | --- | --- | --- | --- | --- | --- |
| I was curious about how the activity would progress (1) |  |  |  |  |  |  |  |
| I was often excited since I felt as being part of the activity (2) |  |  |  |  |  |  |  |
| I often felt suspense by the activity (8) |  |  |  |  |  |  |  |

Q4.10 Timing

First Click (1)

Last Click (2)

Page Submit (3)

Click Count (4)

| Page Break |  |
| --- | --- |

| 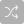 |
| --- |

Q4.11

|  | Strongly agree (1) | Agree (2) | Somewhat agree (3) | Neither agree nor disagree (4) | Somewhat disagree (5) | Disagree (6) | Strongly disagree (7) |
| --- | --- | --- | --- | --- | --- | --- | --- |
| If interrupted, I looked forward to returning to the activity (1) |  |  |  |  |  |  |  |
| Everyday thoughts and concerns faded out during the activity (2) |  |  |  |  |  |  |  |
| I was more focused on the activity rather on any external distraction (8) |  |  |  |  |  |  |  |

Q4.12 Timing

First Click (1)

Last Click (2)

Page Submit (3)

Click Count (4)

| Page Break |  |
| --- | --- |

| 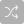 |
| --- |

Q4.13

|  | Strongly agree (1) | Agree (2) | Somewhat agree (3) | Neither agree nor disagree (4) | Somewhat disagree (5) | Disagree (6) | Strongly disagree (7) |
| --- | --- | --- | --- | --- | --- | --- | --- |
| The activity felt so authentic that it made me think that the virtual characters/objects existed for real (1) |  |  |  |  |  |  |  |
| I felt that what I was experiencing was something real, instead of a fictional activity (2) |  |  |  |  |  |  |  |
| I was so involved in the activity, that in some cases I wanted to interact with the virtual characters/objects directly (8) |  |  |  |  |  |  |  |
| I so was involved, that I felt that my actions could affect the activity (10) |  |  |  |  |  |  |  |

Q4.14 Timing

First Click (1)

Last Click (2)

Page Submit (3)

Click Count (4)

| Page Break |  |
| --- | --- |

| 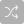 |
| --- |

Q4.15

|  | Strongly agree (1) | Agree (2) | Somewhat agree (3) | Neither agree nor disagree (4) | Somewhat disagree (5) | Disagree (6) | Strongly disagree (7) |
| --- | --- | --- | --- | --- | --- | --- | --- |
| I didn’t have any irrelevant thoughts or external distractions during the activity (1) |  |  |  |  |  |  |  |
| The activity became the unique and only thought occupying my mind (2) |  |  |  |  |  |  |  |
| I lost track of time, as if everything just stopped, and the only thing that I could think about was the activity (8) |  |  |  |  |  |  |  |

Q4.16 Timing

First Click (1)

Last Click (2)

Page Submit (3)

Click Count (4)

End of Block: Augmented Reality Immersion

Start of Block: CEI-II

Q5.1 Please answer the following questions about yourself quickly. There is no "right" or "wrong" answer.

|  | very slightly or not at all (1) | a little (2) | moderately (3) | quite a bit (4) | extremely (5) |
| --- | --- | --- | --- | --- | --- |
| I actively seek as much information as I can in new situations. (1) |  |  |  |  |  |
| I am the type of person who really enjoys the uncertainty of everyday life. (6) |  |  |  |  |  |
| I am at my best when doing something that is complex or challenging. (4) |  |  |  |  |  |
| Everywhere I go, I am out looking for new things or experiences. (5) |  |  |  |  |  |
| I view challenging situations as an opportunity to grow and learn. (2) |  |  |  |  |  |
| I like to do things that are a little frightening. (3) |  |  |  |  |  |
| I am always looking for experiences that challenge how I think about myself and the world. (7) |  |  |  |  |  |
| I prefer jobs that are excitingly unpredictable. (8) |  |  |  |  |  |
| I frequently seek out opportunities to challenge myself and grow as a person. (9) |  |  |  |  |  |
| I am the kind of person who embraces unfamiliar people, events, and places. (10) |  |  |  |  |  |

Q5.2 Timing

First Click (1)

Last Click (2)

Page Submit (3)

Click Count (4)

End of Block: CEI-II

Start of Block: Recall

Q6.1 In the following, we will ask you a couple of questions on your understanding of the biological processes that were presented in the game. There is always only one correct response.

| Page Break |  |
| --- | --- |

| 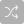 |
| --- |

Q6.2 The osteoclast binds to proteins on the bone surface via which molecular sequence?

- RER-sequence (1)
- RGD-sequence (2)
- PAL- sequence (3)
- RED-sequence (4)

| 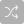 |
| --- |

Q6.3 Which cells fuse to form a mature osteoclast?

- erythrocytes (1)
- osteoblasts (2)
- hematopoetic progenitor cells (3)
- lymphoid progenitor cells (4)

| 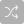 |
| --- |

Q6.4 Which cell type resorbs bone?

- osteocyte (1)
- osteoblast (2)
- osteoclast (3)
- osteoid (4)

| 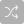 |
| --- |

Q6.5 Which acid is responsible for dissolving calcium and phosphorus?

- hydrochloric acid (1)
- hydrofluoric acid (2)
- nitric acid (3)
- citric acid (4)

| 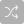 |
| --- |

Q6.6 Which cells form new bone?

- osteoclasts (1)
- osteocytes (2)
- polymorphonuclear cells (3)
- osteoblasts (4)

Q6.7 Mesenchymal osteogenic progenitor (MOP) cells differentiate into which cell type?

- osteoblasts (1)
- osteoclasts (2)
- mesenchymal stem cells (3)
- mesenchymal stromal cells (4)

Q6.8 Timing

First Click (1)

Last Click (2)

Page Submit (3)

Click Count (4)

End of Block: Recall

Start of Block: Transfer

Q7.1 In the following, we will ask you a few questions that build on the content presented in the game. There is always only one correct answer.

| Page Break |  |
| --- | --- |

| 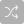 |
| --- |

Q7.2 Which cell type signals the need for bone remodelling?

- osteoblast (1)
- osteoclast (2)
- osteocyte (3)
- mesenchymal stem cell (4)

| 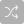 |
| --- |

Q7.3 What is the reason for the “sealing zone”?

- protected zone for acid mediated bone resorption (1)
- protection against invading cells (2)
- barrier between spongious and cortical bone (3)
- barrier between blood vessels and bone (4)

| 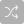 |
| --- |

Q7.4 Acid and enzymes dissolve hard and soft tissue components under the canopy. Where do these dissolved components find themselves later?

- in lymphatic tissue (1)
- in the bloodstream (2)
- nowhere (3)
- in bile fluid (4)

| 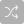 |
| --- |

Q7.5 What is the role of bone morphogenetic proteins (BMPs)?

- induction of bone resorption (1)
- dissolving hard tissues (2)
- induction of osteogenic differentiation (3)
- formation of the canopy (4)

| 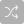 |
| --- |

Q7.6 Which is the correct order of the following procedures of bone remodelling?

- bone resorption – MOP cells – bone formation (1)
- bone formation – MOP cells – bone resorption (2)
- bone resorption – sealing zone formation – bone formation (3)
- bone formation – sealing zone formation – bone formation (4)

Q7.7 Timing

First Click (1)

Last Click (2)

Page Submit (3)

Click Count (4)

End of Block: Transfer

Start of Block: Visual Transfer

Q8.1 In the following, we will show you three images that show some content related to osteoclasts. Please select the answer that describes the content of the image best. There is only one correct answer.

Q8.2 Timing

First Click (1)

Last Click (2)

Page Submit (3)

Click Count (4)

| Page Break |  |
| --- | --- |

Q8.3 Please look at this scene involving Osteoclasts and answer the following question.

| 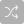 |
| --- |

Q8.4 Which cell type coming from the blood becomes the osteoclast?

- Endothelial cell (1)
- Bone Lining Cell (2)
- Hematopoetic stem cell (5)
- Macrophage (6)

Q8.5 Timing

First Click (1)

Last Click (2)

Page Submit (3)

Click Count (4)

| Page Break |  |
| --- | --- |

Q8.6 Please look at this scene involving Osteoclasts and answer the following question.

Q8.7 The osteoclast has now started to resorb bone. What factors are released by this bone resorption?

- Calcium and Phosphor (1)
- Calcium and TGF-B (2)
- Phosphor and TGF-B (3)
- Collagen and TGF-B (4)

Q8.8 Timing

First Click (1)

Last Click (2)

Page Submit (3)

Click Count (4)

| Page Break |  |
| --- | --- |

Q8.9 Please look at this scene involving Osteoclasts and answer the following question.

| 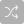 |
| --- |

Q8.10 Which cell attaches to the resorption lacuna created by osteoclasts?

- Osteoblast (1)
- Mesenchymal stem cell (2)
- Mesenchymale Osteoprogenitor cell (3)
- None of the above (4)

Q8.11 Timing

First Click (1)

Last Click (2)

Page Submit (3)

Click Count (4)

End of Block: Visual Transfer

Start of Block: Qualitative answers

Q9.1 We will now ask you to answer three questions on the content you learned while playing. Please keep your answers short and write only between 3 to 6 sentences.

Q9.2 Timing

First Click (1)

Last Click (2)

Page Submit (3)

Click Count (4)

| Page Break |  |
| --- | --- |

Q9.3 Describe the formation of an osteoclast in 3 to 6 sentences.

________________________________________________________________

________________________________________________________________

________________________________________________________________

________________________________________________________________

________________________________________________________________

Q9.4 Timing

First Click (1)

Last Click (2)

Page Submit (3)

Click Count (4)

| Page Break |  |
| --- | --- |

Q9.5 Describe in words the function of osteoclasts and osteoblasts in the canopy in 3 to 6 sentences.

________________________________________________________________

________________________________________________________________

________________________________________________________________

________________________________________________________________

________________________________________________________________

Q9.6 Timing

First Click (1)

Last Click (2)

Page Submit (3)

Click Count (4)

| Page Break |  |
| --- | --- |

Q9.7 Describe the processes and molecules involved in osteogenic differentiation, and the role they play in 3 to 6 sentences.

________________________________________________________________

________________________________________________________________

________________________________________________________________

________________________________________________________________

________________________________________________________________

Q9.8 Timing

First Click (1)

Last Click (2)

Page Submit (3)

Click Count (4)

Q9.9 Timing

First Click (1)

Last Click (2)

Page Submit (3)

Click Count (4)

End of Block: Qualitative answers

Start of Block: IMMS

Q10.1 In the following, we present you with several statement and ask you to identify how much you agree or disagree with them. There is no "right" or "wrong". Please answer truthfully to help us evaluate the Augmented Reality Game.

Q10.3 When I first looked at this lesson, I had the impression that it would be easy for me.

- Agree (1)
- Somewhat agree (2)
- Neither agree nor disagree (3)
- Somewhat disagree (4)
- Disagree (5)

Q10.5 This material was more difficult to understand than I would like for it to be.

- Agree (1)
- Somewhat agree (2)
- Neither agree nor disagree (3)
- Somewhat disagree (4)
- Disagree (5)

Q10.7 After reading the introductory information, I felt confident that I knew what I was supposed to learn from this lesson.

- Agree (1)
- Somewhat agree (2)
- Neither agree nor disagree (3)
- Somewhat disagree (4)
- Disagree (5)

Q10.9 Many of the pages had so much information that it was hard to pick out and remember the important points.

- Agree (1)
- Somewhat agree (2)
- Neither agree nor disagree (3)
- Somewhat disagree (4)
- Disagree (5)

Q10.11 As I worked on this lesson, I was confident that I could learn the content.

- Agree (1)
- Somewhat agree (2)
- Neither agree nor disagree (3)
- Somewhat disagree (4)
- Disagree (5)

Q10.13 The exercises in this lesson were too difficult.

- Agree (1)
- Somewhat agree (2)
- Neither agree nor disagree (3)
- Somewhat disagree (4)
- Disagree (5)

Q10.15 After working on this lesson for a while, I was confident that I would be able to pass a test on it.

- Agree (1)
- Somewhat agree (2)
- Neither agree nor disagree (3)
- Somewhat disagree (4)
- Disagree (5)

Q10.17 I could not really understand quite a bit of the material in this lesson.

- Agree (1)
- Somewhat agree (2)
- Neither agree nor disagree (3)
- Somewhat disagree (4)
- Disagree (5)

Q10.19 The good organization of the content helped me be confident that I would learn this material.

- Agree (1)
- Somewhat agree (2)
- Neither agree nor disagree (3)
- Somewhat disagree (4)
- Disagree (5)

Q10.21 When I first looked at this lesson, I had the impression that it would be easy for me.

- Agree (1)
- Somewhat agree (2)
- Neither agree nor disagree (3)
- Somewhat disagree (4)
- Disagree (5)

Q10.23 These materials are eye-catching.

- Agree (1)
- Somewhat agree (2)
- Neither agree nor disagree (3)
- Somewhat disagree (4)
- Disagree (5)

Q10.25 The quality of the writing helped to hold my attention.

- Agree (1)
- Somewhat agree (2)
- Neither agree nor disagree (3)
- Somewhat disagree (4)
- Disagree (5)

Q10.27 This lesson is so abstract that it was hard to keep my attention on it.

- Agree (1)
- Somewhat agree (2)
- Neither agree nor disagree (3)
- Somewhat disagree (4)
- Disagree (5)

Q10.29 The pages of this lesson look dry and unappealing.

- Agree (19)
- Somewhat agree (20)
- Neither agree nor disagree (21)
- Somewhat disagree (22)
- Disagree (23)

Q10.31 The way the information is arranged on the pages helped keep my attention.

- Agree (1)
- Somewhat agree (2)
- Neither agree nor disagree (3)
- Somewhat disagree (4)
- Disagree (5)

Q10.33 This lesson has things that stimulated my curiosity.

- Agree (1)
- Somewhat agree (2)
- Neither agree nor disagree (3)
- Somewhat disagree (4)
- Disagree (5)

Q10.35 The amount of repetition in this lesson caused me to get bored sometimes.

- Agree (1)
- Somewhat agree (2)
- Neither agree nor disagree (3)
- Somewhat disagree (4)
- Disagree (5)

Q10.37 I learned some things that were surprising or unexpected.

- Agree (1)
- Somewhat agree (2)
- Neither agree nor disagree (3)
- Somewhat disagree (4)
- Disagree (5)

Q10.39 The variety of reading passages, exercises, illustrations, etc., helped keep my attention on the lesson.

- Agree (1)
- Somewhat agree (2)
- Neither agree nor disagree (3)
- Somewhat disagree (4)
- Disagree (5)

Q10.41 There are so many words on each page that it is irritating.

- Agree (1)
- Somewhat agree (2)
- Neither agree nor disagree (3)
- Somewhat disagree (4)
- Disagree (5)

Q10.43 Completing the exercises in this lesson gave me a satisfying feeling of accomplishment.

- Agree (1)
- Somewhat agree (2)
- Neither agree nor disagree (3)
- Somewhat disagree (4)
- Disagree (5)

Q10.45 I enjoyed this lesson so much that I would like to know more about this topic.

- Agree (1)
- Somewhat agree (2)
- Neither agree nor disagree (3)
- Somewhat disagree (4)
- Disagree (5)

Q10.47 I really enjoyed studying this lesson.

- Agree (1)
- Somewhat agree (2)
- Neither agree nor disagree (3)
- Somewhat disagree (4)
- Disagree (5)

Q10.49 The wording of feedback after the exercises, or of other comments in this lesson, helped me feel rewarded for my effort.

- Agree (1)
- Somewhat agree (2)
- Neither agree nor disagree (3)
- Somewhat disagree (4)
- Disagree (5)

Q10.51 It felt good to successfully complete this lesson.

- Agree (1)
- Somewhat agree (2)
- Neither agree nor disagree (3)
- Somewhat disagree (4)
- Disagree (5)

Q10.53 It was a pleasure to work on such a well-designed lesson.

- Agree (1)
- Somewhat agree (2)
- Neither agree nor disagree (3)
- Somewhat disagree (4)
- Disagree (5)

Q10.55 It is clear to me how the content of this material is related to things I already know.

- Agree (1)
- Somewhat agree (2)
- Neither agree nor disagree (3)
- Somewhat disagree (4)
- Disagree (5)

Q10.57 There were stories, pictures, or examples that showed me how this material could be important to some people.

- Agree (1)
- Somewhat agree (2)
- Neither agree nor disagree (3)
- Somewhat disagree (4)
- Disagree (5)

Q10.59 Completing this lesson successfully was important to me.

- Agree (1)
- Somewhat agree (2)
- Neither agree nor disagree (3)
- Somewhat disagree (4)
- Disagree (5)

Q10.61 The content of this material is relevant to my interests.

- Agree (1)
- Somewhat agree (2)
- Neither agree nor disagree (3)
- Somewhat disagree (4)
- Disagree (5)

Q10.63 There are explanations or examples of how people use the knowledge in this lesson.

- Agree (1)
- Somewhat agree (2)
- Neither agree nor disagree (3)
- Somewhat disagree (4)
- Disagree (5)

Q10.65 The content and style of writing in this lesson convey the impression that its content is worth knowing.

- Agree (1)
- Somewhat agree (2)
- Neither agree nor disagree (3)
- Somewhat disagree (4)
- Disagree (5)

Q10.67 This lesson was not relevant to my needs because I already knew most of it.

- Agree (1)
- Somewhat agree (2)
- Neither agree nor disagree (3)
- Somewhat disagree (4)
- Disagree (5)

Q10.69 I could relate the content of this lesson to things I have seen, done, or thought about in my own life.

- Agree (1)
- Somewhat agree (2)
- Neither agree nor disagree (3)
- Somewhat disagree (4)
- Disagree (5)

Q10.71 The content of this lesson will be useful to me.

- Agree (1)
- Somewhat agree (2)
- Neither agree nor disagree (3)
- Somewhat disagree (4)
- Disagree (5)

Q10.72 Timing

First Click (1)

Last Click (2)

Page Submit (3)

Click Count (4)

End of Block: IMMS

Start of Block: Follow-up

Q11.1
Thank you for participating in this questionnaire!

Q11.2
We would like to conduct a follow-up survey in two weeks to understand how much about osteoclasts you still remember after playing the game.


If you are willing to participate, please provide an email address we can use to contact you. The email address will not be shared with third parties and will not be used to identify you beyond this series of surveys. The email address will only be used to send you an invitation to the follow-up survey and connect your answers from the first survey with the follow-up survey.

- No, I don't want to participate in a follow-up. (1)
- Yes, I want to participate in a follow-up. You can contact me under this email (2) ________________________________________________

Q11.3 Timing

First Click (1)

Last Click (2)

Page Submit (3)

Click Count (4)

End of Block: Follow-up

AR Osteoclasts Feedback - 1 week

Survey Flow

Block: Consent (6 Questions)

Standard: Identifier (3 Questions)

Branch: New Branch

If

If Did you play the Augmented Reality Educational Game "AR Osteoclasts"? Yes Is Selected

Standard: Recall (8 Questions)

Standard: Transfer (7 Questions)

Standard: Visual Transfer (11 Questions)

Standard: Qualitative answers (9 Questions)

Standard: Follow-up (3 Questions)

| Page Break |  |
| --- | --- |

Start of Block: Consent

Q1.1
Thank you for participating in this user study for our educational augmented reality game AR Osteoclasts. We thank you for joining us again one week later. Your answers help us to determine the effectiveness of our educational game.


Before we start with the test, we would like to get your consent to use your data for scientific evaluation.

Q1.2 Can we use your answers to this survey for our analysis?

- Yes (1)
- No (2)

Q1.3 Are you older than 18 years?

- Yes (1)
- No (2)

Q1.4 Timing

First Click (1)

Last Click (2)

Page Submit (3)

Click Count (4)

| Page Break |  |
| --- | --- |

Q1.6 Did you play the Augmented Reality Educational Game "AR Osteoclasts"?

- Yes (1)
- No (2)

Q1.8 Timing

First Click (1)

Last Click (2)

Page Submit (3)

Click Count (4)

End of Block: Consent

Start of Block: Identifier

| 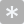 |
| --- |

Q2.1
Please write down your participant id that you generated on the piece of paper before the user study. Hopefully, you took a picture of the code. If not, here are the instructions again:
 
Example: my youngest parent is called Katia (▲), my oldest parent is born in 1975 (⚫), and my best friend is called Anna (◾), so my participant ID is: KA75AN (▲ ⚫ ◾)
 
To compute your own participant id, write: The first two letters for the name of your youngest parent __________ (▲) The last two digits of the birth year of your oldest parent __________ (⚫) The first two letters of your best friend’s name __________ (◾) Write here your final participant id in the text box below.

 Please note that a few of you had KA75AN. If your id was KA75AN, please add the number we assigned you during the in person session to your code.

________________________________________________________________

Q164 Because some of you had the same participant id, we would like you to repeat with what gender do you identify?

- Male (1)
- Female (3)
- Non-binary / third gender (4)
- Prefer not to say (5)

Q2.2 Timing

First Click (1)

Last Click (2)

Page Submit (3)

Click Count (4)

End of Block: Identifier

Start of Block: Recall

Q6.1 In the following, we will ask you a couple of questions on your understanding of the biological processes that were presented in the game. There is always only one correct response.

| Page Break |  |
| --- | --- |

| 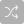 |
| --- |

Q6.2 The osteoclast binds to proteins on the bone surface via which molecular sequence?

- RER-sequence (1)
- RGD-sequence (2)
- PAL- sequence (3)
- RED-sequence (4)

| 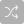 |
| --- |

Q6.3 Which cells fuse to form a mature osteoclast?

- erythrocytes (1)
- osteoblasts (2)
- hematopoetic progenitor cells (3)
- lymphoid progenitor cells (4)

| 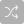 |
| --- |

Q6.4 Which cell type resorbs bone?

- osteocyte (1)
- osteoblast (2)
- osteoclast (3)
- osteoid (4)

| 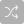 |
| --- |

Q6.5 Which acid is responsible for dissolving calcium and phosphorus?

- hydrochloric acid (1)
- hydrofluoric acid (2)
- nitric acid (3)
- citric acid (4)

| 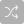 |
| --- |

Q6.6 Which cells form new bone?

- osteoclasts (1)
- osteocytes (2)
- polymorphonuclear cells (3)
- osteoblasts (4)

Q6.7 Mesenchymal osteogenic progenitor (MOP) cells differentiate into which cell type?

- osteoblasts (1)
- osteoclasts (2)
- mesenchymal stem cells (3)
- mesenchymal stromal cells (4)

Q6.8 Timing

First Click (1)

Last Click (2)

Page Submit (3)

Click Count (4)

End of Block: Recall

Start of Block: Transfer

Q7.1 In the following, we will ask you a few questions that build on the content presented in the game. There is always only one correct answer.

| Page Break |  |
| --- | --- |

| 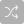 |
| --- |

Q7.2 Which cell type signals the need for bone remodelling?

- osteoblast (1)
- osteoclast (2)
- osteocyte (3)
- mesenchymal stem cell (4)

| 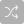 |
| --- |

Q7.3 What is the reason for the “sealing zone”?

- protected zone for acid mediated bone resorption (1)
- protection against invading cells (2)
- barrier between spongious and cortical bone (3)
- barrier between blood vessels and bone (4)

| 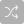 |
| --- |

Q7.4 Acid and enzymes dissolve hard and soft tissue components under the canopy. Where do these dissolved components find themselves later?

- in lymphatic tissue (1)
- in the bloodstream (2)
- nowhere (3)
- in bile fluid (4)

| 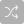 |
| --- |

Q7.5 What is the role of bone morphogenetic proteins (BMPs)?

- induction of bone resorption (1)
- dissolving hard tissues (2)
- induction of osteogenic differentiation (3)
- formation of the canopy (4)

| 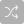 |
| --- |

Q7.6 Which is the correct order of the following procedures of bone remodelling?

- bone resorption – MOP cells – bone formation (1)
- bone formation – MOP cells – bone resorption (2)
- bone resorption – sealing zone formation – bone formation (3)
- bone formation – sealing zone formation – bone formation (4)

Q7.7 Timing

First Click (1)

Last Click (2)

Page Submit (3)

Click Count (4)

End of Block: Transfer

Start of Block: Visual Transfer

Q8.1 In the following, we will show you three images that show some content related to osteoclasts. Please select the answer that describes the content of the image best. There is only one correct answer.

Q8.2 Timing

First Click (1)

Last Click (2)

Page Submit (3)

Click Count (4)

| Page Break |  |
| --- | --- |

Q8.3 Please look at this scene involving Osteoclasts and answer the following question.

| 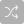 |
| --- |

Q8.4 Which cell type coming from the blood becomes the osteoclast?

- Endothelial cell (1)
- Bone Lining Cell (2)
- Hematopoetic stem cell (5)
- Macrophage (6)

Q8.5 Timing

First Click (1)

Last Click (2)

Page Submit (3)

Click Count (4)

| Page Break |  |
| --- | --- |

Q8.6 Please look at this scene involving Osteoclasts and answer the following question.

Q8.7 The osteoclast has now started to resorb bone. What factors are released by this bone resorption?

- Calcium and Phosphor (1)
- Calcium and TGF-B (2)
- Phosphor and TGF-B (3)
- Collagen and TGF-B (4)

Q8.8 Timing

First Click (1)

Last Click (2)

Page Submit (3)

Click Count (4)

| Page Break |  |
| --- | --- |

Q8.9 Please look at this scene involving Osteoclasts and answer the following question.

| 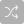 |
| --- |

Q8.10 Which cell attaches to the resorption lacuna created by osteoclasts?

- Osteoblast (1)
- Mesenchymal stem cell (2)
- Mesenchymale Osteoprogenitor cell (3)
- None of the above (4)

Q8.11 Timing

First Click (1)

Last Click (2)

Page Submit (3)

Click Count (4)

End of Block: Visual Transfer

Start of Block: Qualitative answers

Q9.1 We will now ask you to answer three questions on the content you learned while playing. Please keep your answers short and write only between 3 to 6 sentences.

Q9.2 Timing

First Click (1)

Last Click (2)

Page Submit (3)

Click Count (4)

| Page Break |  |
| --- | --- |

Q9.3 Describe the formation of an osteoclast in 3 to 6 sentences.

________________________________________________________________

________________________________________________________________

________________________________________________________________

________________________________________________________________

________________________________________________________________

Q9.4 Timing

First Click (1)

Last Click (2)

Page Submit (3)

Click Count (4)

| Page Break |  |
| --- | --- |

Q9.5 Describe in words the function of osteoclasts and osteoblasts in the canopy in 3 to 6 sentences.

________________________________________________________________

________________________________________________________________

________________________________________________________________

________________________________________________________________

________________________________________________________________

Q9.6 Timing

First Click (1)

Last Click (2)

Page Submit (3)

Click Count (4)

| Page Break |  |
| --- | --- |

Q9.7 Describe the processes and molecules involved in osteogenic differentiation, and the role they play in 3 to 6 sentences.

________________________________________________________________

________________________________________________________________

________________________________________________________________

________________________________________________________________

________________________________________________________________

Q9.8 Timing

First Click (1)

Last Click (2)

Page Submit (3)

Click Count (4)

Q9.9 Timing

First Click (1)

Last Click (2)

Page Submit (3)

Click Count (4)

End of Block: Qualitative answers

Start of Block: Follow-up

Q11.1
Thank you for participating in this questionnaire!

Q11.2
We would like to conduct a follow-up survey one month after the first session to understand how much about osteoclasts you still remember after playing the game.


If you are willing to participate, please provide an email address we can use to contact you. The email address will not be shared with third parties and will not be used to identify you beyond this series of surveys. The email address will only be used to send you an invitation to the follow-up survey and connect your answers from the first survey with the follow-up survey.

- No, I don't want to participate in a follow-up. (1)
- Yes, I want to participate in a follow-up. You can contact me under this email (2) __________________________________________________

Q11.3 Timing

First Click (1)

Last Click (2)

Page Submit (3)

Click Count (4)

End of Block: Follow-up

AR Osteoclasts Feedback - 1 month

Survey Flow

Block: Consent (6 Questions)

Standard: Identifier (3 Questions)

Branch: New Branch

If

If Did you play the Augmented Reality Educational Game "AR Osteoclasts"? Yes Is Selected

Standard: Recall (8 Questions)

Standard: Transfer (7 Questions)

Standard: Visual Transfer (11 Questions)

Standard: Qualitative answers (9 Questions)

Standard: Follow-up (2 Questions)

| Page Break |  |
| --- | --- |

Start of Block: Consent

Q1.1
Thank you for participating in this user study for our educational augmented reality game AR Osteoclasts. We thank you for joining us again one month later. Your answers help us to determine the effectiveness of our educational game.


Before we start with the test, we would like to get your consent to use your data for scientific evaluation.

Q1.2 Can we use your answers to this survey for our analysis?

- Yes (1)
- No (2)

Q1.3 Are you older than 18 years?

- Yes (1)
- No (2)

Q1.4 Timing

First Click (1)

Last Click (2)

Page Submit (3)

Click Count (4)

| Page Break |  |
| --- | --- |

Q1.6 Did you play the Augmented Reality Educational Game "AR Osteoclasts"?

- Yes (1)
- No (2)

Q1.8 Timing

First Click (1)

Last Click (2)

Page Submit (3)

Click Count (4)

End of Block: Consent

Start of Block: Identifier

| 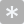 |
| --- |

Q2.1
Please write down your participant id that you generated on the piece of paper before the user study. Hopefully, you took a picture of the code. If not, here are the instructions again:
 
Example: my youngest parent is called Katia (▲), my oldest parent is born in 1975 (⚫), and my best friend is called Anna (◾), so my participant ID is: KA75AN (▲ ⚫ ◾)
 
To compute your own participant id, write: The first two letters for the name of your youngest parent __________ (▲) The last two digits of the birth year of your oldest parent __________ (⚫) The first two letters of your best friend’s name __________ (◾) Write here your final participant id in the text box below.

 Please note that a few of you had KA75AN. If your id was KA75AN, please add the number we assigned you during the in person session to your code.

________________________________________________________________

Q164 Because some of you had the same participant id, we would like you to repeat with what gender do you identify?

- Male (1)
- Female (3)
- Non-binary / third gender (4)
- Prefer not to say (5)

Q2.2 Timing

First Click (1)

Last Click (2)

Page Submit (3)

Click Count (4)

End of Block: Identifier

Start of Block: Recall

Q6.1 In the following, we will ask you a couple of questions on your understanding of the biological processes that were presented in the game. There is always only one correct response.

| Page Break |  |
| --- | --- |

| 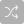 |
| --- |

Q6.2 The osteoclast binds to proteins on the bone surface via which molecular sequence?

- RER-sequence (1)
- RGD-sequence (2)
- PAL- sequence (3)
- RED-sequence (4)

| 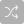 |
| --- |

Q6.3 Which cells fuse to form a mature osteoclast?

- erythrocytes (1)
- osteoblasts (2)
- hematopoetic progenitor cells (3)
- lymphoid progenitor cells (4)

| 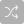 |
| --- |

Q6.4 Which cell type resorbs bone?

- osteocyte (1)
- osteoblast (2)
- osteoclast (3)
- osteoid (4)

| 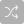 |
| --- |

Q6.5 Which acid is responsible for dissolving calcium and phosphorus?

- hydrochloric acid (1)
- hydrofluoric acid (2)
- nitric acid (3)
- citric acid (4)

| 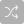 |
| --- |

Q6.6 Which cells form new bone?

- osteoclasts (1)
- osteocytes (2)
- polymorphonuclear cells (3)
- osteoblasts (4)

Q6.7 Mesenchymal osteogenic progenitor (MOP) cells differentiate into which cell type?

- osteoblasts (1)
- osteoclasts (2)
- mesenchymal stem cells (3)
- mesenchymal stromal cells (4)

Q6.8 Timing

First Click (1)

Last Click (2)

Page Submit (3)

Click Count (4)

End of Block: Recall

Start of Block: Transfer

Q7.1 In the following, we will ask you a few questions that build on the content presented in the game. There is always only one correct answer.

| Page Break |  |
| --- | --- |

| 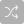 |
| --- |

Q7.2 Which cell type signals the need for bone remodelling?

- osteoblast (1)
- osteoclast (2)
- osteocyte (3)
- mesenchymal stem cell (4)

| 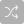 |
| --- |

Q7.3 What is the reason for the “sealing zone”?

- protected zone for acid mediated bone resorption (1)
- protection against invading cells (2)
- barrier between spongious and cortical bone (3)
- barrier between blood vessels and bone (4)

| 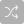 |
| --- |

Q7.4 Acid and enzymes dissolve hard and soft tissue components under the canopy. Where do these dissolved components find themselves later?

- in lymphatic tissue (1)
- in the bloodstream (2)
- nowhere (3)
- in bile fluid (4)

| 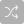 |
| --- |

Q7.5 What is the role of bone morphogenetic proteins (BMPs)?

- induction of bone resorption (1)
- dissolving hard tissues (2)
- induction of osteogenic differentiation (3)
- formation of the canopy (4)

| 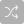 |
| --- |

Q7.6 Which is the correct order of the following procedures of bone remodelling?

- bone resorption – MOP cells – bone formation (1)
- bone formation – MOP cells – bone resorption (2)
- bone resorption – sealing zone formation – bone formation (3)
- bone formation – sealing zone formation – bone formation (4)

Q7.7 Timing

First Click (1)

Last Click (2)

Page Submit (3)

Click Count (4)

End of Block: Transfer

Start of Block: Visual Transfer

Q8.1 In the following, we will show you three images that show some content related to osteoclasts. Please select the answer that describes the content of the image best. There is only one correct answer.

Q8.2 Timing

First Click (1)

Last Click (2)

Page Submit (3)

Click Count (4)

| Page Break |  |
| --- | --- |

Q8.3 Please look at this scene involving Osteoclasts and answer the following question.

| 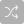 |
| --- |

Q8.4 Which cell type coming from the blood becomes the osteoclast?

- Endothelial cell (1)
- Bone Lining Cell (2)
- Hematopoetic stem cell (5)
- Macrophage (6)

Q8.5 Timing

First Click (1)

Last Click (2)

Page Submit (3)

Click Count (4)

| Page Break |  |
| --- | --- |

Q8.6 Please look at this scene involving Osteoclasts and answer the following question.

Q8.7 The osteoclast has now started to resorb bone. What factors are released by this bone resorption?

- Calcium and Phosphor (1)
- Calcium and TGF-B (2)
- Phosphor and TGF-B (3)
- Collagen and TGF-B (4)

Q8.8 Timing

First Click (1)

Last Click (2)

Page Submit (3)

Click Count (4)

| Page Break |  |
| --- | --- |

Q8.9 Please look at this scene involving Osteoclasts and answer the following question.

| 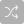 |
| --- |

Q8.10 Which cell attaches to the resorption lacuna created by osteoclasts?

- Osteoblast (1)
- Mesenchymal stem cell (2)
- Mesenchymale Osteoprogenitor cell (3)
- None of the above (4)

Q8.11 Timing

First Click (1)

Last Click (2)

Page Submit (3)

Click Count (4)

End of Block: Visual Transfer

Start of Block: Qualitative answers

Q9.1 We will now ask you to answer three questions on the content you learned while playing. Please keep your answers short and write only between 3 to 6 sentences.

Q9.2 Timing

First Click (1)

Last Click (2)

Page Submit (3)

Click Count (4)

| Page Break |  |
| --- | --- |

Q9.3 Describe the formation of an osteoclast in 3 to 6 sentences.

________________________________________________________________

________________________________________________________________

________________________________________________________________

________________________________________________________________

________________________________________________________________

Q9.4 Timing

First Click (1)

Last Click (2)

Page Submit (3)

Click Count (4)

| Page Break |  |
| --- | --- |

Q9.5 Describe in words the function of osteoclasts and osteoblasts in the canopy in 3 to 6 sentences.

________________________________________________________________

________________________________________________________________

________________________________________________________________

________________________________________________________________

________________________________________________________________

Q9.6 Timing

First Click (1)

Last Click (2)

Page Submit (3)

Click Count (4)

| Page Break |  |
| --- | --- |

Q9.7 Describe the processes and molecules involved in osteogenic differentiation, and the role they play in 3 to 6 sentences.

________________________________________________________________

________________________________________________________________

________________________________________________________________

________________________________________________________________

________________________________________________________________

Q9.8 Timing

First Click (1)

Last Click (2)

Page Submit (3)

Click Count (4)

Q9.9 Timing

First Click (1)

Last Click (2)

Page Submit (3)

Click Count (4)

End of Block: Qualitative answers

Start of Block: Follow-up

Q11.1
Thank you for participating in this questionnaire!

Q165 Timing

First Click (1)

Last Click (2)

Page Submit (3)

Click Count (4)

End of Block: Follow-up
