# Supplementary material for: Applying Augmented Reality to Convey Medical Knowledge on Osteoclasts to Users of a Serious Game: Vignette Experiment
Source: JMIR Serious Games. 2025 Jun 16;13:e64751. doi: 10.2196/64751 (PMC12185033; doi:10.2196/64751)
Supplement: Multimedia Appendix 5 — Advanced statistical analysis of the data not included in the main text. [file games-v13-e64751-s005.docx]

**Applying Augmented Reality to Convey Medical Knowledge on Osteoclasts to Users of a Serious Game: A Vignette Experiment**

## Multimedia Appendix 5: Statistical analysis

### S8 Participant responses


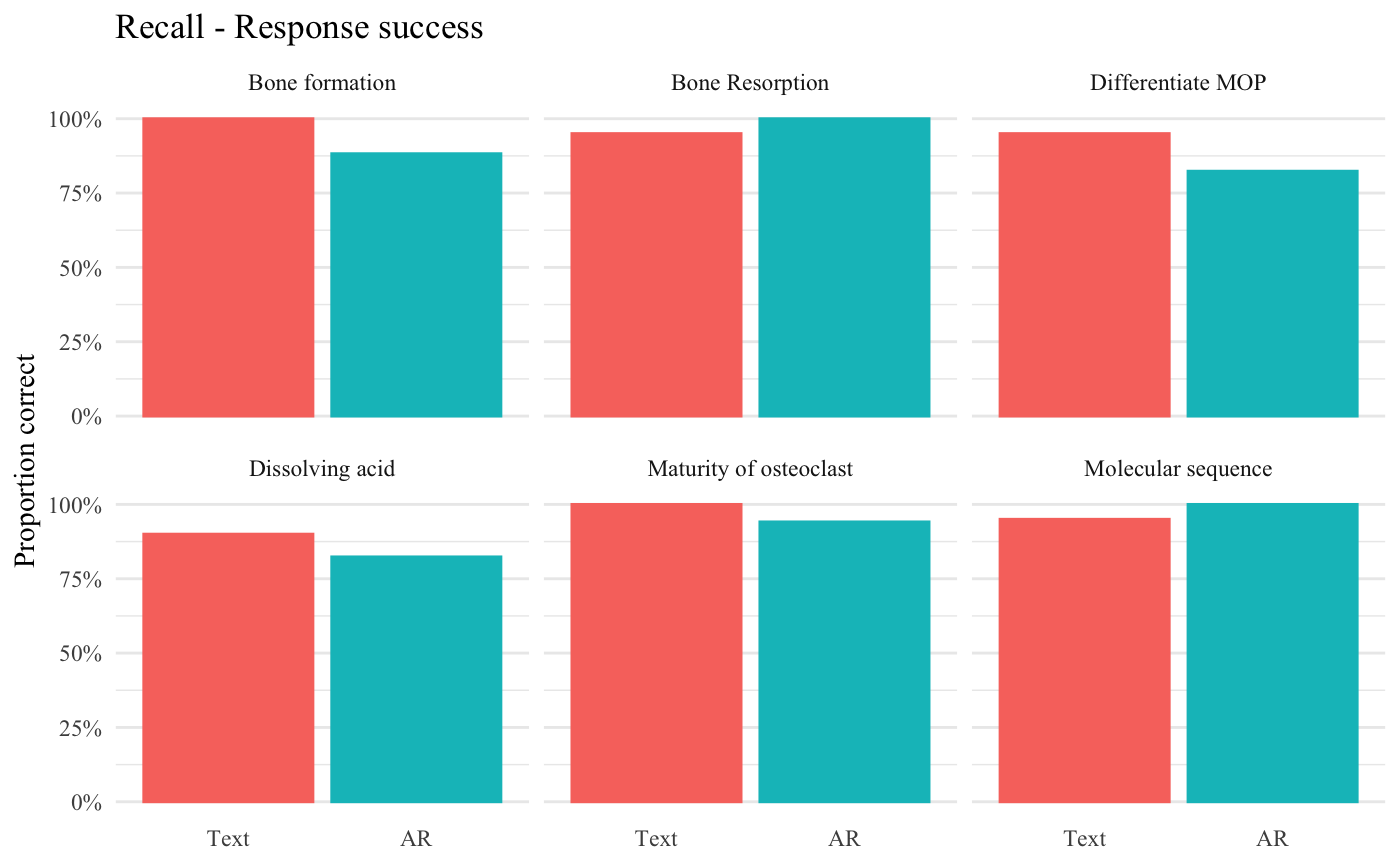


Figure S20: Outcome recall for 6 questions.


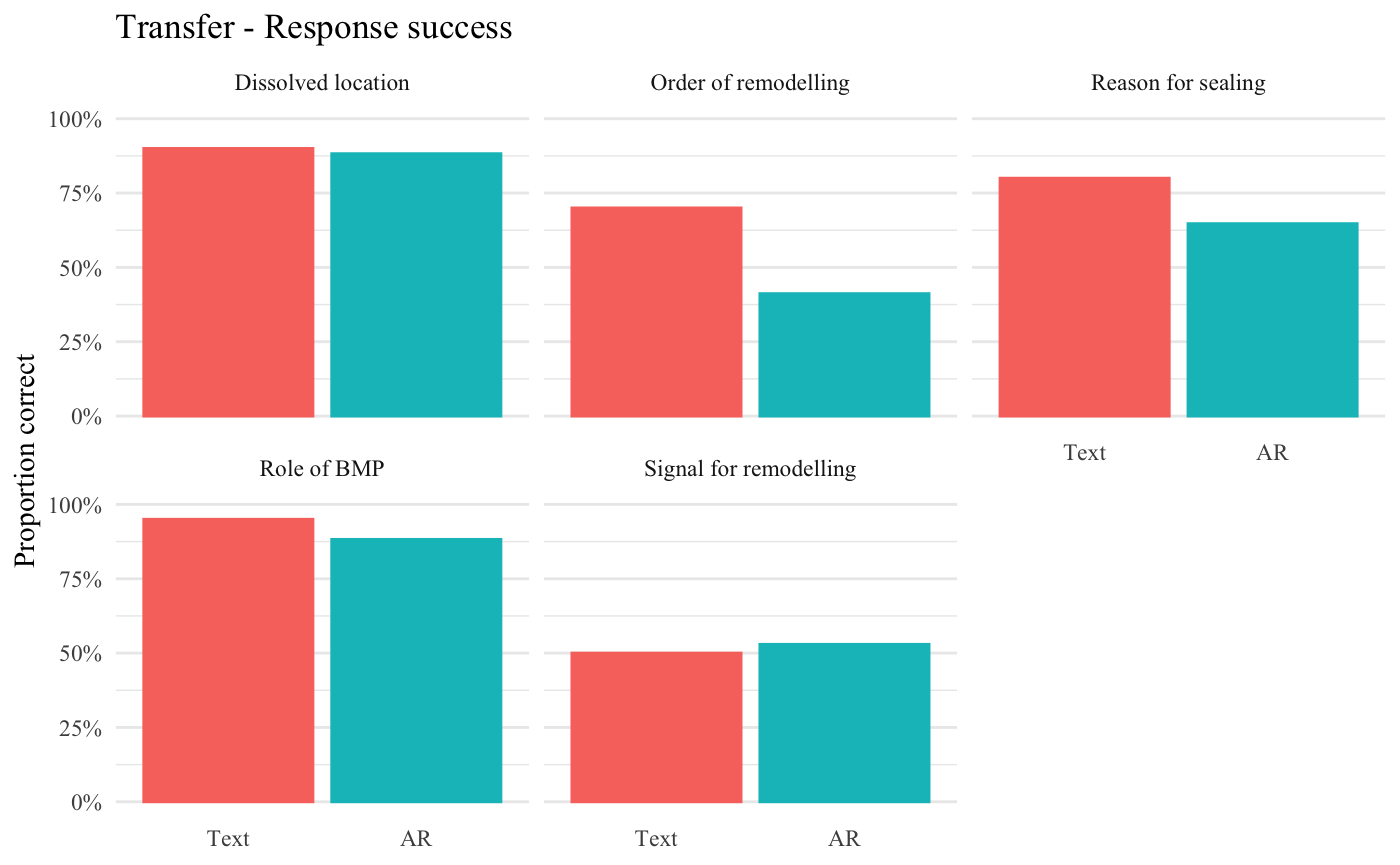


Figure S21: Outcome of Transfer for 5 questions.


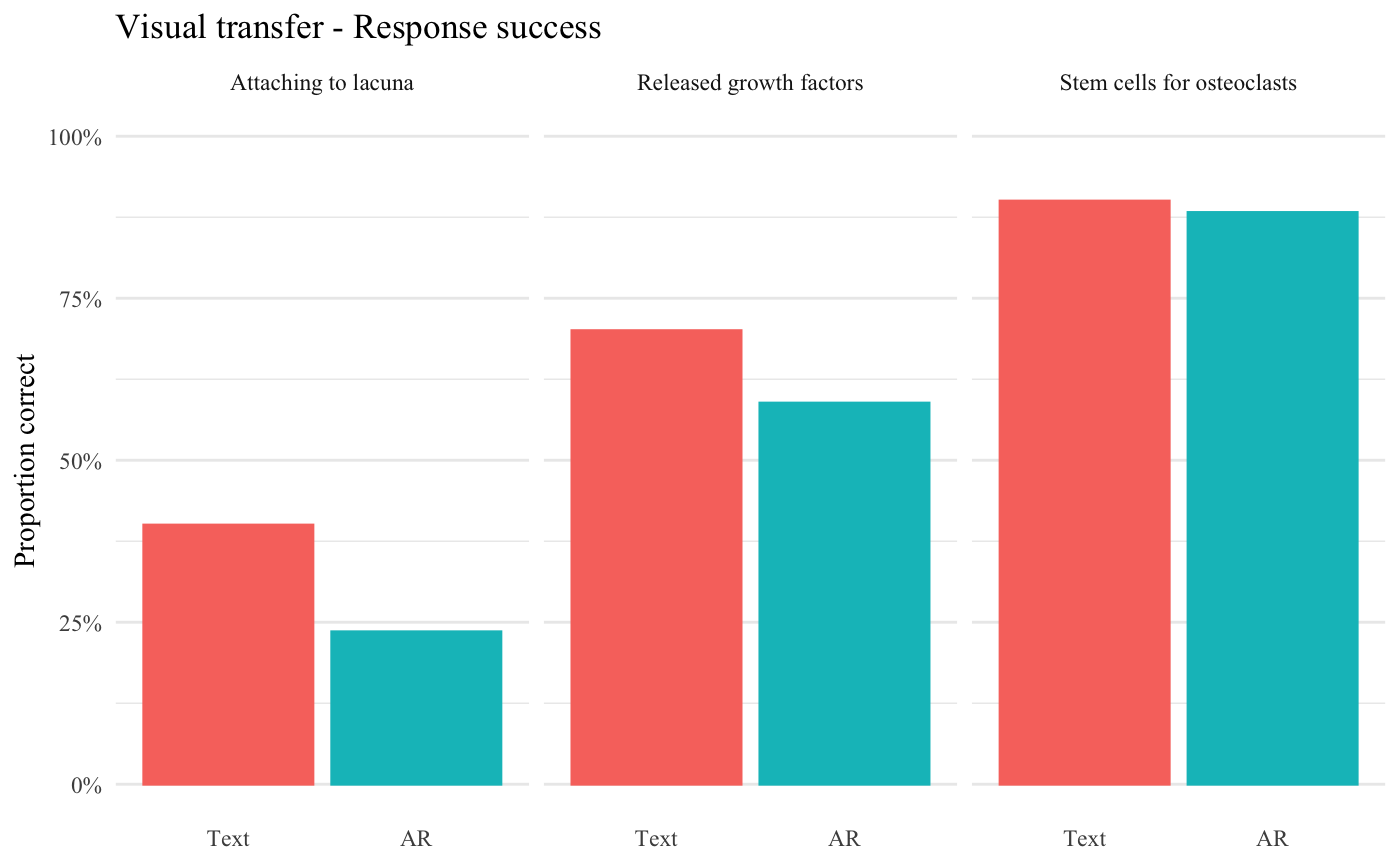


Figure S22: Outcome of Visual transfer for 3 questions.


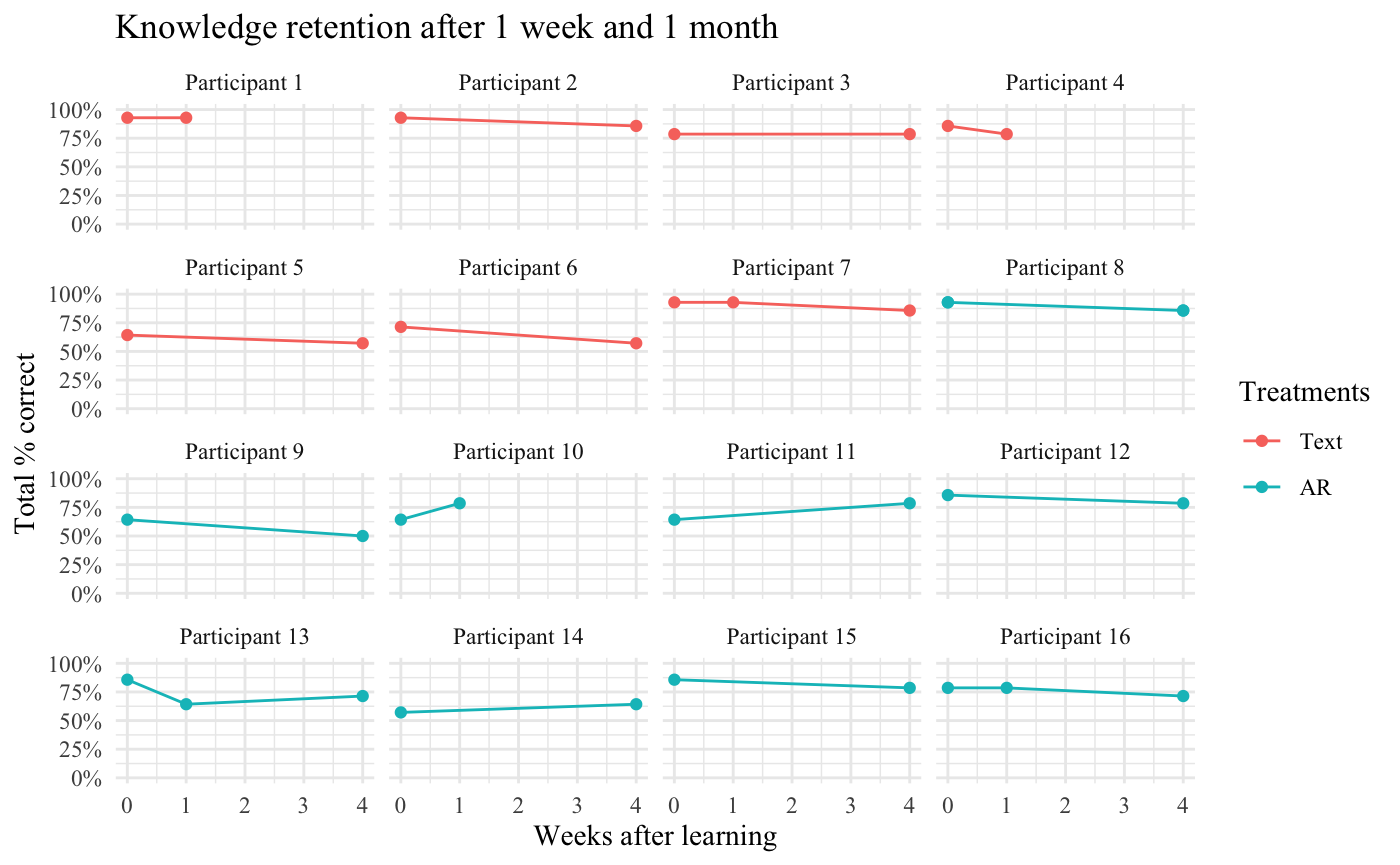


Figure S23: The evaluation score changed over the course of one month. Two measurements were taken after 1 and 4 weeks. Only a subset of participants responded. Overall, there is no significant difference between Text and AR treatment. However, a few AR participants showed improvement over time.


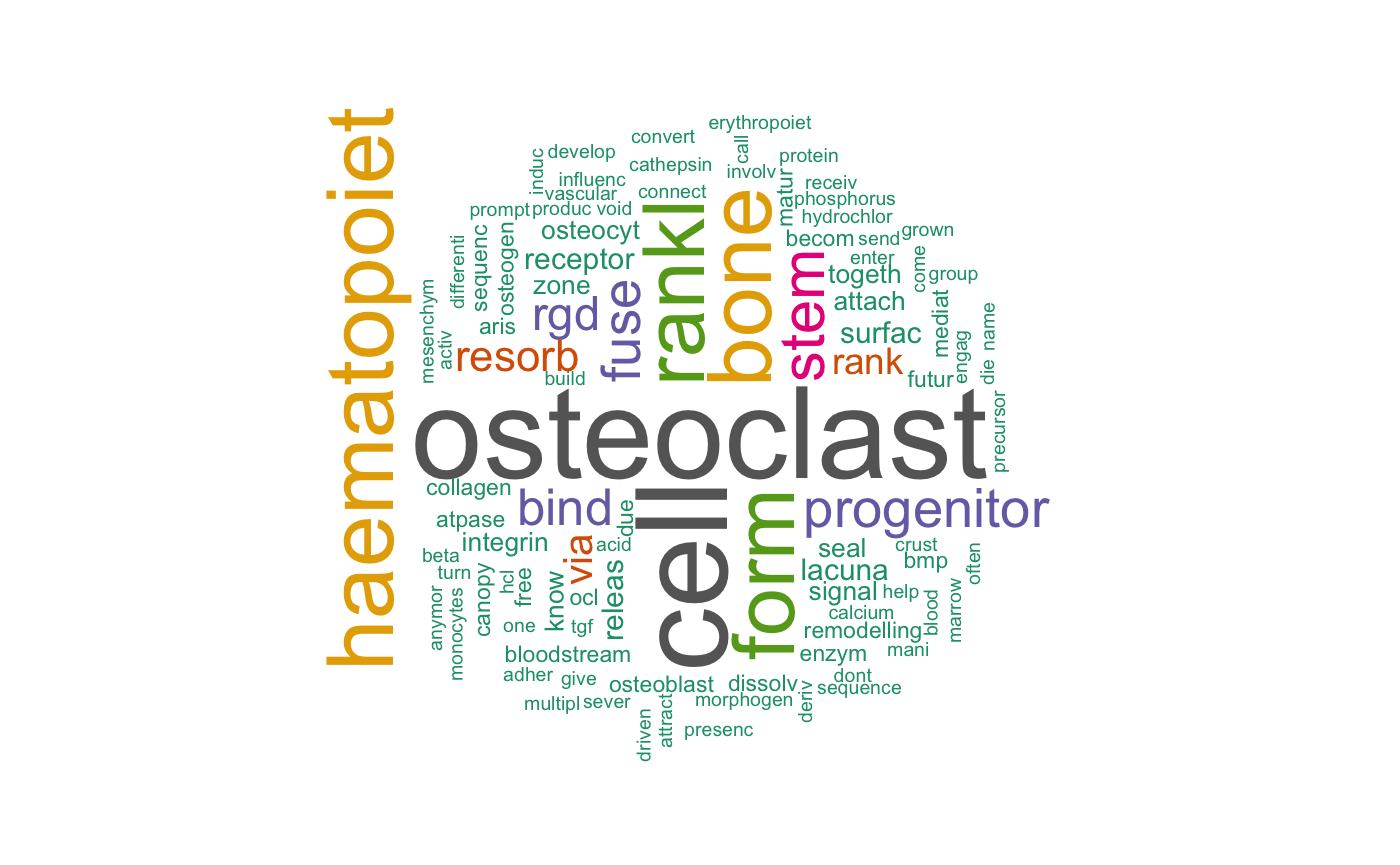

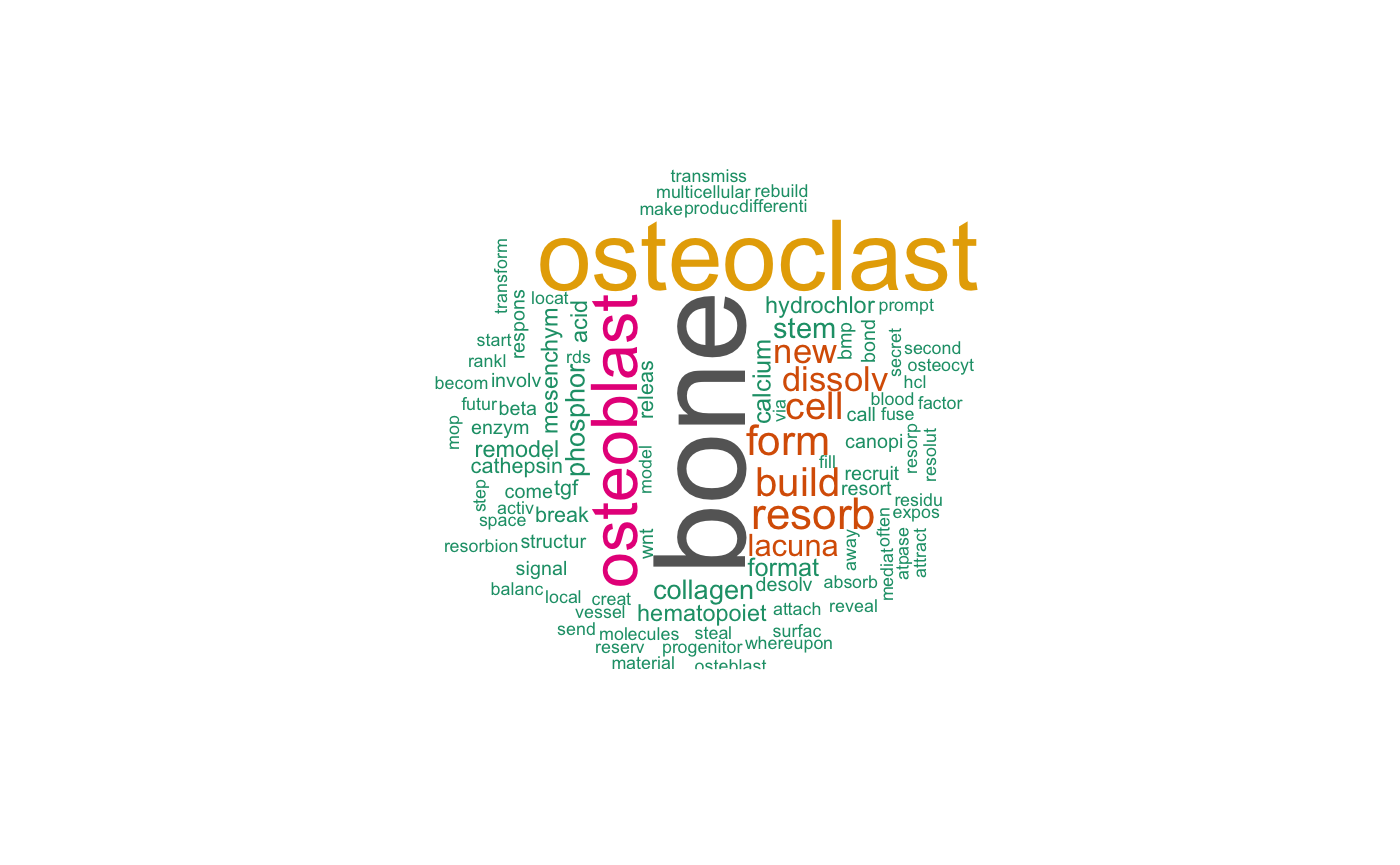

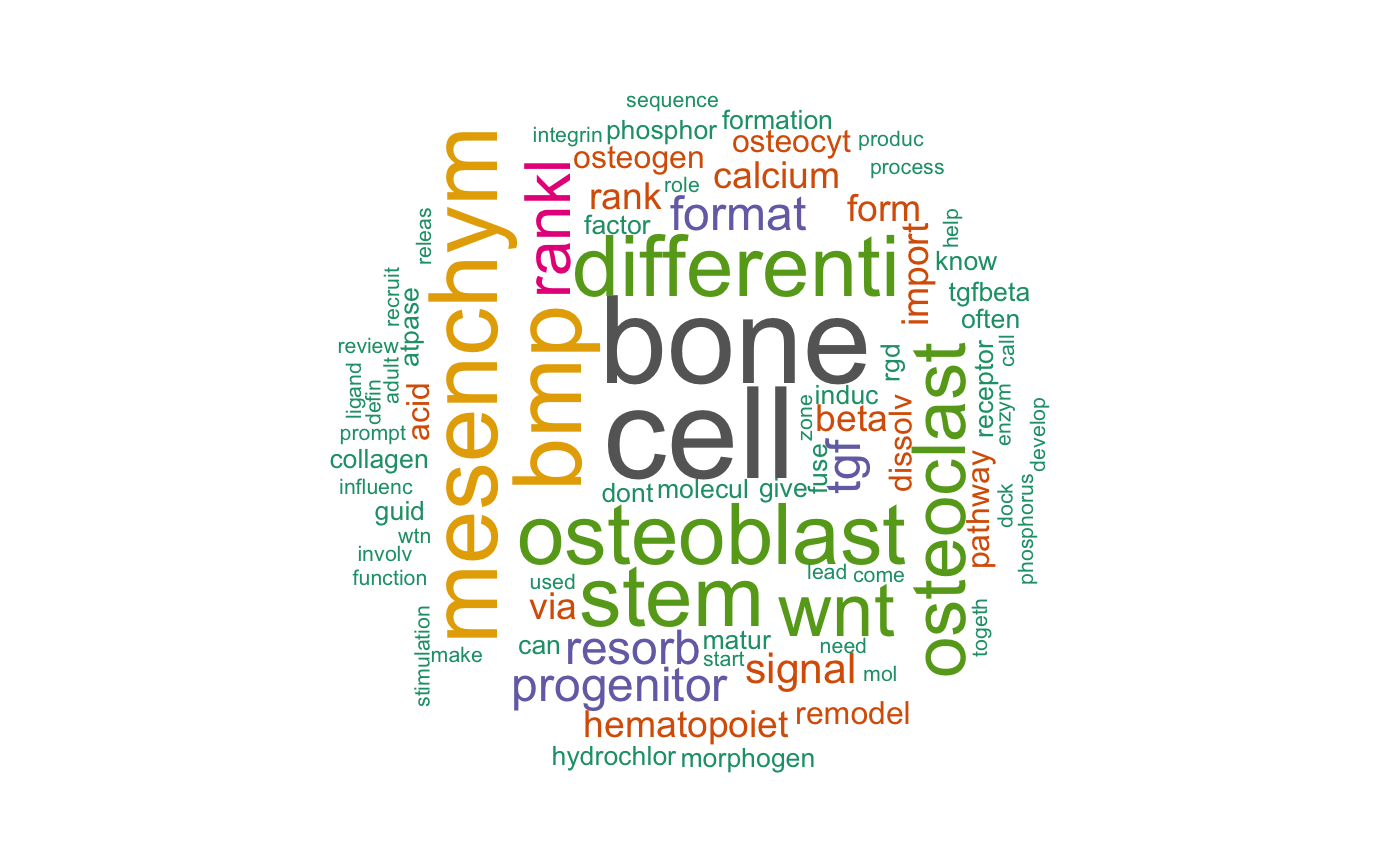


a) b) c)

Figure S24: Word cloud representation of text answers by students on the formation of osteoclasts (a), the functions of osteoclasts and osteoblasts (b), and the osteogenic differentiation (c).

### S9 Text Analysis


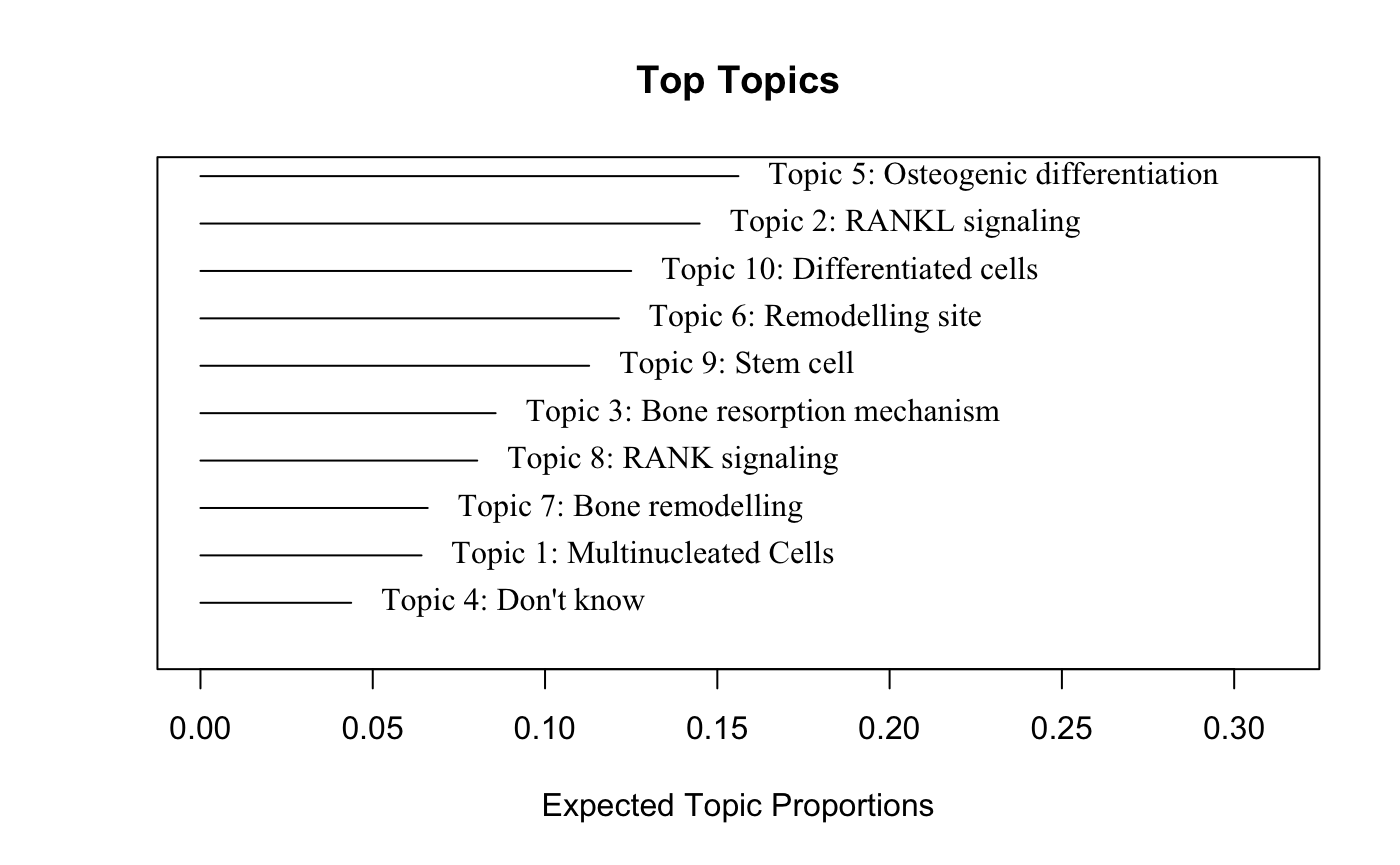


Figure S25: The distribution of topics as percent of the total text length across all texts (100%).

#### S9.1 Structural Topic Modelling

We tested the optimal number of topics according to Roberts et al [[55]](https://paperpile.com/c/3DGn7U/Zz72). We found that with more than 10 topics, the benefits decreased.

a)
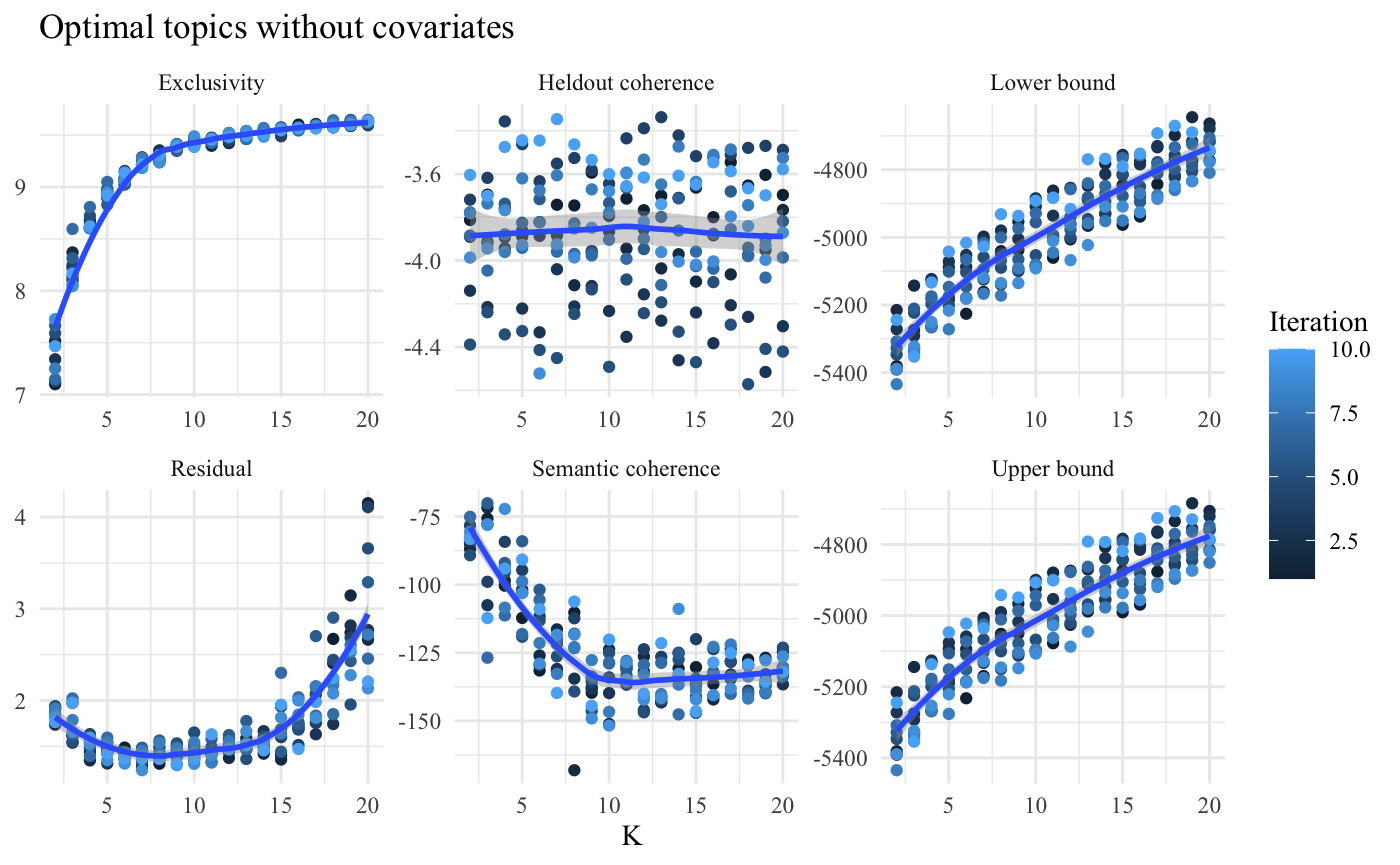


b)
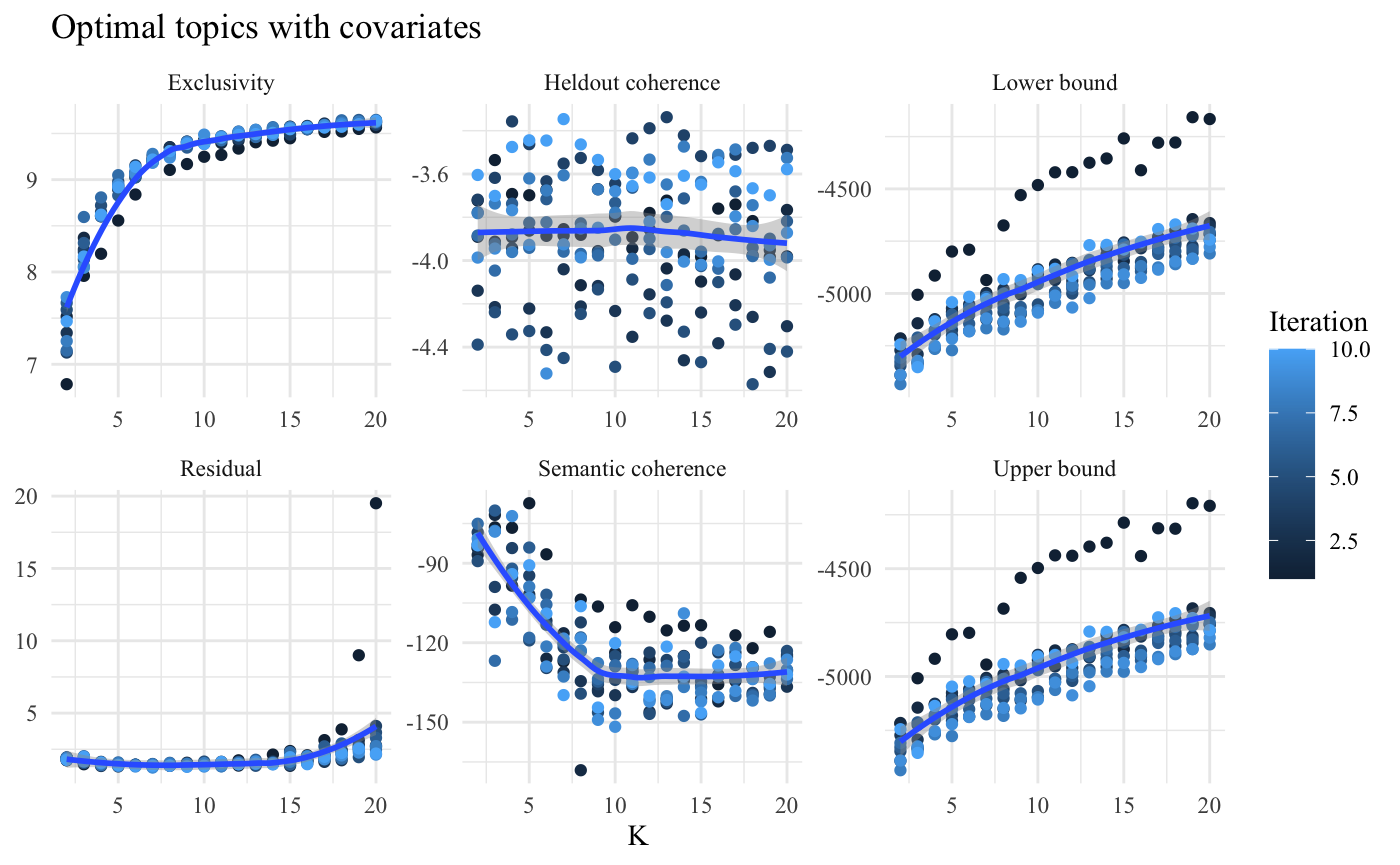


Figure S26: The quality of topic modeling across different numbers of topics. With (a) and without (b) covariates, there was an optimal tradeoff at around 10 topics (bend in blue line).

#### S9.2 Topic contrasts

These contrasts showed whether a word more strongly belonged to one topic or another [[55]](https://paperpile.com/c/3DGn7U/Zz72). A word more to the left indicated belonging to the left topic, a word more to right indicated belonging to the right topic. The size of the word indicated the certainty with which the word was placed on the scale. We used these contrasts as validation of our independent raters.

We observed that words that strongly belonged to several topics often sat in the middle of the scale (grey color) when these topics were compared, and firmly on their topics side otherwise. We could use this to differentiate the context. For instance, bone appeared strongly in topic 3,6, and 7. However, the additional words indicated dissolving bones, forming bones and resorbing bones.


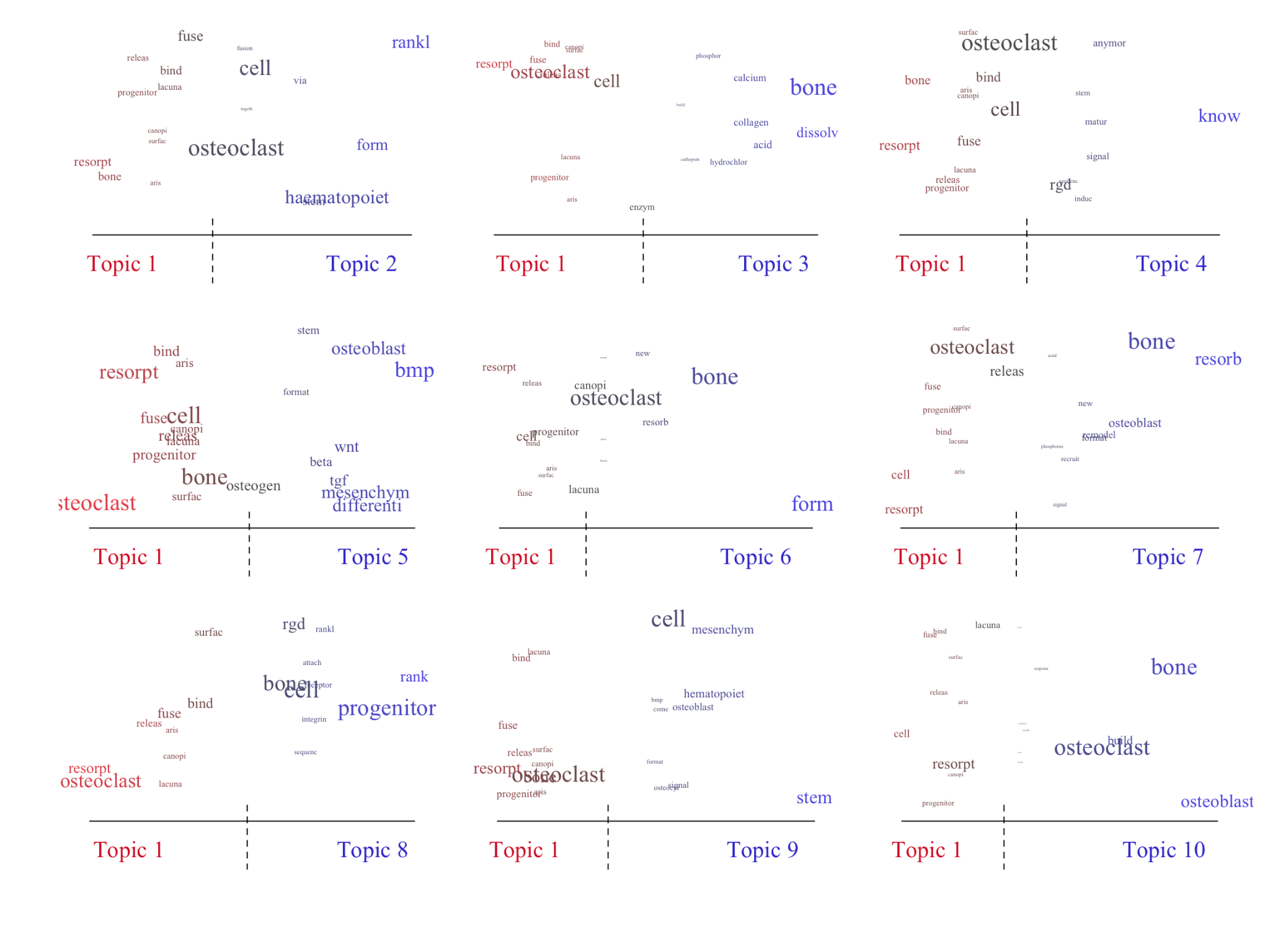


Figure S27: The keyword “resorption” and “osteoclast” differentiated topic 1 clearly from all other topics. The other words like “bind”, “fuse”, and “release” indicated the work of multinucleated cells.


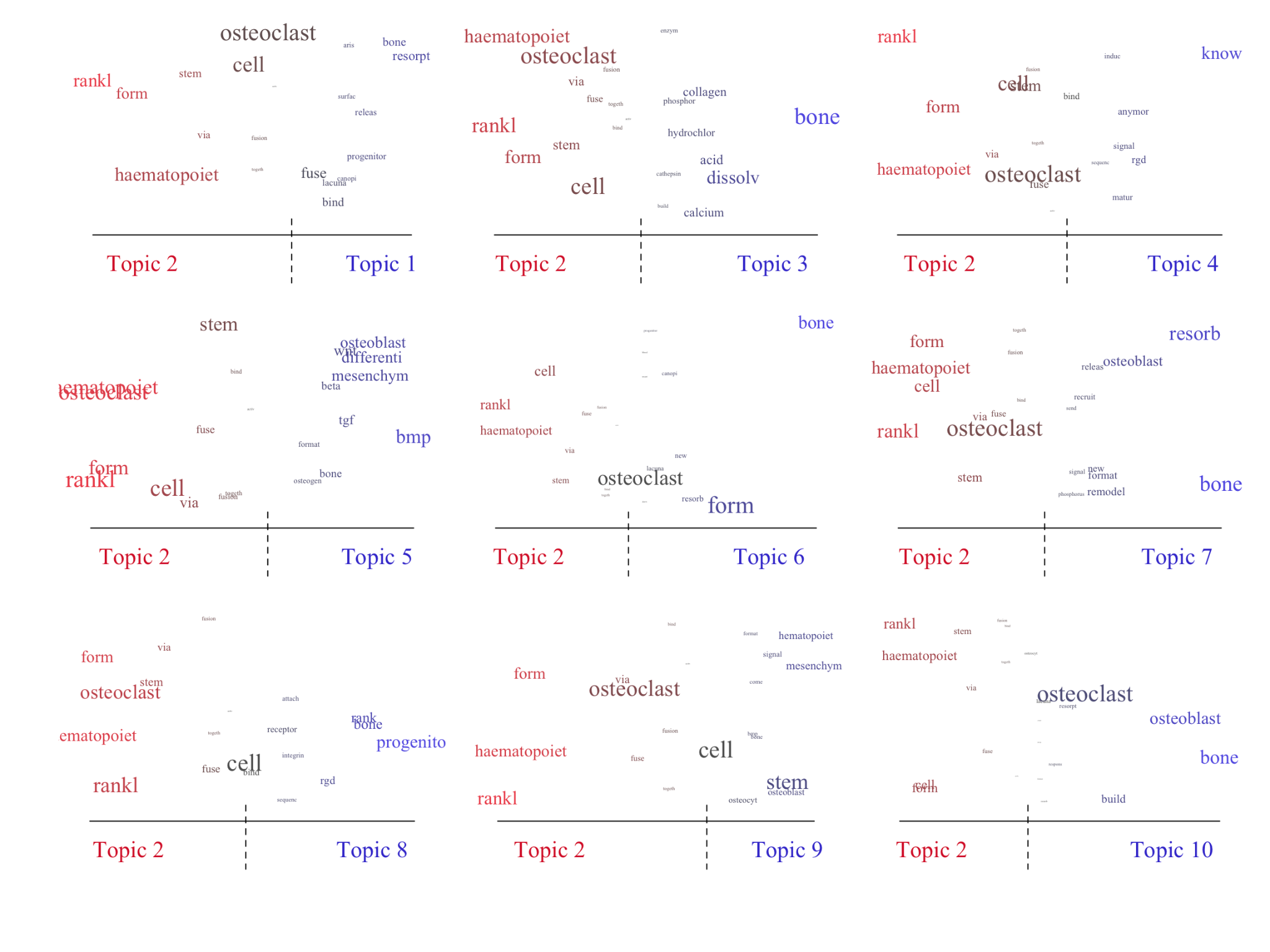


Figure S28: The keywords “rankl” and “haematopoietic” differentiated topic 2 clearly from all other topics.


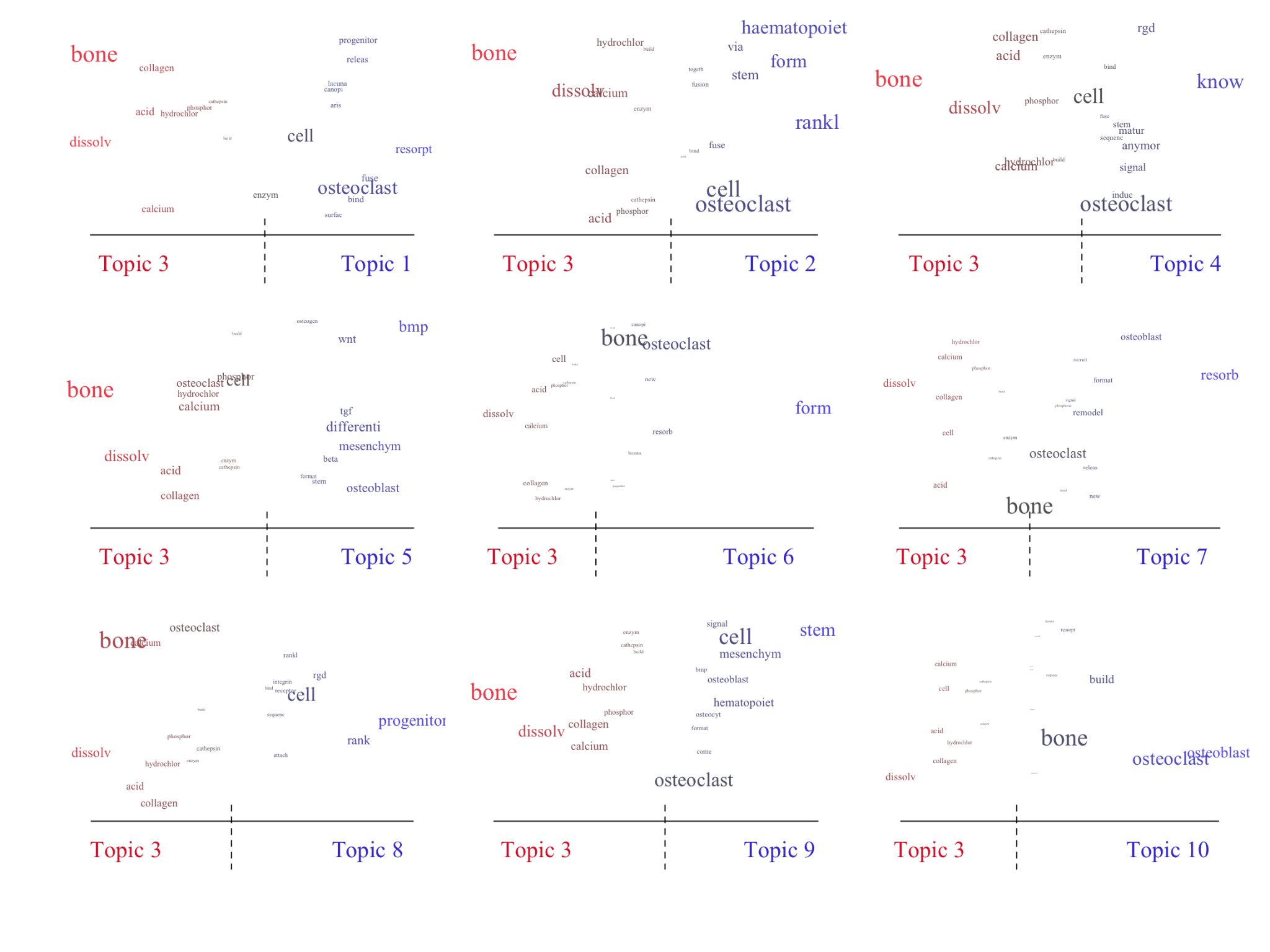


Figure S29: The keyword “bone” and “dissolv” differentiated topic 3 clearly from all other topics.


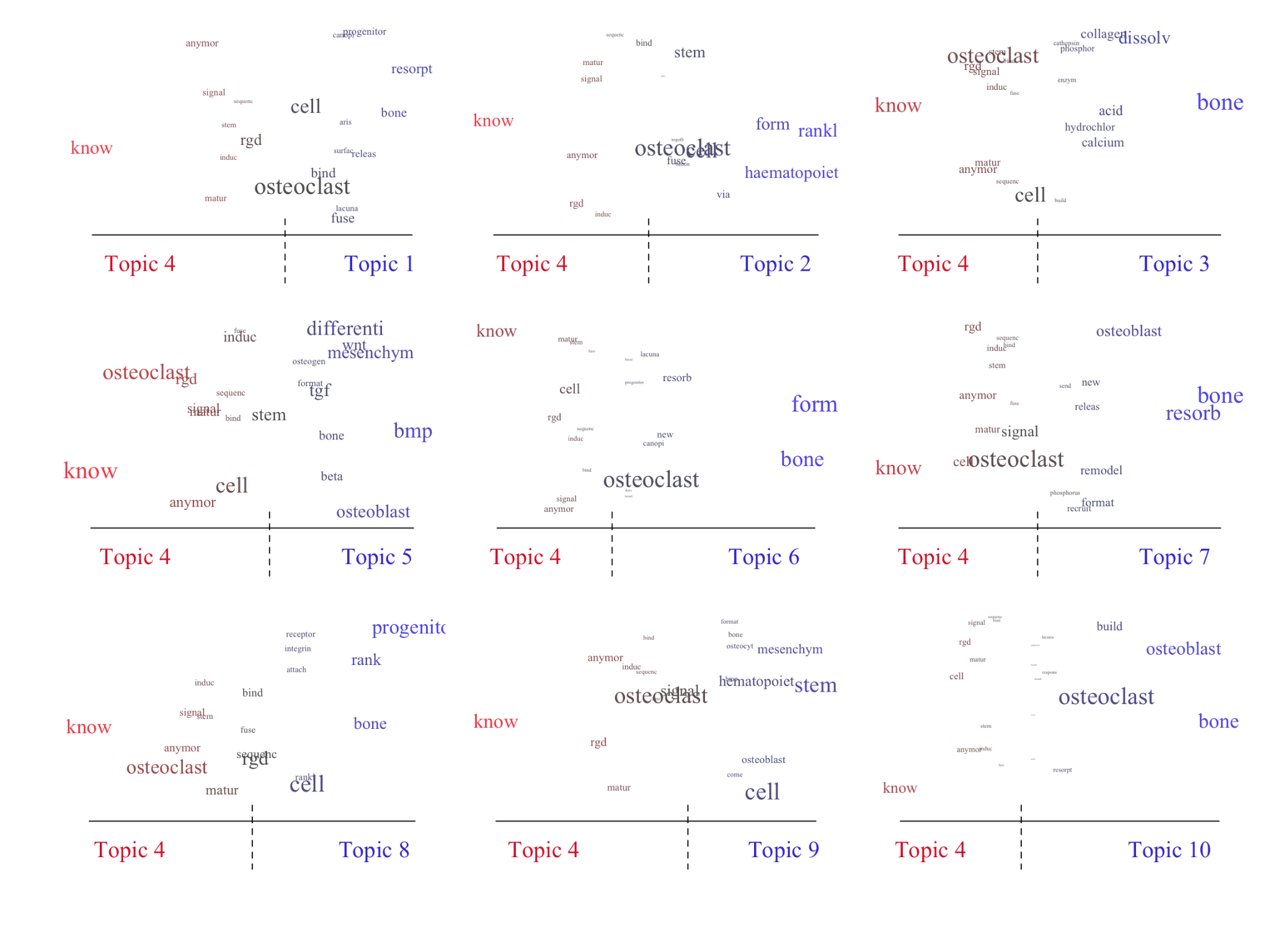


Figure S30: The keyword “know” differentiated topic 4 clearly from all other topics. This indicates that “don’t know” was the key driver of topic 4.


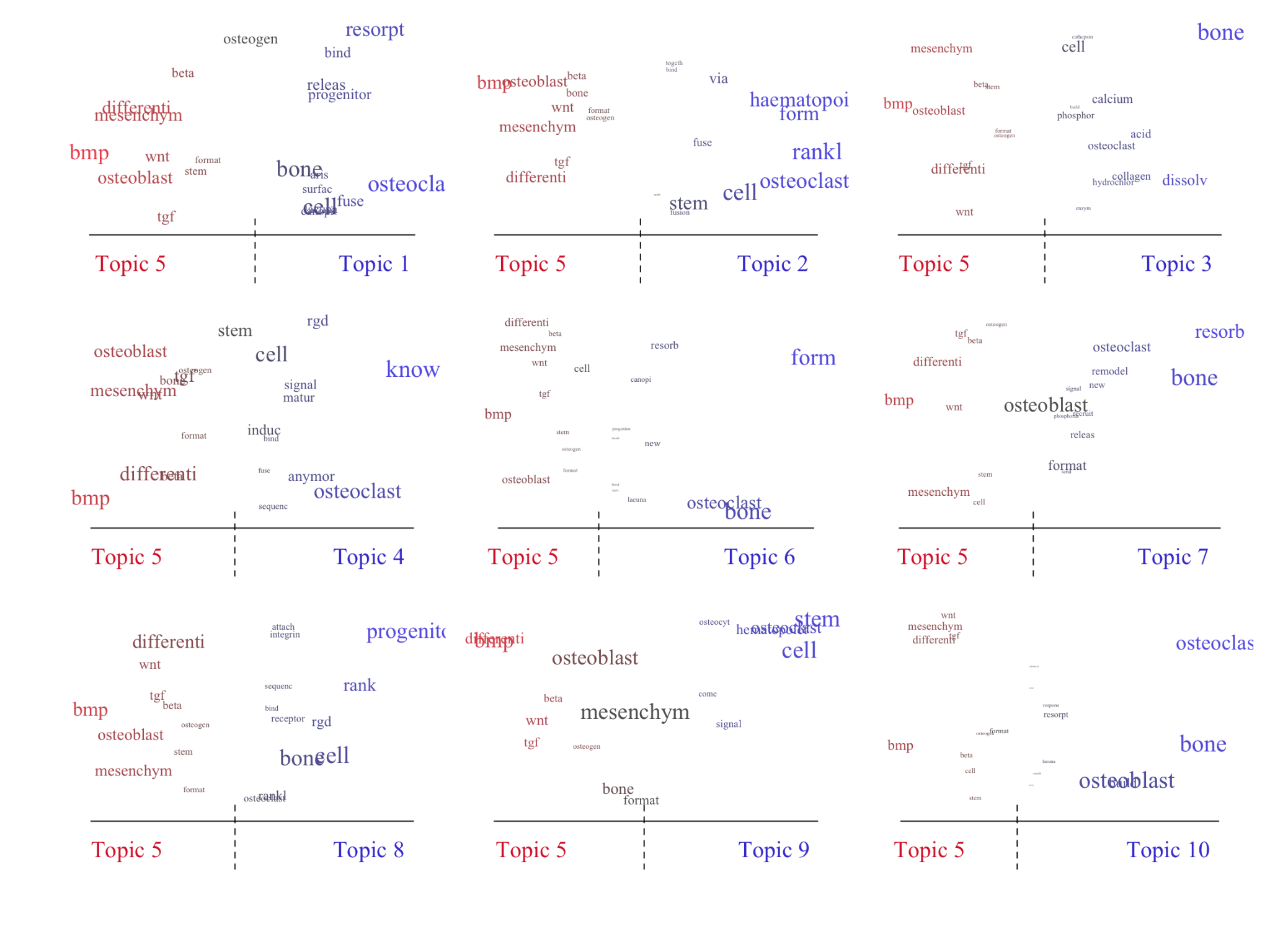


Figure S31: The keywords “bmp” and “differentiate” differentiated topic 5 clearly from all other topics.


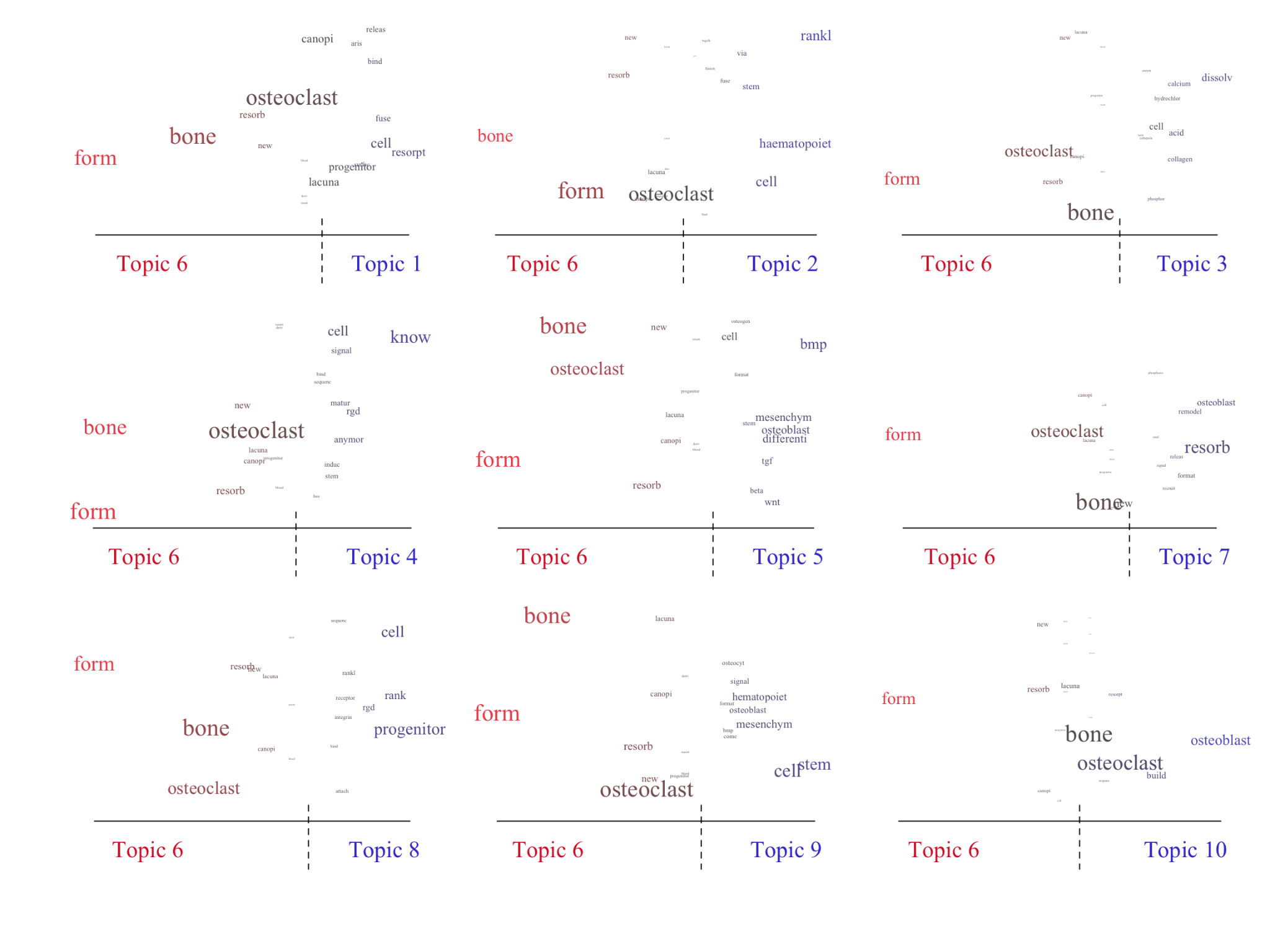


Figure S32: The keywords “bone” and “form” differentiated topic 6 clearly from all other topics.


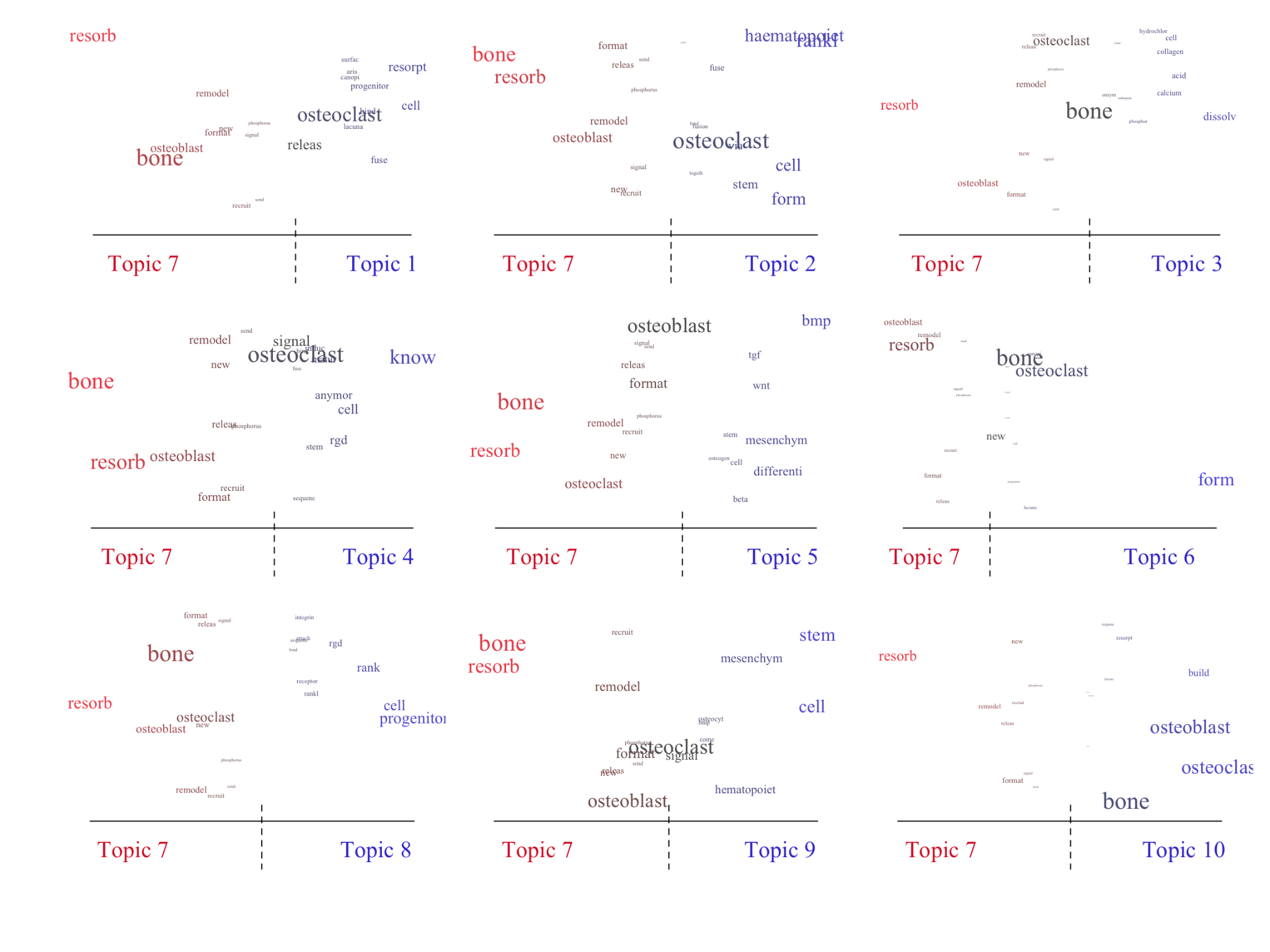


Figure S33: The keywords “bone” and “resorb” differentiated topic 7 clearly from all other topics.


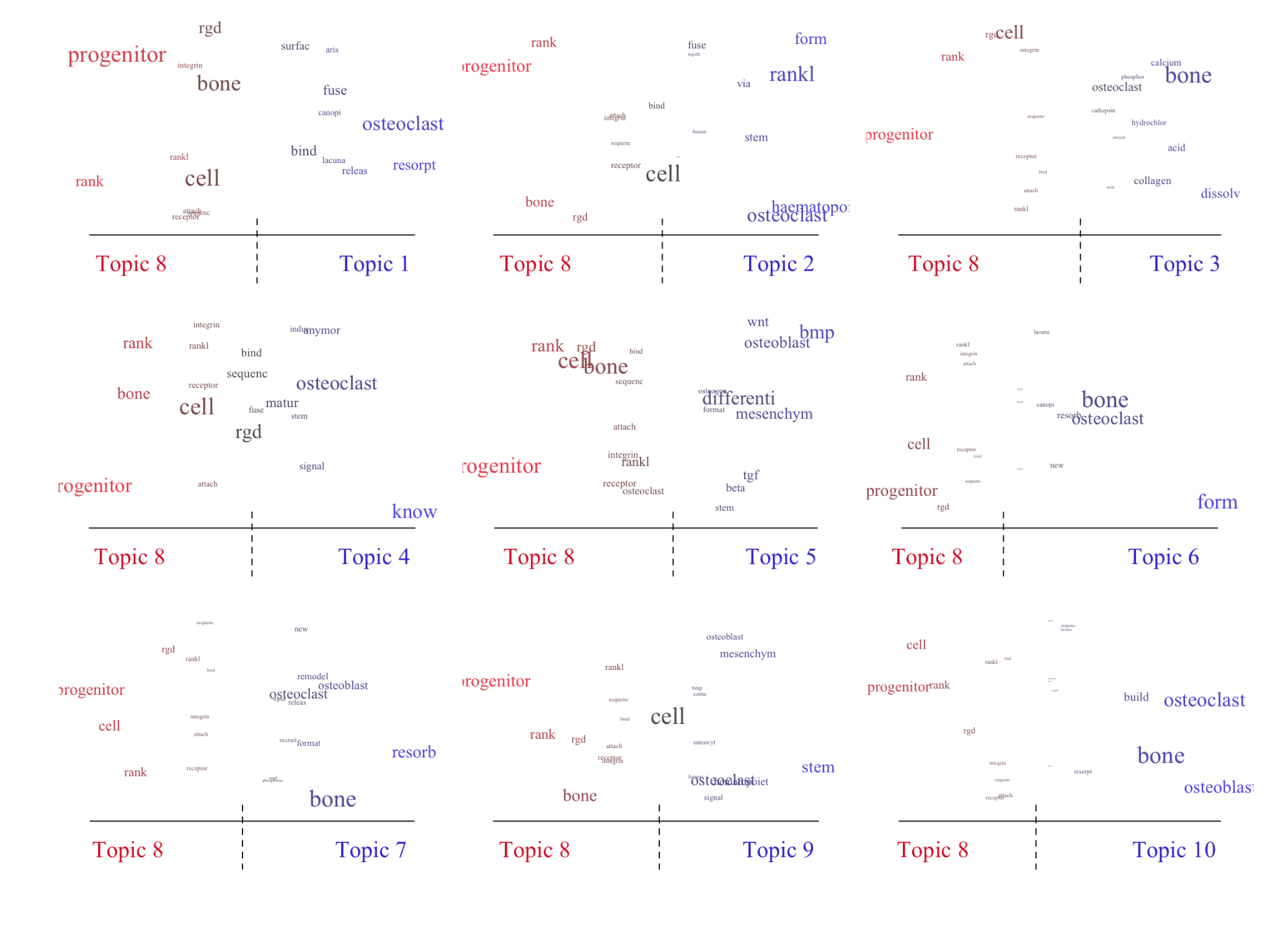


Figure S34: The keywords “progenitor” and “rank” differentiated topic 8 clearly from all other topics.


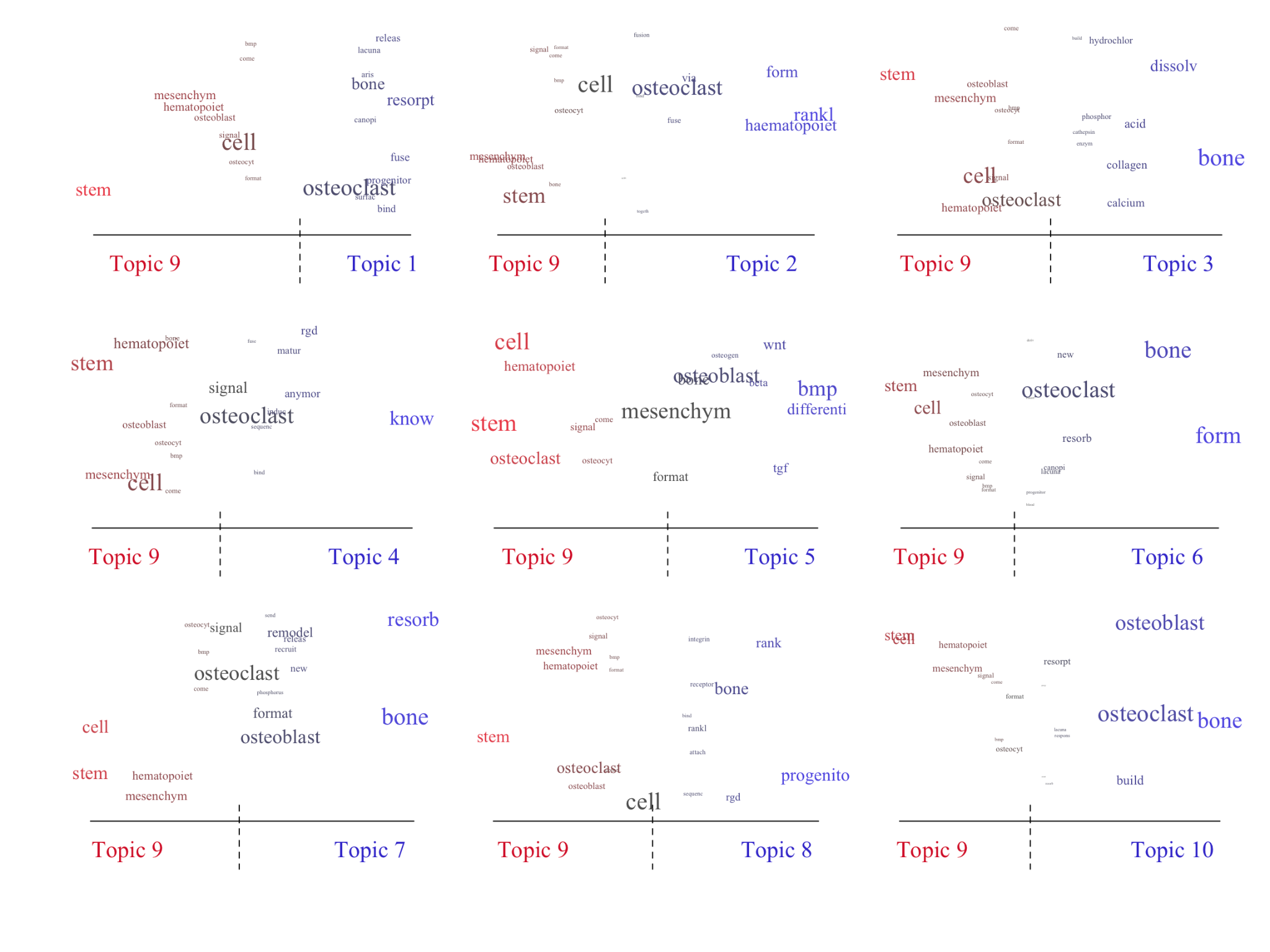


Figure S35: The keywords “stem” and “cell” differentiated topic 9 clearly from all other topics.


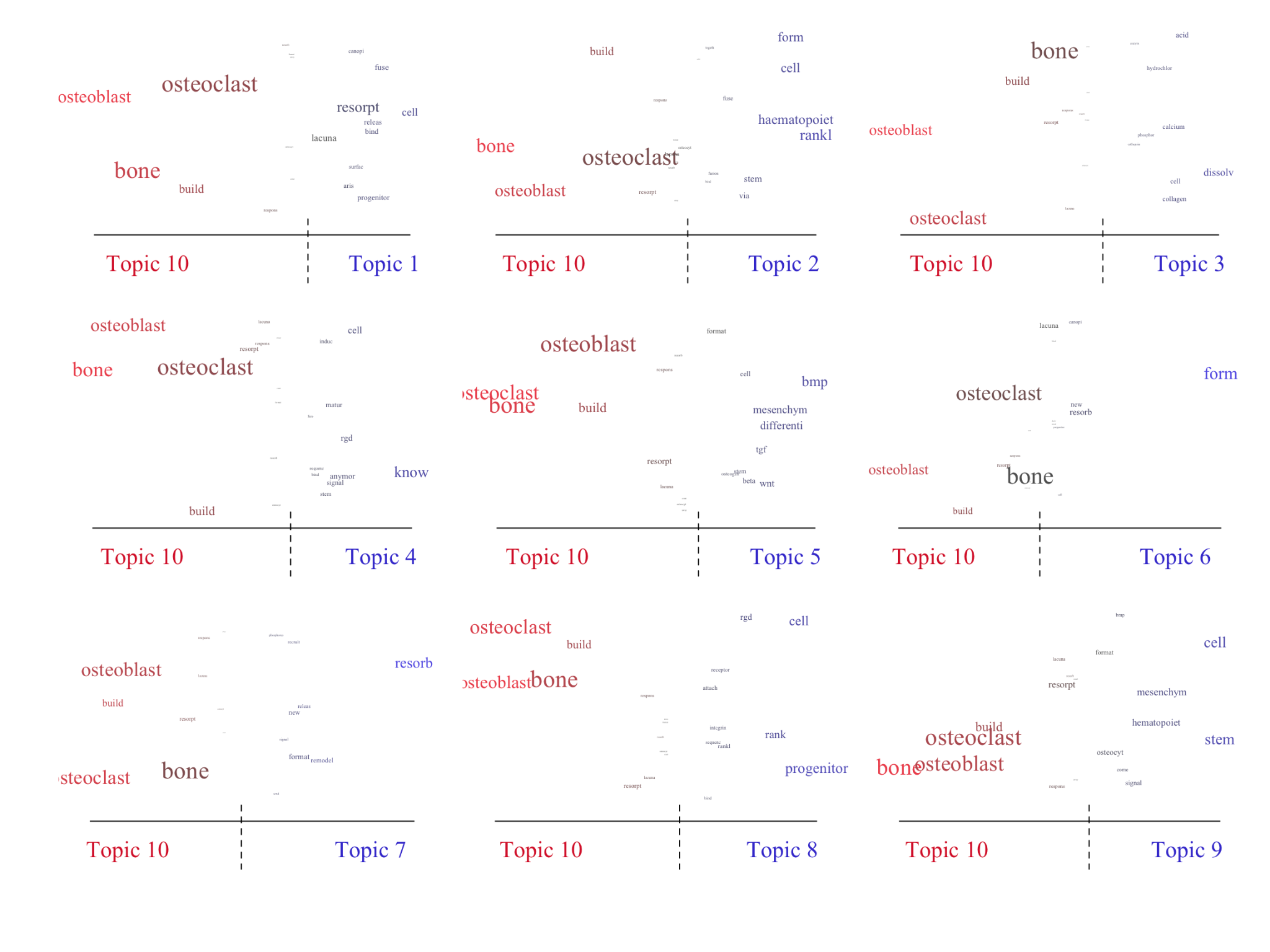


Figure S36: The keywords “build”, “bone”, “osteoclast” and “osteoblast” differentiated topic 3 clearly from all other topics.

#### S9.3 Treatments as Covariates

Table S2: The questions for the text analysis.

|  | Title | Question |
| --- | --- | --- |
|  |  | << |
| Q1 | Formation | *Describe the formation of an osteoclast in 3 to 6 sentences.* |
| Q2 | Function | *Describe the function of osteoclasts and osteoblasts in 3 to 6 sentences.* |
| Q3 | Differentiation | *Describe the processes and molecules involved in osteogenic differentiation, and the role they play, in 3 to 6 sentences.* |


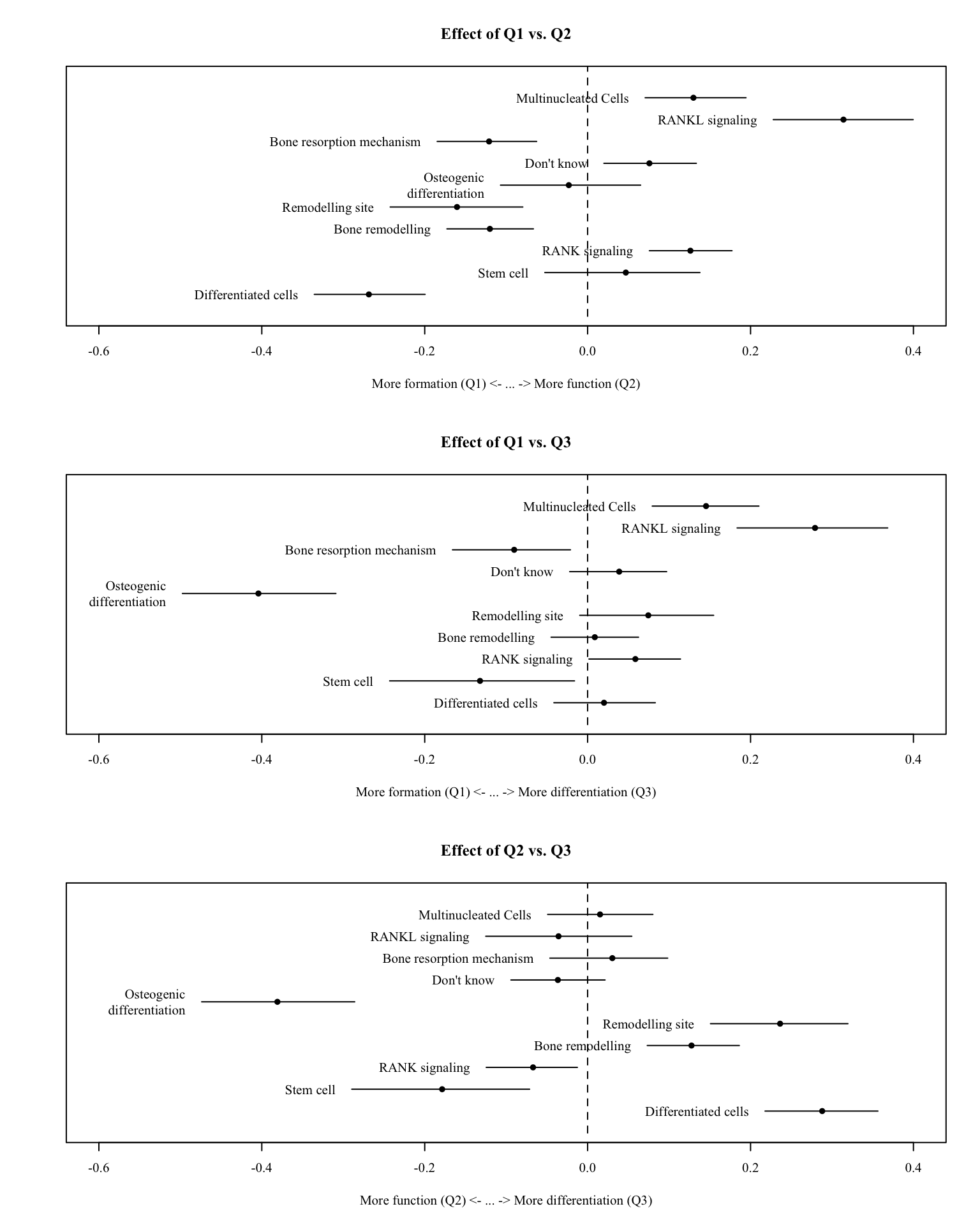


Figure S37: We also investigated whether topics belonged to specific questions. Following the methodology introduced in Figure 10, we found multiple topics were shared between two questions but not all three.

#### S9.4 Long-term observation

Lastly, we investigated how topic proportions changed over the long-term. Most topics remained on the same level over time but three topics exhibited a significant change over time (Figure S19). The RANK signaling topic (t= -2.195, *P* = .03) was mentioned more rarely whereas the remodeling side (t = 2.232, *P* = .017) and stem cells (t = 3.138, *P* = .002) were mentioned more often.


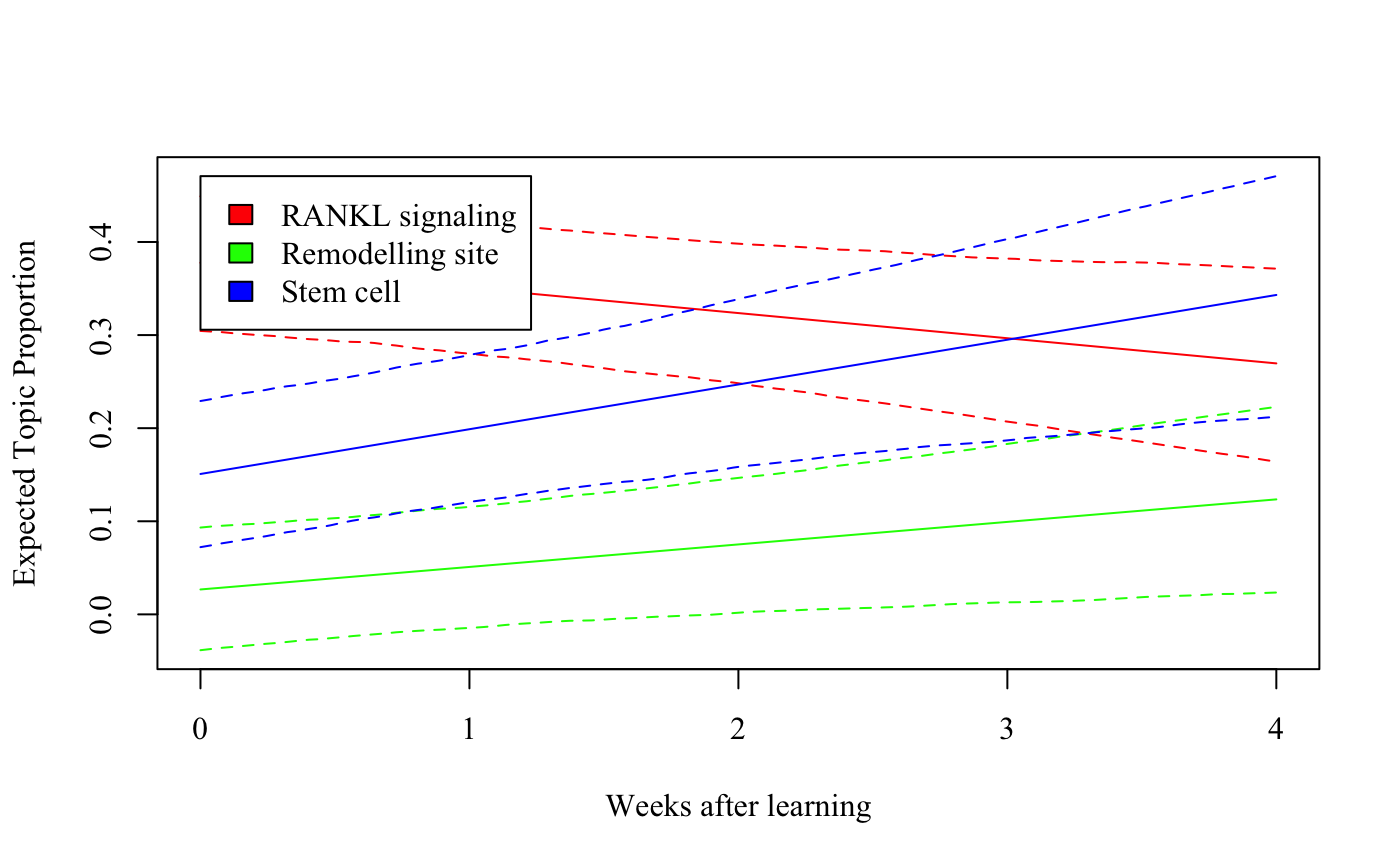


Figure S38: Topics over time. Most topics retained their proportion over time but the topics on RANKL decreased in prominence whereas the remodeling site and stem cell topics gained in prominence.

### C Intercoder reliability

The coder responses where first collected and then standardised to compute Krippendorff’s α, see Table S1. We highlighted in red when the wording was assimilated as the content was deemed to be similar. Topic 4 was removed from the calculation as the topic contrasts revealed that it gathered the “don’t know” responses.

Table S3: Intercoder-Reliability labels

|  | Rater 1 | Rater 2 | Rater 3 |
| --- | --- | --- | --- |
|  |  |  |  |
| **Topic1** | Cells | Bone Resorption | Multinucleated Cells |
| **Standardized** | Multinucleated Cells | Bone Resorption | Multinucleated Cells |
| **Topic2** | Progenitors | Osteoclast | Master Regulator RANKL |
| **Standardized** | Progenitors | Osteoclast | Master Regulator RANKL |
| **Topic3** | Bone Formation | Osteolyse | Bone Resorption Mechanism |
| **Standardized** | Bone Resorption Mechanism | Bone Resorption Mechanism | Bone Resorption Mechanism |
| **Topic4** | Signaling, Receptors | Development | Sealing zone |
| **Standardized** | Signaling, Receptors | Development | Sealing zone |
| **Topic5** | Growth Factors | Protein | Osteogenic differentiation |
| **Standardized** | Osteogenic differentiation | Protein | Osteogenic differentiation |
| **Topic6** | Bone Resorption | Bone Remodelling Compartment | Remodelling Site |
| **Standardized** | Bone Resorption | Remodelling Site | Remodelling Site |
| **Topic7** | Bone Remodelling | Bone Remodelling | Bone Remodelling |
| **Standardized** | Bone Remodelling | Bone Remodelling | Bone Remodelling |
| **Topic8** | Bone Receptors | Differentiation | Rankl-Rank Signaling |
| **Standardized** | Rankl-Rank Signaling | Differentiation | Rankl-Rank Signaling |
| **Topic9** | Hematopietic cell | Hematopietic cell | Stem Cell origin |
| **Standardized** | Stem Cell origin | Stem Cell origin | Stem Cell origin |
| **Topic10** | Bone Remodelling | Communication | Resorption Lacuna |
| **Standardized** | Resorption Lacuna | Communication | Resorption Lacuna |

### E IMMS all responses


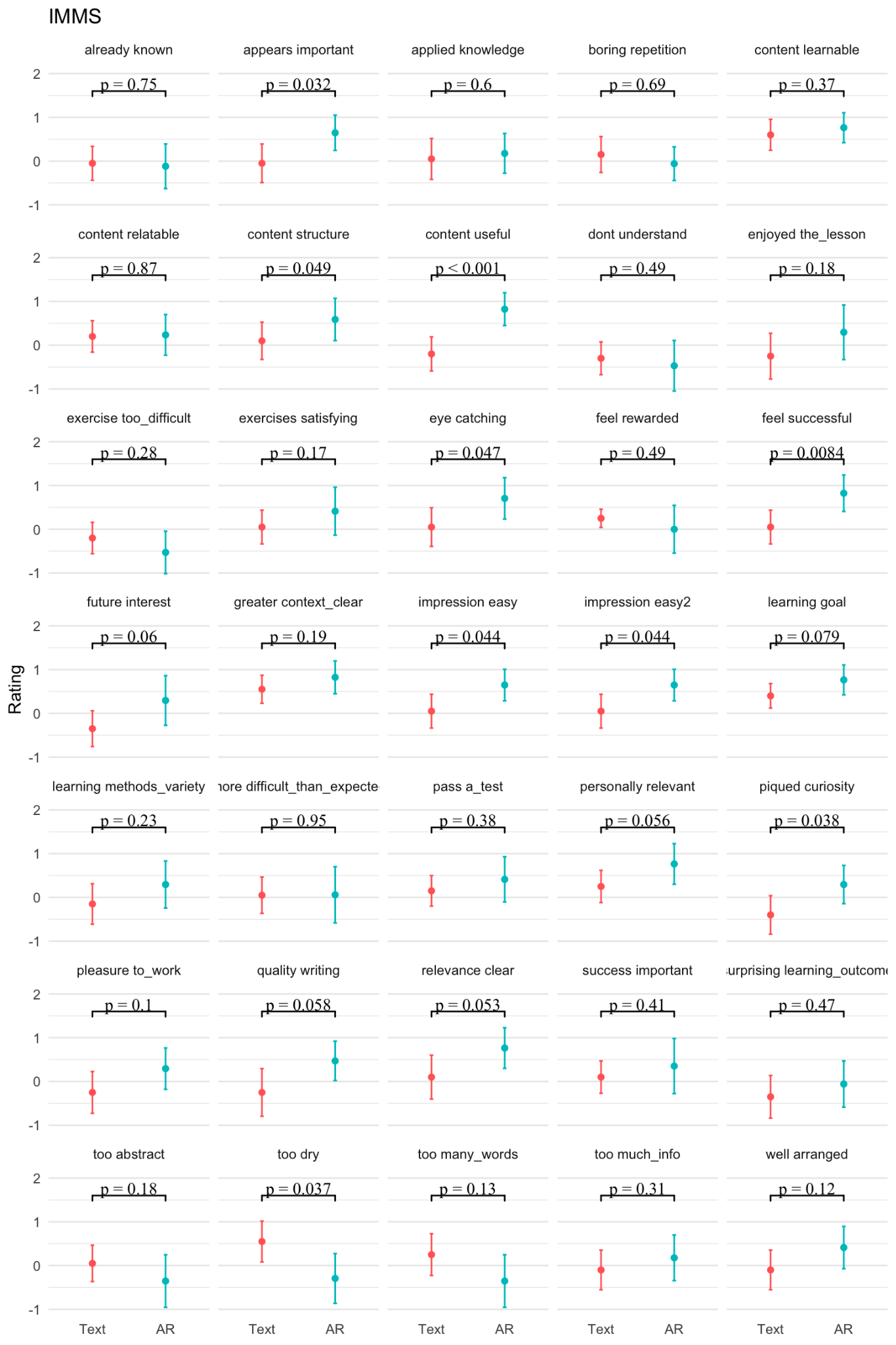


Figure S39: Responses to all IMMS questions. NS stands for “no significance”.
